# Scaling behaviour for the water transport in nanoconfined geometries

**DOI:** 10.1038/ncomms4565

**Published:** 2014-04-03

**Authors:** Eliodoro Chiavazzo, Matteo Fasano, Pietro Asinari, Paolo Decuzzi

**Affiliations:** 1 Department of Energy, Politecnico di Torino, Corso Duca degli Abruzzi 24, Torino 10129, Italy; 2 Department of Translational Imaging, The Methodist Hospital Research Institute, 6670 Bertner Ave, Houston, Texas 77030, USA; 3 Department of Nanomedicine, The Methodist Hospital Research Institute, 6670 Bertner Ave, Houston, Texas 77030, USA; 4 Nanofabrication Department, Italian Institute of Technology, Via Morego 30, Genova 16163, Italy; 5 These authors contributed equally to this work

## Abstract

The transport of water in nanoconfined geometries is different from bulk phase and has tremendous implications in nanotechnology and biotechnology. Here molecular dynamics is used to compute the self-diffusion coefficient *D* of water within nanopores, around nanoparticles, carbon nanotubes and proteins. For almost 60 different cases, *D* is found to scale linearly with the sole parameter *θ* as *D*(*θ*)=*D*_*B*_[1+(*D*_*C*_/*D*_*B*_−1)*θ*], with *D*_B_ and *D*_C_ the bulk and totally confined diffusion of water, respectively. The parameter *θ* is primarily influenced by geometry and represents the ratio between the confined and total water volumes. The *D*(*θ*) relationship is interpreted within the thermodynamics of supercooled water. As an example, such relationship is shown to accurately predict the relaxometric response of contrast agents for magnetic resonance imaging. The *D*(*θ*) relationship can help in interpreting the transport of water molecules under nanoconfined conditions and tailoring nanostructures with precise modulation of water mobility.

## Abstract (web summary)

A precise control of water transport in different configurations is crucial for many technological applications. Chiavazzo *et al.* define a dimensionless scaling parameter to fully describe the water self-diffusion coefficient in various nanoconfined geometries using molecular dynamic simulations.

## Graphical abstract

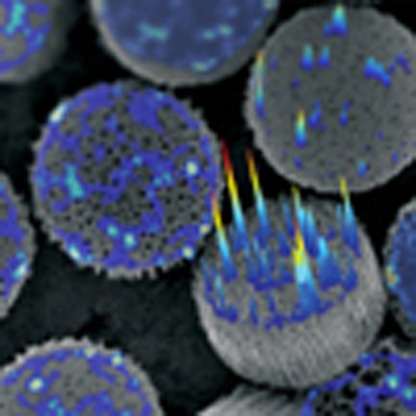

Despite its fundamental importance in science and technology, the physical and transport properties of water are far from being completely understood^1^. The self-diffusion of water molecules *D* in proximity of solid surfaces, at the interface between immiscible liquids, and in confined geometries, such as nanopores and nanotubes, is a very different process as compared to the bulk phase^2^,^3^,^4^. The thermal agitation of the water molecules in the bulk liquid is only dictated by the local temperature and pressure conditions, and molecular diffusion follows the Einstein relation^5^. Differently, under confined conditions, the mobility of water molecules is perturbed by the presence of additional interaction forces arising at the water/solid interfaces, mainly van der Waals and Coulomb interactions. These additional forces usually reduce the local molecular diffusion^6^,^7^. Even if considerable work has been done in recent years, both experimentally and theoretically, to understand and characterize the perturbed behaviour of the water molecules in confined geometries, there is still no complete comprehension of the process and often the published results are contradictory^8^.

Controlling the mobility of water molecules is of relevance to several scientific disciplines and has implications in multiple technological applications. For instance, water adsorption/desorption in nanoporous materials, such as zeolites, has potential in long-term thermal storage and energy engineering^9^,^10^; filters with nanopores and nanochannels are increasingly explored for their large surface area and higher efficiency^11^,^12^; in heat transfer problems, nanofluids are under investigation because of their peculiar thermal properties^13^,^14^; in micro/nanotechnology processes, controlling the deposition and surface diffusion of water molecules is critical for precise manufacturing^15^,^16^; in biology, the mechanisms regulating the transport of single water molecules through cell membrane channels (aquaporins) and the multi-scale water compartmentalization in tissues are still elusive^17^,^18^,^19^,^20^. Also, proteins tend to modify their structure and function according to the surrounding aqueous environment^21^,^22^.

Certainly, nanomedicine is one of the fields where several exciting discoveries and technological applications can be directly related to the anomalous behaviour of water in confined geometries. A few examples are the enhancement in longitudinal relaxivity associated with the entrapment of Gd^3+^-ion complexes in mesoporous structures^23^,^24^; the dynamics of water molecules in nanotubes and nanochannels for controlled drug delivery^25^,^26^; and the design of hydrogel-based nano/microparticles^27^,^28^. In particular, the dynamics of water molecules is essential in magnetic resonance imaging (MRI), in that contrast enhancement is influenced by the local diffusion of water molecules^29^,^30^. It is known that for paramagnetic metal complexes, such as Gd^3+^ ions, the Solomon–Bloembergen–Morgan theory^31^ would predict a change in longitudinal relaxivity *r*_1_ of the complex following a variation in the relative translational diffusion time (*τ*_*D*_) of the water molecules surrounding the complex, and in the residence lifetime (*τ*_M_) of the water molecules bound to the complex. Similarly, for magnetic nanoparticles (NPs), such as the iron oxide NPs, an increase in *τ*_*D*_ (that is, decrease in *D*) would enhance the transversal relaxivity *r*_2_ (ref. 32). Hence, the modulation and precise control of the diffusion of the water molecules in the vicinity of an MRI contrast agent plays an important role in imaging performance. This concept has been already successfully proved by experiments^23^, but a clear rationale (and a computationally efficient tool) for optimally designing such agents is still missing.

In this work, the self-diffusion coefficient *D* of water molecules is investigated through molecular dynamics (MD) simulations under five different isothermal configurations, namely, within silica (SiO_2_) nanopores, around spherical hydroxylated NPs, within SiO_2_ nanopores filled by NPs, around single-wall carbon nanotubes (CNTs) and proteins. The coefficient *D* has been estimated for almost 60 cases by varying the size of the NPs and nanopores, the electrostatic surface charges and level of hydrophobicity, as well as the type of protein. The self-diffusion coefficient *D* for all different configurations has been found to scale with a single non-dimensional parameter *θ*, incorporating both geometrical and physicochemical information, following the relationship *D*(*θ*)=*D*_B_[1+(*D*_C_/*D*_B_−1)*θ*]. The *D*(*θ*) scaling is modulated by the coefficients *D*_B_ and *D*_C_, which represent the bulk and totally confined diffusion of water, respectively. This *D*(*θ*) law has been applied to estimate the enhancement in MRI contrast in magnetic nanoconstructs obtained by geometrically confining super-paramagnetic iron oxide NPs (SPIOs) into silicon mesoporous matrices. It has been confirmed that the transversal relaxivity of SPIOs can be significantly augmented by modulating the diffusion of water molecules. This law would help in explaining and rationalizing previous experimental evidences^23^, and represent a ready-to-use tool for the rational design of nanoconstructs based on the nanoscale confinement of water molecules.

## Results

### Computing the diffusion of nanoconfined water molecules

**Figure 1:**
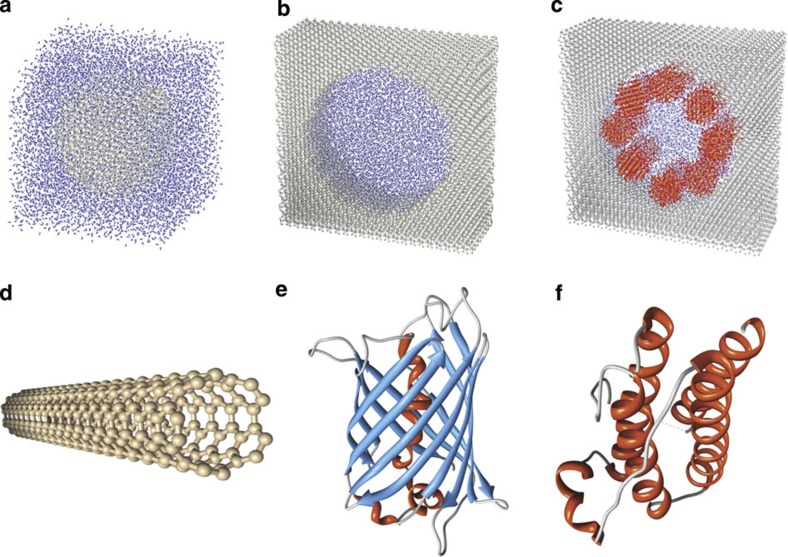
Selected configurations. (**a**) SiO_2_ particle in water, diameter *φ*=5.2 nm (blue dots: water molecules; grey dots: SiO_2_ atoms); (**b**) SiO_2_ nanopore filled by water, diameter Φ=8.1 nm; (**c**) sixteen Fe_3_O_4_ NPs within a SiO_2_ nanopore filled by water, φ=2.0 nm and Φ=8.1 nm (red and cyan dots: Fe_3_O_4_ atoms); (**d**) single-walled CNT with chirality (5,5); (**e**) green fluorescence protein; (**f**) leptin protein (the standard ribbon visualization of secondary structures has been used for proteins). In **d**–**f** water molecules have been removed for clarity. Almost 60 different cases have been analysed by varying the size and surface properties of the NPs, nanopores and nanotubes as well as the type of protein.

MD simulations were used to compute the self-diffusion coefficient *D* of water molecules confined under different configurations. These are shown in Fig. 1 and include the case of water molecules (blue dots) moving (a) around spherical hydroxylated nanoparticles (NPs) (grey dots); (b) within a hydrated nanopore (grey dots); (c) around hydroxylated NPs (red dots) adsorbed on the surface of a hydrated nanopore (grey dots); (d) around and within single-walled carbon nanotubes (CNTs); (e,f) around proteins. The NPs are made out of magnetite (Fe_3_O_4_) crystals (red and cyan dots), with OH^−^ functional groups on their surface, or SiO_2_ crystals (grey dots), with silanol SiOH functional groups on the surface. The nanopores are made out of SiO_2_ only.

To investigate the influence of geometry and material properties, the self-diffusion coefficient *D* of the water molecules was computed for 58 different cases. In particular, these cases were different in terms of NP diameter, being 1.3, 2.0 or 5.2 nm; nanopore diameter, with values 2.0, 4.1, 8.1 and 11.0 nm; number of NPs adsorbed on the nanopore wall, varying from 0 to 66 NPs per pore; CNT armchair chirality, namely, (5,5), (10,10), (20,20) and (30,30); and type of proteins, including molecules with a spherical (for example, ubiquitin) and elongated (for example, Ca^2+^-ATPase) shapes; of small (for example, 562 atoms of B1-immunoglobulin-binding domain) and large (for example, 9667 atoms of Ca^2+^-ATPase) sizes; and exhibiting a catalytic (for example, glucokinase), hormonal (for example, leptin) and transport (for example, myoglobin) function. As per the material properties, two types of interactions were considered in the MD simulations: bonded interactions and non-bonded interactions between the water molecules and the solid surfaces, described via van der Waals and Coulomb potentials. In the SiO_2_ structures and spherical NPs, the bonded interactions are modelled by means of harmonic terms. For the NPs, the strength *ε* of the Lennard–Jones potential was varied from 2.49 to 24.94 kJ mol^−1^ and the partial electrostatic charges of atoms were set to either the nominal value or zero for NPs and nanopores. For simulations with CNTs, the non-bonded interactions between CNTs and water molecules were modelled by the Lennard–Jones potential, with neutral carbon atoms and *σ*_CC_=0.36 nm, *ε*_CC_=0.29 kJ mol^−1^ (ref. 33). Finally for the proteins, all bonded and non-bonded interactions were modelled using the GROMOS96 43a2 force field^34^, which has been widely used for studying similar applications^35^,^36^. Finally, various hydration levels were considered providing an overall water density ranging from 715 to 941 kg m^−3^. Note that for these density values, there is no heterogenous wetting and consequently no anomalous behaviour related to low water-filling regimes^3^,^7^.

All computed values of the self-diffusion coefficient *D* are reported in the Supplementary Tables 1–11 and Supplementary Note 1. In general, it is observed that the coefficient *D* decreases compared with the bulk value, 2.60 × 10^−9^ m^2^ s^−1^ at 300 K (ref. 37), as the ratio between the total area of the solid–liquid interface and the total volume occupied by the water (*V*_w_) increases. More specifically, for a SiO_2_ nanopore, *D* is reduced from 2.50±0.09 × 10^−9^ m^2^ s^−1^ to 0.82±0.22 × 10^−9^ m^2^ s^−1^ as the pore diameter decreases from 11.0 to 2.0 nm. Moreover, *D* is inversely proportional to the NP concentration and diameter. For a 5.2-nm SiO_2_ NP, *D* decreases from the almost unconfined value to 2.12±0.04 × 10^−9^ m^2^ s^−1^ following a 36% increase in NP concentration. Indeed, the increase in NP concentration is associated with a decrease in separation distance between adjacent NPs and, consequently, a decrease in the volume available to water molecules. Consistently, *D* reduces as the concentration of Fe_3_O_4_ NP increases within a nanopore: *D* is 2.20±0.10 × 10^−9^ m^2^ s^−1^ in an 8.1-nm SiO_2_ nanopore; however, if 2 Fe_3_O_4_ NPs, of 2.0 nm in diameter, are adsorbed on the nanopore surface, *D* decreases to 2.07±0.14 × 10^−9^ m^2^ s^−1^ and it drops to 0.44±0.05 × 10^−9^ m^2^ s^−1^ (~80% decrease), if 16 Fe_3_O_4_ NPs, of 2.0 nm in diameter, are added in the nanopore. In addition, for 16 Fe_3_O_4_ NPs adsorbed on the wall of an 8.1-nm nanopore, *D* is 1.46±0.09 × 10^−9^ m^2^ s^−1^ for 1.3 nm NPs and becomes 0.44±0.05 × 10^−9^ m^2^ s^−1^ (~70% decrease) for 2.0 nm NPs. A similar trend is observed for the CNTs and proteins by reducing the size of the water box. More specifically, around the (5,5) chirality CNT with a length of 5 nm, the water diffusivity *D* decreases from the bulk value (box of 316 nm^3^) to 1.22±0.12 × 10^−9^ m^2^ s^−1^ (box of 21 nm^3^). Similarly, around the B1-immunoglobulin-binding domain, the diffusivity *D* decreases from 2.41±0.04 × 10^−9^ m^2^ s^−1^ (box of 348 nm^3^) to 0.87±0.10 × 10^−9^ m^2^ s^−1^ (box of 23 nm^3^). As expected, the above data qualitatively demonstrate that the self-diffusion coefficient *D* of water is strongly correlated to the ratio between the interface surface and the total water volume: the larger this ratio the smaller the water mobility. However, the possible contribution of other parameters should also be assessed.

To this end, sensitivity analyses were performed to elucidate the effect of the Lennard–Jones potential strength *ε* and Coulomb interactions on *D*. Larger values of the parameter *ε* are associated with lower mobility of the water molecules. As an example, let us consider the case of eight Fe_3_O_4_ NPs (2.0 nm diameter) adsorbed on the walls of an 8.1-nm diameter SiO_2_ nanopore. A one order of magnitude decrease of *ε* of Fe_3_O_4_ atoms only carries a 10% increase of *D*: for *ε*=24.94 kJ mol^−1^ *D* is equal to 1.33±0.13 × 10^−9^ m^2^ s^−1^, whereas *D* increases to 1.47±0.11 × 10^−9^ m^2^ s^−1^ for *ε*=2.49 kJ mol^−1^. Moreover, *D* increases as the surface electrostatic charges decrease. For neutral Fe_3_O_4_ NPs, *D* grows to 1.64±0.04 × 10^−9^ m^2^ s^−1^ and, if both the NPs and nanopore wall are electrically neutral, *D* takes the value of 1.69±0.20 × 10^−9^ m^2^ s^−1^ (~30% increase as compared with the example above). Although the water molecule confinement is affected by the strength of the interaction potentials (van der Waals and Coulomb), geometrical parameters show a greater influence on the coefficient *D*. The reason is that all considered surfaces have effective wall potentials that are strong enough to induce a significant reduction of the water mobility in a region close to the wall. On the other hand, as clarified below, the volume of the low mobility region only slightly depends on the wall potential strength, namely, the minimum of the potential well generated by the wall.

Finally, the self-diffusion coefficient *D* did not change significantly with the level of hydration in the considered range, in accordance with previous studies^7^. Considering again the representative case of eight Fe_3_O_4_ NPs, of 2.0 nm in diameter, adsorbed on the wall of an 8.1-nm SiO_2_ nanopore, *D* ranges from 1.30±0.11 × 10^−9^ to 1.40±0.07 × 10^−9^ m^2^ s^−1^ (<10% variation) as the water density increases from 700 to 930 kg m^−3^.

### Characteristic length of confinement

In the bulk fluid, the water molecules fluctuate with a kinetic energy proportional to *k*_B_*T*, where *k*_B_ is the Boltzmann constant (1.38 × 10^−23^ J K^−1^) and *T* is the temperature. As opposed to molecules in the bulk, those in a close proximity of solid surfaces are subjected to additional van der Waals (*U*_vdw_) and Coulomb (*U*_c_) interactions interfering with their state of agitation. This induces a layering of water molecules with reduced mobility near the solid surface (see Supplementary Figs 1–3 and Supplementary Discussion), as already pointed out in other works^38^,^39^. A characteristic length *δ* can be introduced to quantify the thickness of such confined water layer.

**Figure 2:**
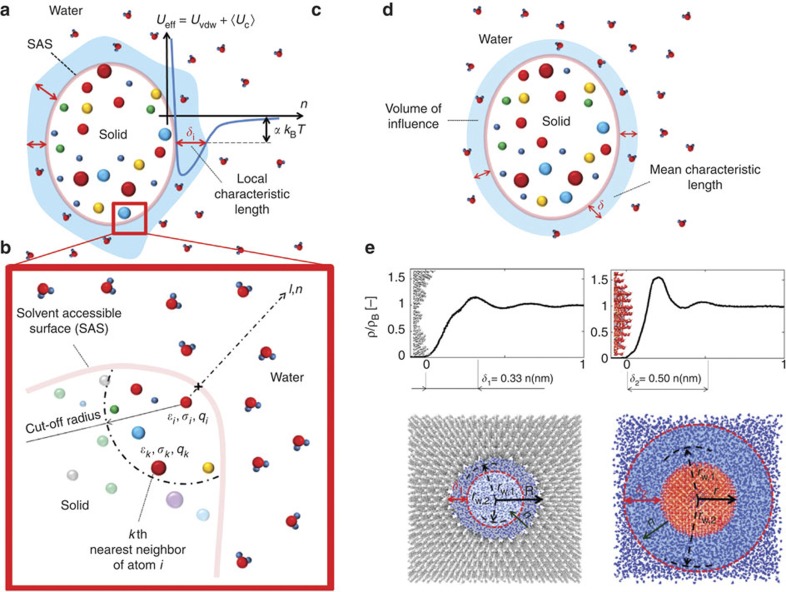
**The characteristic length of confinement** ***δ***. (**a**) A local characteristic length *δ*_*i*_ can be defined at any atom *i* (with non-vanishing SAS). (**b**) The contribution to the total potential energy of neighbours along a direction orthogonal to the SAS (line *l* with local coordinate *n*) is computed. (**c**) The thermal energy level provides a criterion to (locally) define the characteristic length *δ*_*i*_, which typically varies along the whole SAS. (**d**) A characteristic length of confinement *δ* for the whole structure can be defined by a weighted average of all *δ*_*i*_. (**e**) The length of confinement is reported for a SiO_2_ nanopore (*δ*_1_≈0.33 nm) and for a Fe_3_O_4_ NP (*δ*_2_≈0.50 nm). For SiO_2_, only the first water layer (with distance *r*_w,1_ from the pore axis) is located within the volume of influence. For Fe_3_O_4_, both the first and the second water layers (with distances *r*_w,1_ and *r*_w,2_ from the particle centre, respectively) are found within the volume of influence (more cases are reported in the Supplementary Figs 1 and 2).

Referring to the popular notion of solvent accessible surfaces (SAS)^40^,^41^,^42^, the quantities *S*_tot_ and *S*_loc_ can be introduced as the total and specific (per atom) SAS areas, respectively. For an arbitrary atom *i* of the solid structure, a number *N*_*n*_ of nearest neighbours (including the atom *i* itself) can be identified within a fixed cutoff radius (Fig. 2a,b). The corresponding effective potential energy *U*_eff_ on the water molecules, due to both van der Waals (*U*_vdw_) and Coulomb (*U*_c_) interactions, can be computed as:


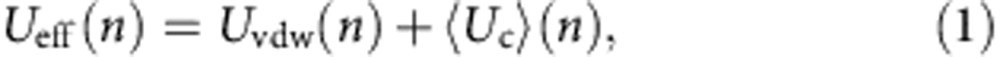


along the *n* direction, orthogonally to the SAS and passing through the centre of the atom *i* (Fig. 2b). For the 12-6 Lennard–Jones potential, it follows that 
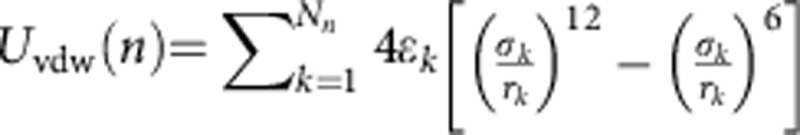
, with *ε*_*k*_, *σ*_*k*_ and *r*_*k*_ denoting the depth of the potential well, the distance at with such potential becomes zero and the Euclidean distance between the generic line point with coordinate *n* and the centre of *k*th nearest neighbour, respectively. For the Coulomb interactions, the average potential energy at a fixed temperature *T* between the *N*_*n*_ atoms and the water dipoles is 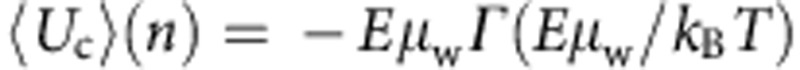, where *E*, *μ*_w_, *k*_B_ and *Γ* denote the electrical field strength, water dipole moment (7.50 × 10^−30^ C m for the SPC/E model), the Boltzmann constant and the Langevin function *Γ*(*x*)=coth(*x*)−1/*x*. The strength of the electrical field *E* can be readily computed following the law of electrostatics (see Methods). Knowing the effective potential *U*_eff_(*n*) for the atom *i*, a corresponding characteristic length *δ*_*i*_ can be estimated within which the water molecules have reduced mobility. This length *δ*_*i*_ is given by *δ*_*i*_=*n*_*i*,2_−*n*_*i*,1_ where *n*_*i*,2_ and *n*_*i*,1_ are the two zeros of the equation *U*_eff_(*n*)+*k*_B_*T*/4=0 (Fig. 2c). Therefore, based on the definition of *δ*_*i*_, all the water molecules located within such a distance are significantly affected by the van der Walls and Coulomb interactions, whereas all the water molecules beyond the characteristic length *δ*_*i*_ can escape the potential well generated by the solid wall. Here *k*_B_*T*/4 is half the kinetic energy per independent degree of freedom according to the equipartition of energy (see Methods). By proper averaging over the surface, the mean characteristic length *δ* of the overall solid surface (Fig. 2d) can be derived as


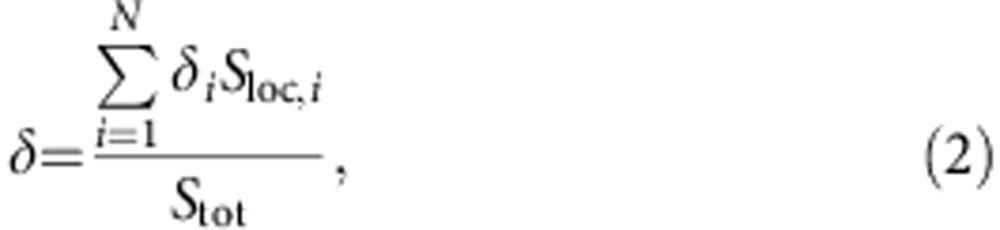


with *S*_loc,*i*_ and *N* being the specific (per atom) SAS for the atom *i* and the total number of atoms, respectively. Note that the above formulation is general and applies to hydrophilic and hydrophobic surfaces, regardless of their electrostatic surface charge. Also, the characteristic length *δ* can be conveniently computed based on the geometry of the problem, Lennard–Jones force field parameters and the partial electrostatic charges using the script provided in the Supplementary Software 1.

In Fig. 2e, the water density profile within (a) a SiO_2_ nanopore and (b) around a single Fe_3_O_4_ NP is shown, where peaks denote the typical water layers nearby a solid wall at the nanoscale^2^,^39^. It is noteworthy that despite the great difference in the potential strength between SiO_2_ and Fe_3_O_4_ (min(*U*_eff,2_)/min(*U*_eff,1_)≈2.7), the corresponding difference in terms of characteristic lengths is much more moderate (*δ*_2_/*δ*_1_≈1.5). From the density profiles, Fe_3_O_4_ clearly induces a stronger perturbation in the nearby water molecules’ distribution. However, except for a first thin water layer strongly adsorbed to the NP surface (and accounted by *δ*_2_>*δ*_1_), the amplitude of these perturbations rapidly decays further away and becomes comparable in the remaining confined volume for both cases. Similarly, although there is a significant difference in the potential minimum between green fluorescence protein and (5,5) chirality CNT (min(*U*_eff,2_)/min(*U*_eff,1_)≈1.5), the difference between the two characteristic lengths is negligible. The above observations suggest that geometrical parameters could more significantly affect *D* as compared to energetic parameters.

### Scaling law

Since water mobility is impaired mostly in a thin layer next to the liquid–solid interface with thickness *δ*, it is reasonable to assume that the observed variation in the self-diffusion coefficient *D* is mainly associated with the altered mobility of the water molecules within such a layer and the corresponding volume (volume of influence— Fig. 2d). In addition, it has been already observed that the self-diffusion coefficient *D* reduces as the ratio between the total interfacial area and the total volume occupied by water increases. On the basis of such an evidence, a scaling parameter *θ* can be introduced as the ratio between the total water volume of influence (*V*_in_) and the total volume accessible to the water molecules (*V*_w_); thus,


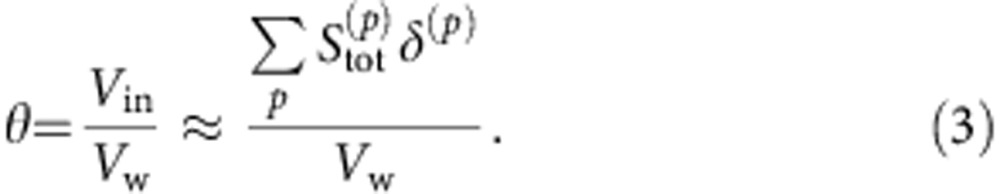


This parameter *θ* varies from 0 (bulk water case) to 1 (totally confined water). The volume of influence (*V*_in_) is the volume of water that feels the van der Walls and Coulomb interactions and is therefore influenced by the presence of solid walls. This volume is readily given by *V*_in_≈∑_*p*_*S*_tot_^(*p*)^*δ*^(*p*)^, where *S*_tot_^(*p*)^ and *δ*^(*p*)^ represent the total SAS and characteristic length of the *p*th particle, respectively. As detailed in the Methods, possible overlap of the volumes of influence due to several particles/pores (for example, several NPs loaded within a SiO_2_ nanopore) can be easily taken into account by the continuum percolation theory (CPT), and a more accurate estimate of *V*_in_ may include the effect of particle curvature.

**Figure 3:**
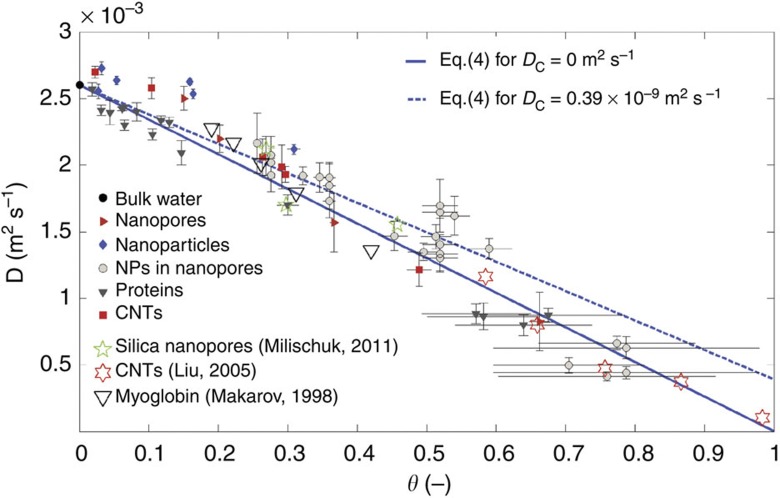
**Scaling behaviour of the water diffusion coefficient** ***D***. The self-diffusion coefficient of water *D* has been calculated for over 60 different cases including spherical NPs in water, water within nanopores with and without spherical NPs, proteins and CNTs in water. Fifty-eight cases are the results of MD simulations performed in this work, under isothermal conditions. Results from the literature are also provided, for which the scaling variable *θ* was computed as from the Supplementary Discussion. The solid and the dashed lines represent equation (4) for *D*_C_=0 and *D*_C_=0.39 × 10^−9^ m^2^ s^−1^, respectively. Equation (4) accurately recovers simulation and literature results with high coefficient of determination (*R*^2^>0.90). The uncertainties on the value of *D* (vertical bars) refer to the fitting of the mean square displacement, whereas the uncertainties on the value of *θ* (horizontal bars) refer to the estimate of the total volume accessible to water molecules *V*_w_ (see Methods).

Assuming *θ* as the sole, independent variable for *D*, all computed values relax within a narrow band around a linear curve (Fig. 3) that can be readily described by the relationship


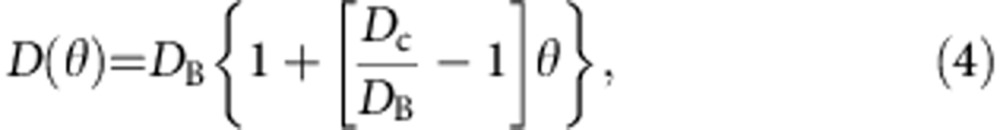


where *D*_B_ is the self-diffusion coefficient of bulk water, while *D*_C_ the self-diffusion coefficient of totally confined water. Remarkably, Fig. 3 presents data from 58 different cases analysed in this work as well as data available in the published literature. Here despite the variety of the considered configurations, particles and sources of the results, a simple law is found to be sufficiently accurate to describe the phenomenon under study, thus confirming that *θ* is indeed an important controlling parameter under very diverse conditions and geometrical configurations. To have a more explicit formulation of equation (4), details about the evaluation of *D*_C_ are provided in the section below.

### Thermodynamic insights

**Figure 4:**
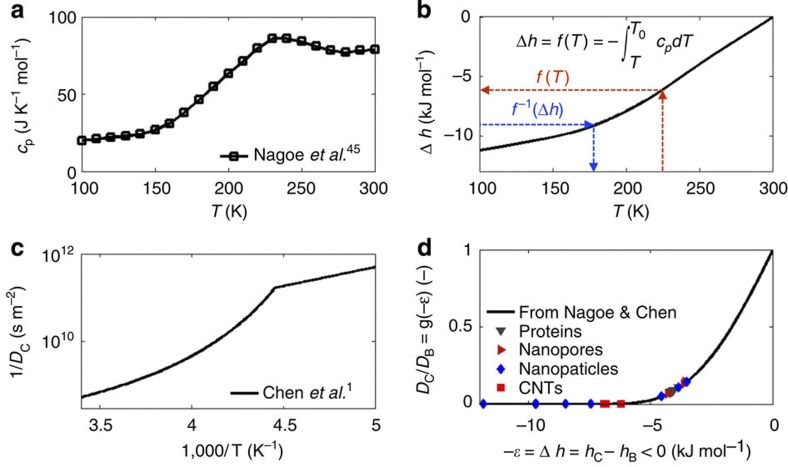
**Derivation of the function** ***D***_**C**_/***D***_**B**_=***g***(**−*ε***). (**a**) The specific heat capacity *c*_*p*_(*T*) of water confined in the narrowest SiO_2_ pore reported in Nagoe *et al*.^45^ (**b**) By integrating the latter function *c*_*p*_(*T*), the energy of nanoconfinement is evaluated as 
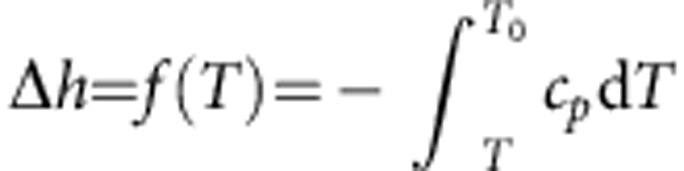
. (**c**) The inverse function *T*=*f*^−1^(Δ*h*) provides the temperature corresponding to a given energy Δ*h;* hence, it can be used in combination with the experimental results from Chen *et al.*^1^ where the diffusion coefficient (in narrow SiO_2_ pores) is given as a function of *T*. (**d**) The ratio between the diffusion *D*_c_ of fully confined water and the bulk diffusion *D*_b_ of water, *D*_C_/*D*_B_=*g*(Δ*h*), is reported and the corresponding values for all the considered configurations are shown, where *g*(Δ*h*)<0.15.

Nanoconfined water shares some features with supercooled ordinary water in that it may not crystallize on cooling below the melting temperature of *T*_M_≈273.15 K (refs 1, 43). Within the thin *δ* layer of water molecules next to a solid surface, the thermodynamic state depends on the characteristic confinement length scale^44^. In particular, the specific heat capacity *c*_p_ of nanoconfined water has been experimentally measured in narrow SiO_2_ nanopores and its variation with the temperature *T* is plotted in Fig. 4a for a pore diameter of 1.7 nm (ref. 45). Using these experimental data, the energy variation associated with the transition from bulk to confined water can be readily computed as 
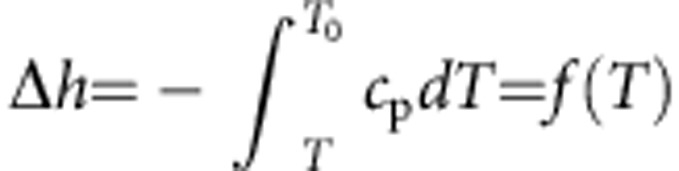
, with *f*(*T*_0_)=0 at the bulk water temperature *T*_0_=300 K. From this, the inverse function 
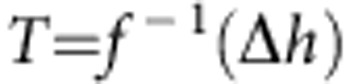
 can be derived as plotted in Fig. 4b. The energy variation associated with water nanoconfinement can also be written as 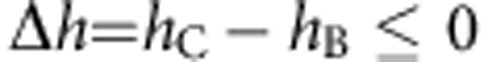, where *h*_B_ and *h*_C_ are the energy of bulk and confined water, respectively. Since the confined region is typically limited to 1–2 layers of water molecules (for all the considered structures, *δ*<0.6 nm), it can be assumed that *h*≈−*ε*, which is the minimum of the effective potential (*U*_eff_) generated by the solid surface. The function *f*^−1^ can be used to compute the supercooled temperature of nanoconfined water corresponding to the energy variation −*ε*, that is, to say *T*=*f*^−1^(−*ε*). In the work by Chen *et al.*^1^, the diffusion coefficient of water molecules under strong nanoconfinement (that is, within SiO_2_ pores with diameter of 1.4–1.8 nm) is reported as a function of the temperature. From the latter experimental data, the diffusion coefficient of totally confined water *D*_C_ can be readily expressed in the form *D*_C_=*D*_B_ *g*(*T*) (Fig. 4c). Therefore, by combining the two sets of experimental data and knowing the value of *ε*, the diffusion coefficient of totally confined water *D*_C_ can be computed as *D*_C_/*D*_B_=*g*′(*T*)=*g*′(*f*^−1^(−*ε*))=*g*(−*ε*). The ratio *D*_C_/*D*_B_ is plotted in Fig. 4d for the different 58 cases analysed here. For the iron oxide NPs and CNTs, which are characterized by a fairly strong effective potential (large *ε*), *D*_C_≈0. As the strength of the effective potential reduces, the value for the diffusion coefficient of totally confined water increases. However, even for the SiO_2_ NPs and nanopores, and for the proteins that are characterized by lower *ε*, in our computations *D*_C_ is at most 15% of *D*_B_. We stress that the function *g*(Δ*h*) is a property of water, and here we have chosen to estimate it on the basis of measurements in SiO_2_ nanopores only, because such experiments are among the very few that are well documented in literature.

In general, the volume of water can be partitioned into bulk (B) and confined (C). Invoking the mixing rule, the average diffusivity *D* of the system can be presented as


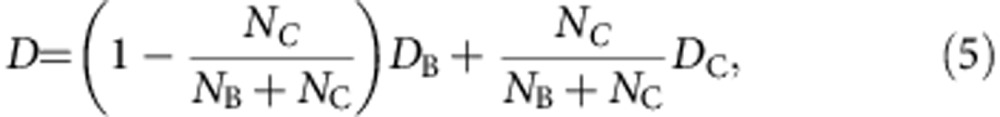


where *N*_C_ and *N*_B_ are the number of water molecules in the confined and bulk regions, respectively. Considering that *N*_C_=*ρ*_C_*V*_C_/*m*_w_, with *ρ*_C_ being the water (mean) density in the adsorbed region and *m*_w_ being the mass of one water molecule, it follows:


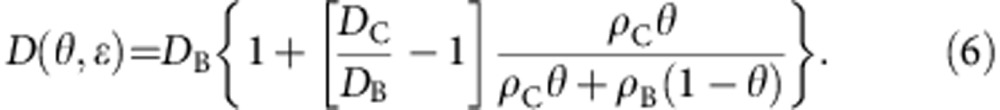


This implies that the average diffusivity *D* depends in general on a geometrical parameter (*θ*) and an energetic parameter (*ε*). However, the following approximation (*ρ*_C_*θ*)/(*ρ*_C_*θ*+*ρ*_B_(1−*θ*))≈*θ* can be safely made for the full range of *θ* (see Supplementary Fig. 4 and Supplementary Discussion). Therefore, equation (6) degenerates into equation (4) demonstrating that the diffusion of nanoconfined water can be interpreted invoking the thermodynamics of supercooled water. Moreover, based on the above discussion on *D*_C_ (see Fig. 4d), for a large variety of nanoconfined systems, the simplifying assumption *D*_C_/*D*_B_≈0 can be safely made. In such cases, the much simpler law *D*(*θ*,*ε*)≈*D*(*θ*)=*D*_B_(1−*θ*) is readily derived showing the direct linear dependence of the water diffusion coefficient on the sole scaling parameter *θ*. Note that no empirical factor is needed to derive *D*(*θ*), with the latter law matching the values of the 58 MD simulations as well as 13 further configurations from the literature with a quite good coefficient of determination (*R*^2^=0.93, solid line in Fig. 3). The dashed line in Fig. 3 represents, instead, equation (4) with *D*_C_/*D*_B_=0.15, corresponding to the largest value of *D*_C_ only observed in a few simulated cases (Fig. 4d).

## Discussion

**Figure 5:**
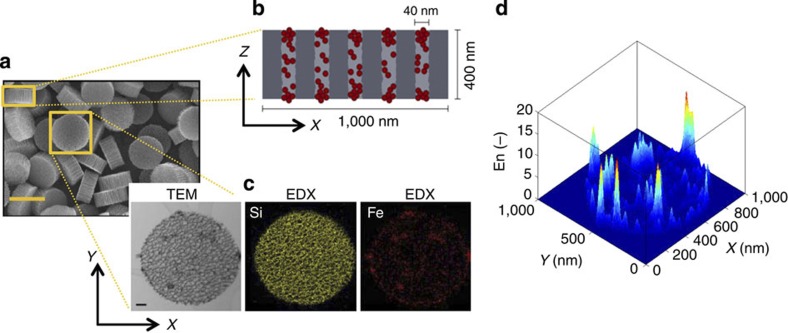
Relaxivity of SPIO-loaded mesoporous silicon nanoconstructs. (**a**) Scanning electron microscopy image of discoidal mesoporous silicon nanoconstructs (with cross section area 1000 × 400 nm^2^) loaded with 5 nm SPIOs. Scale bar, 1 μm. (**b**) Schematic representation of the pores within the silicon nanoconstructs loaded with the SPIOs (red circles). (**c**) TEM and EDX images showing the distribution of SPIO clusters within the silicon nanoconstructs. The black dots in the TEM image and red dots in the EDX image identify the SPIOs; the yellow lines in the EDX image represent the Si walls of the mesoporous silicon nanoconstructs. Scale bar, 100 nm. (**d**) Diagram showing the local enhancement in transversal magnetic resonance relaxivity as calculated via the *D*(*θ*) relationship.

It has been shown that the relaxometric properties of contrast agents for MR imaging can be enhanced by proper modulation of the water molecule dynamics^46^. Recently, Decuzzi and collaborators^23^,^24^ have shown that the geometrical confinement of Gd^3+^-based MRI contrast agents and SPIOs within mesoporous structures can increase by several folds the longitudinal and transversal relaxivities, respectively, of the original agents. Here it is shown that the scaling law *D*(*θ*) can be effectively used to predict the enhancement in transversal relaxivity exhibited by SPIOs confined in mesoporous silicon structures. Referring to Fig. 5a, discoidal mesoporous silicon particles (SiMPs) of 1,000 nm in diameter and with an average pore size of 40 nm are synthesized by a combination of optical lithographic techniques and electrochemical etching^26^. These particles are exposed to a concentrated solution of SPIOs, which are loaded within the pores by capillary suction and form clusters of controlled size. As schematically shown in Fig. 5b and documented by the transmission electron microscopy (TEM) and energy-dispersive X-ray spectroscopy (EDX) images of Fig. 5c, the SPIOs are distributed more or less uniformly within the pores and across the porous matrix of the silicon particles. The black and red spots, respectively, in the TEM and EDX images identify the SPIO clusters within the mesoporous silicon matrix. In these experiments, commercially available SPIOs (Sigma-Aldrich) are considered, coated with a thin layer of polyethylene glycol to facilitate their dispersion in water, and presenting an average core diameter of 5.13±1.07 nm, as derived by TEM analysis. In bulk water the transversal relaxivity of the unconfined SPIOs has been measured to be (*r*_*2*_=107±24 mM^−1^ s^−1^), whereas on geometrical confinement within the SiMPs, the relaxivity rises up to *r*_*2*_=270±73 mM^−1^ s^−1^.

From the theory on the MR relaxation of iron oxide NPs, the transversal relaxivity *r*_2_ of the SPIOs dispersed in an aqueous solution can be estimated as^29^,^32^,^47^


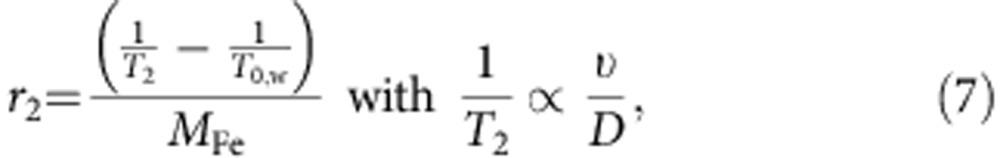


where *T*_2_ and *T*_0,w_ are the transversal relaxation times for the solution with contrast agents and bulk water (=2,800 ms), respectively; *M*_Fe_ is the iron concentration (mM) in the solution; *υ* is the volume fraction of the SPIOs; and *D* is the diffusion of the water molecules surrounding the SPIOs. Details on equation (7) are provided in the Supplementary Discussion.

For a fixed iron concentration *M*_Fe_ in the solution, the enhancement in transversal relaxivity is directly related to the ratio *En*=(*υ*/*D*)/(*υ*_B_/*D*_B_), where the quantity *υ*/*D* has to be estimated for confined SPIOs and *υ*_B_/*D*_B_ for the free SPIOs dispersed in bulk water. Since the distribution of the SPIOs within the SiMP is not uniform, the diffusion coefficient *D* and the local SPIO volume fraction *υ* are expected to vary within the porous matrix. The local enhancement in relaxivity *En* is plotted in Fig. 5d. Areas are shown with mild and high enhancement, up to 20-fold, corresponding to different levels of SPIO loading and clustering, and therefore different levels of water confinement. By integrating over the whole silicon particle, an average relaxivity enhancement 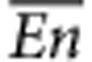 can be computed as being ~2.7, which is in excellent agreement with the experimental value of ~2.52 (see Methods for details). Note that the proposed law can be used in several other fields to analyse different cases of water confinement.

In summary, it has been shown that the self-diffusion coefficient of nanoconfined water can be described by a unique dimensionless parameter *θ*, representing the ratio between the confined and total water volumes. The coefficient *D* scales linearly with *θ* and can be readily estimated, knowing the bulk *D*_B_ and totally confined *D*_C_ diffusion of water. This has been validated on the basis of almost 60 different cases and 5 different geometrical configurations, including the analysis of the water molecule dynamics within nanopores and CNTs, around NPs and proteins. The coefficient of diffusion for confined *D*_C_ water is quantified on the basis of the thermodynamics of supercooled water. As an example, the scaling relation has been shown to accurately predict the enhancement in magnetic resonance relaxivity of iron oxide NPs confined into mesoporous silicon structures.

The proposed approach may be used to interpret experimental data collected in different scientific disciplines on the dynamics of water molecules under confined conditions and to rationally design nanostructures for modulating the diffusion of water. This is of relevance in nanomedicine, nanotechnology as well as in more traditional engineering fields such as heat transport, fluid dynamics and energy storage.

## Methods

### MD simulations

Atomic coordinates of Fe_3_O_4_ NPs are generated from Fe_3_O_4_ crystals^48^, whereas SiO_2_ crystals are considered in the case of nanopores and SiO_2_ NPs (Supplementary Figs 5–8)^49^. Crystal structures of proteins are taken from the Protein Data Bank ( http://www.rcsb.org; Supplementary Figs 9 and 10). CNTs are generated by means of the Visual Molecular Dynamics software (Supplementary Fig. 11)^50^. The SPC/E model^51^ is used for water molecules, which is known to accurately predict some of the properties of water relevant for this study at room temperature^37^. However, it is also worth noticing that the SPC/E model does not accurately predict some other properties of water. For instance, shear viscosity or thermal conductivity were found to be off by more than 50% at room temperature^52^. Bonded interactions of SiO_2_ and Fe_3_O_4_ are treated by means of harmonic stretching and angle potentials^53^. Van der Waals interactions are modelled by a 12-6 Lennard–Jones potential; partial charge interactions between solid surfaces and water are modelled by a Coulomb potential (Supplementary Tables 12–14)^51^,^54^,^55^,^56^. Non-zero partial charges are only assigned to atoms on the surface of nanopore or NP, which belong to silanol and FeOH groups, whereas all other atoms (bulk of SiO_2_ and Fe_3_O_4_) are considered as neutral.

Simulations are carried out with a leap-frog algorithm (time step: Δ*t*=0.5 fs), and periodic boundary conditions are applied along the three Cartesian coordinates. After energy minimization of NP, nanopore or nanotube setups, the two subsystems (solid crystals and water) are initialized at 300 K (Maxwellian distribution of velocities) and fully coupled to a Nosé–Hoover thermostat^57^,^58^ (at 300 K and time constant *τ*=0.2 ps) for 50 ps, until the energies of the system relax to a steady state. During the latter preliminary calculation, one thermostat for each subsystem is adopted. Afterwards, Nosé–Hoover thermostats (at 300 K) are maintained attached to solid crystals only, whereas the simulation is continued up to 2 ns. In the case of proteins, the MD protocol is slightly changed to improve convergence. Energy minimization for proteins is performed before and after solvation and ions are added when needed for achieving the neutrality of the system, which is then equilibrated in two steps: 100 ps in canonical ensemble (fixed number N of particles, volume V and temperature T - NVT) at 300 K (initialization with Maxwellian distribution of velocities, Berendsen thermostats^59^ separately attached to proteins and water, *τ*=0.1 ps); 100 ps in NPT ensemble (fixed number N of particles, pressure P and temperature T) at 300 K and 1 bar (Berendsen thermostats separately attached to proteins and water, *τ*=0.1 ps; Parrinello–Rahman pressostat^60^ applied to the whole system, *τ*=2 ps). During the equilibration, all bonds in the proteins are kept rigid using the LINCS (Linear Constraint Solver) algorithm^61^. Finally, a Nosé–Hoover thermostat (300 K, *τ*=0.2 ps) is attached to protein’s atoms and the simulation is continued for 1 ns.

In all simulated cases, steady state is reached when *D*, which is evaluated every 200 ps, tends to an asymptotic value (Supplementary Figs 12–28 and Supplementary Note 2). This is generally achieved after about 600 ps, for all configurations. Note that the root mean square deviation of the proteins in the water box with respect to the crystallographic structures is on average found to be below 0.3 nm (Supplementary Fig. 29 and Supplementary Note 2). The self-diffusion coefficient *D* of the water molecules is determined following the classical relationship of Einstein and computing the mean square displacement as^5^,^62^ 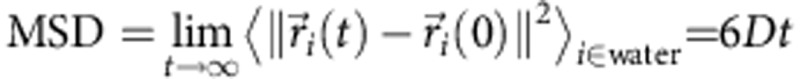, where the position vector 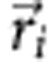 refers to the centre of mass of the water molecule *i* at the generic time *t* and 0 refers to the initial configuration of the system. Alternative approaches for computing the water diffusivity could be considered as well (for example, those based on the first-passage concept)^63^,^64^. Further details on the implementation of the MD simulations and calculation of the self-diffusion coefficient *D* are provided in the Supplementary Methods.

The MD simulations are performed with the software package GROMACS^65^,^66^. Rendering is performed with UCSF Chimera^67^.

### Derivation of the characteristic length *δ*

When defining the van der Waals potential *U*_vdw_(*n*) in equation (1), the parameters *ε*_*k*_ and *σ*_*k*_ already incorporate a combination rule for the Lennard–Jones parameters between the atom *i* and oxygen atoms of water (for example, the Lorentz–Berthelot rule). Moreover, in the average potential 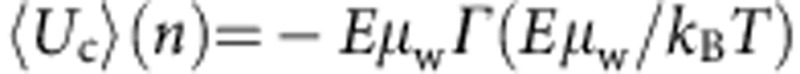, the effective strength of the electrical field *E*=|***E***(*n*)| may be expressed by the following explicit form:


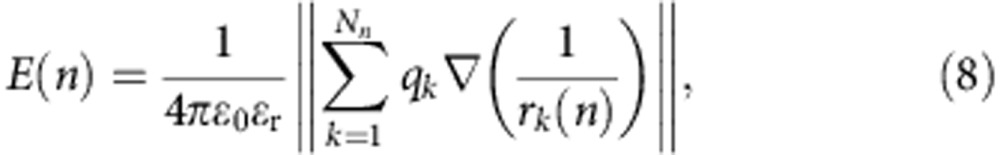



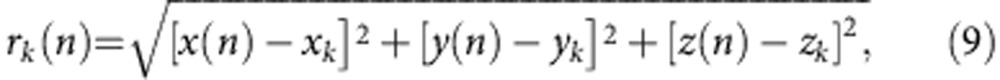


with *q*_*k*_ being the electric charge of the *k*th neighbour, while (*x*,*y*,*z*) and (*x*_*k*_,*y*_*k*_,*z*_*k*_) represent the Cartesian coordinates of the generic point on *l* (corresponding to the local coordinate *n*) and the Cartesian coordinates of neighbour *k*, respectively. In our approach, the relative permittivity *ε*_r_ is an input parameter to be provided to the Matlab(r) routine for the computation of *δ* (see below, Supplementary Fig. 30, Supplementary Methods and Supplementary Software 1). In particular, in this work, *ε*_r_ was included as a function of the distance from the particle *ε*_r_(*n*) following the suggestion in ref. 68.

The expression of 
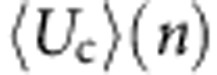
 is a classical result of electrostatics and can be justified as follows. Let *E* be the electric field acting on a dipole **μ**. The instantaneous energy can be expressed as 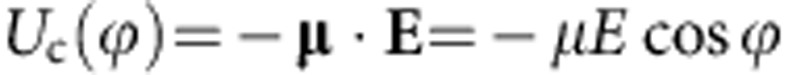, with *E* and *μ* being the field and dipole strength, respectively, while *ϕ* is the angle between the direction of the dipole and the field. The minimum and maximum values of energy are attained for *ϕ*=0 and *π*, respectively. However, in the presence of thermal agitation, the direction of **μ** (hence *ϕ*) is continuously changing in time. For classical systems, a number of independent dipoles are distributed according to their energy level *U*_c_:*U*_c,min_<*U*_c_<*U*_c,max_. In particular, at thermodynamic equilibrium, the Boltzmann distribution predicts that the number *N*(*U*_c_) of dipoles with energy *U*_c_ is: 
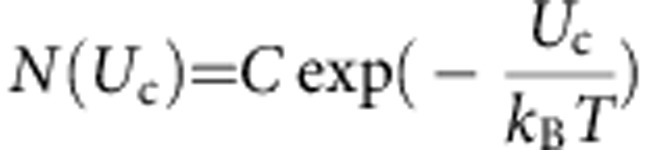
, with *C* being a constant to be determined. Note that, in three dimensions, all dipoles lying on a cone with angle 2*ϕ* around the electrical field direction have the same energy. Moreover, at every *ϕ*, the total number of such dipoles is *N*(*U*_c_(*ϕ*))dΩ, with Ω being the incremental solid angle. The above dipoles at *ϕ* give the component sum d*μ* in the field direction: 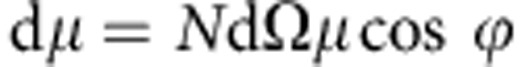. Hence, according to the Boltzmann distribution, the average dipole moment takes the more explicit form:


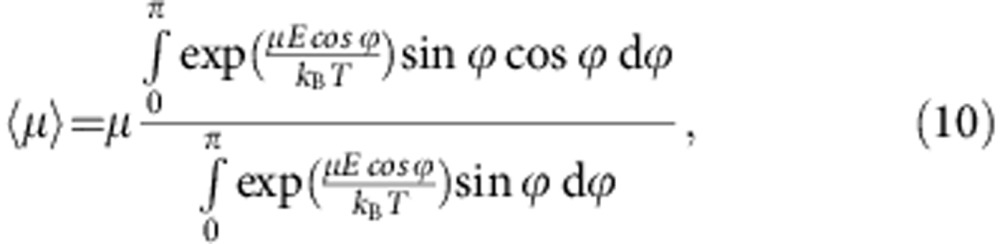


On substituting *β*=*μE*/*k*_B_*T* and 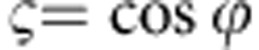, the above integral becomes 
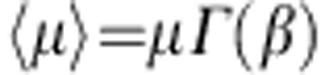
.

On construction of the potential in equation (1) at an arbitrary atom *i*, a local characteristic length *δ*_*i*_ can be defined as *δ*_*i*_=*n*_*i*,2_−*n*_*i*,1_, where *n*_*i*,2_ and *n*_*i*,1_ are the two zeros of the equation (see Fig. 2c): *U*_eff_(*n*)+*αk*_B_*T*=0, where we expect *α*≈1/4. In fact, provided that *k*_B_*T*/2 is the kinetic energy attributed the each degree of freedom of the water molecules, for planar surfaces *α*=1/4 because molecule are allowed to escape the potential well only along half of the direction orthogonal to the surface. The previous equation implies that water molecules located beyond the characteristic length *δ*_*i*_ are in the position of escaping the potential well generated by the *N*_*n*_ atoms in the solid wall owing to the kinetic energy *k*_B_*T*. Obviously, when the horizontal line *U*+*αk*_B_*T*=0 does not intersect the function in equation (1), *δ*_*i*_=0.

In general, the quantity *δ*_*i*_ varies at each atom *i* (see Fig. 2a). Moreover, (meaningless) non-zero values for *δ*_*i*_ can be found for bulk atoms. Thus, for a given solid structure, it is convenient to define a mean characteristic length as in equation (2) (see Fig. 2d). Note that both *S*_tot_ and *S*_loc,*i*_ are readily computed by GROMACS, once the geometry of the system is known (for example, in the form of a pdb file). It is also worth emphasizing that *δ* is a characteristic length of the whole system of interest and can be straightforwardly computed based on the geometry, Lennard–Jones force field parameters and partial charges.

The script for computing *δ* is based on Matlab(r) and it is provided as Supplementary Software 1 of this work.

### Derivation of the scaling parameter *θ*

Finding a proper scaling parameter *θ* (or equivalently *δ*) is not trivial. A few unsuccessful attempts to find a general scaling parameter for water self-diffusion coefficient are reported in Supplementary Discussion and Supplementary Figs 31 and 32.

In equation (3) the particle curvature is neglected; thus, this is accurate in the limit 
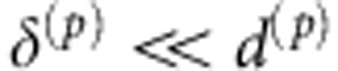
, with *d*^(*p*)^ being a representative radius of curvature of the *p*th particle. If the latter approximation is not properly fulfilled, a more accurate estimate of the numerator at the right-hand side of equation (3) can be easily adopted (see also a few examples in the Supplementary Discussion).

Clearly, in cases of strong confinement (and high values of *θ*) with the presence of several particles (for example, the reported studies where a number of spherical NPs are loaded within cylindrical nanopores), a partial overlap of the volumes of influence becomes more and more probable. However, we notice that volumes of influence due to different particles are not additive. As a result, the quantity *θ* computed by equation (3) is only apparent, with the effective fraction of the volume of influence being smaller than *θ*. The above issue is encountered in the framework of CPT. To this respect, a classical result of CPT, under the assumption of randomly placed volumes suggests that, to properly recover the effective fraction, the apparent volume fraction should be corrected as: 1−exp (−*θ*)^69^,^70^. Hence, we suggest that the above correction applies in the presence of overlap of the volumes of influence (for example, several particles within the computational box).

### The volume *V*_w_

For fully hydrated simple geometries, *V*_w_ can be computed by considering the nominal size of a particle/pore (see the discussion below about relaxivity enhancement). However, in general, the volume *V*_w_ in equation (3) can be defined as:


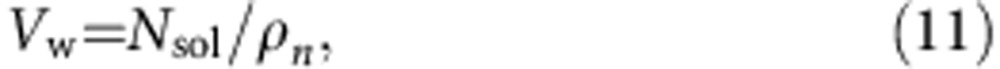


where *N*_sol_ and *ρ*_*n*_ are the number of water molecules within the computational periodic box and the average water density, respectively. As a consequence, the volume occupied by a solvated particle *p* is *V*_*p*_=*V*_box_−*V*_w_, where *V*_box_ is the volume of the computational box. For complex configurations (for example, several NPs surrounded by water or within nanopores), the volume *V*_w_ can be estimated as 
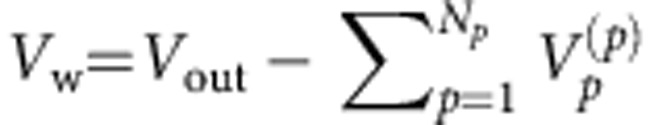
, where *V*_*p*_^(*p*)^ and *N*_*p*_ are the volume of the *p*th particle and the total number of particles, respectively, whereas *V*_out_ is the volume of the surrounding space (that is, *V*_out_=*V*_box_ for particles not loaded in nanopores, while *V*_out_=*V*_pore_ for cases where particles are loaded within a pore whose volume, computed by equation (11), is *V*_pore_).

Clearly, the use of equation (11) relies on the computation of the number density *ρ*_*n*_. Using available packages in standard MD software, an estimate of the average value for *ρ*_*n*_ can be easily computed after solvation of a dry geometry. In our computations, we estimated the water volume in a few realizations of each configuration of interest, several measures of *ρ*_*n*_ were collected and used to compute an average value and the corresponding s.d. The latter s.d. generates uncertainties when computing the volume *V*_w_, and consequently uncertainties of the scaling parameter (horizontal error bars in Fig. 3). Alternative approaches based, for instance, on Monte–Carlo integration can also be used to calculate *V*_w_ accurately.

### Characterization of nanoconstructs and NPs

Details on the fabrication and characterization of the discoidal SiMPs and the SPIOs are provided in the Supplementary Methods and Supplementary Figs 33 and 34, together with the protocols for the loading of SPIOs into SiMPs.

### Relaxivity measurements

*In vitro* relaxation times were measured in a Bruker Minispec (mq 60) bench-top relaxometer operating at 60 MHz and 37 °C. The transverse (*T*_2_) relaxation times were measured using the Carr–Purcell–Meiboom–Gill sequence.

### Analysis of relaxivity enhancement

The function *f*_EDX_(*X*,*Y*) of the two spatial coordinates *X* and *Y* is built from the EDX image for iron (Fig. 5c) and denotes the position of the centre of a generic image pixel. Let *N*_SPIO_ be the average number of SPIOs within a single SiMP. Hence, a surface density function at a pixel scale for the SPIOs can be written as


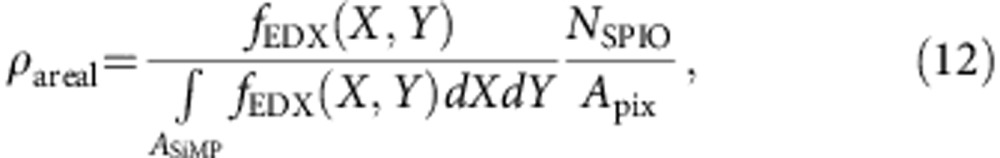


where *A*_pix_ and *A*_SiMP_ are the surface area of a single pixel and the surface area of a SiMP, respectively. The function *ρ*_areal_ has been reported in the (left) bottom part of Supplementary Fig. 35. SiMP pores that are loaded by SPIOs can be automatically recognized by finding the local density map of the EDX non-zero elements (*ρ*_point_). The density function in equation (12) depends on the image pixel size (that is, a quantity that is not representative of the phenomenon under study), whereas a more meaningful function should be based on the pore characteristic dimension instead (representative of the confinement length). Hence, the following surface density function at a pore scale for the SPIOs is introduced: *ρ*_SPIO_(*X*,*Y*)=*Cρ*_point_(*X*,*Y*), with *C* being a constant to be determined. To this end, owing to mass conservation, it is imposed


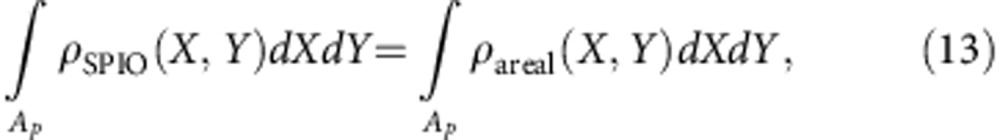


hence,


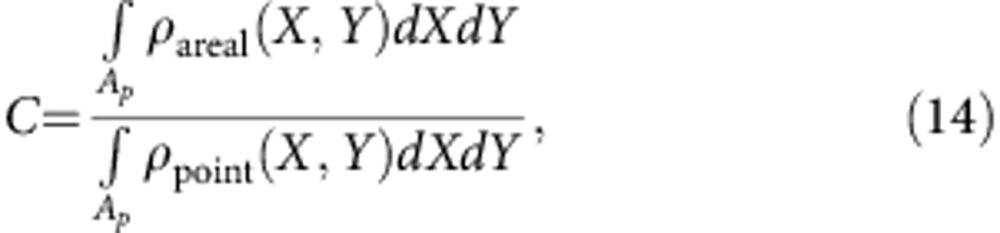


with *A*_*p*_ being the surface area of a representative SiMP pore (see also the (right) bottom part of Supplementary Fig. 35). The total number of SPIOs within the representative pore can be computed as 
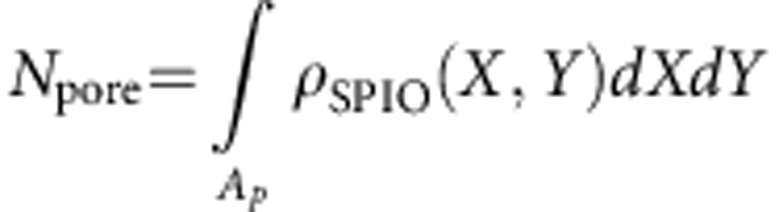
. Hence, the water volume near the SPIOs (for the latter pore) *V*_w_ can be estimated as 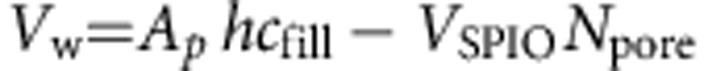, where *h* is the SiMP height, while 0≤ *c*_fill_≤1 indicates the fraction of the SiMP pore that has been effectively occupied by SPIOs. Clearly, for homogeneously distributed SPIOs within the pore, *c*_fill_=1. For the considered representative pore, an estimate of the average scaling parameter 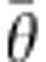 can be obtained as: 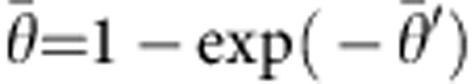
with


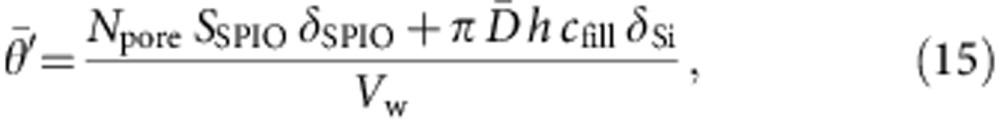


where 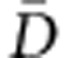 is the average diameter of the pores. Finally, a map of the scaling parameter *θ*=*θ*(*X*,*Y*) is derived by the map *ρ*_SPIO_ as *θ*(*X*,*Y*)=*C*′*ρ*_SPIO_(*X*,*Y*), where the constant *C*′ is determined by imposing


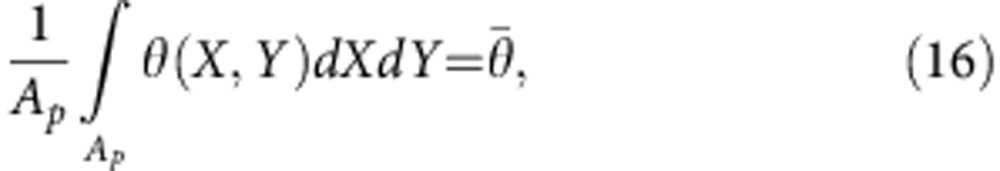


hence


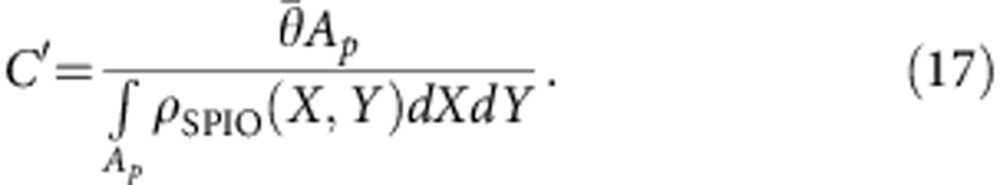


On the estimate of the scaling parameter map *θ*=*θ*(*X*,*Y*), the computation of a corresponding map for the diffusion coefficient *D*=*D*(*X*,*Y*) is straightforwardly achieved by adopting the law suggested in this work.

The above procedure is slightly sensitive to the choice of the representative pore. However, from our computations no significant changes in the final result (map of *θ*=*θ*(*X*,*Y*)) was experienced by making difference choices. In Supplementary Fig. 36, we report both the map of the scaling parameter *θ*=*θ*(*X*,*Y*) and the corresponding *D*=*D*(*X*,*Y*) at different pore filling.

Let us consider two contrast agents with the same iron molarity *M*_Fe_. Let the first one be based on SPIOs homogenously dispersed in bulk water, while the second one adopts the same SPIOs (nanoconfined) within SiMPs. Given the definition of *r*_2_ (Supplementary equation (9)), we can define a relaxivity enhancement as follows:


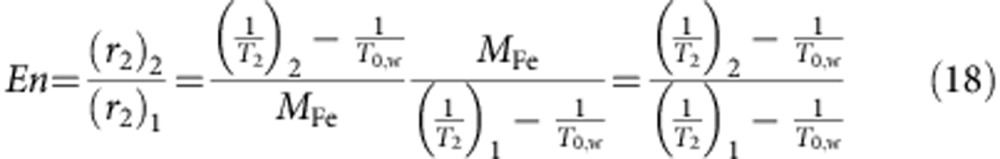


Since 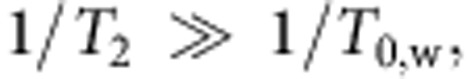



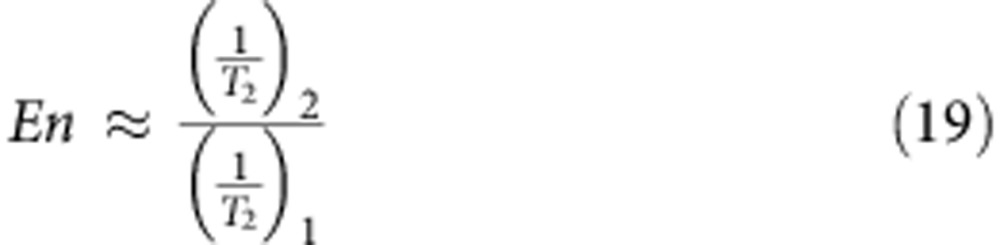


Finally, using the outer sphere theory (see Supplementary Discussion), the enhancement can be recast as


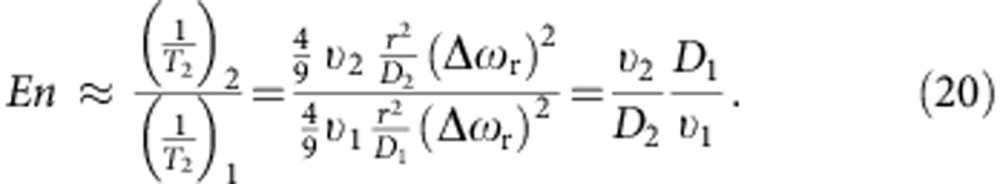


Consistently with the above procedure, we aim at estimating the map *En*(*X*,*Y*):


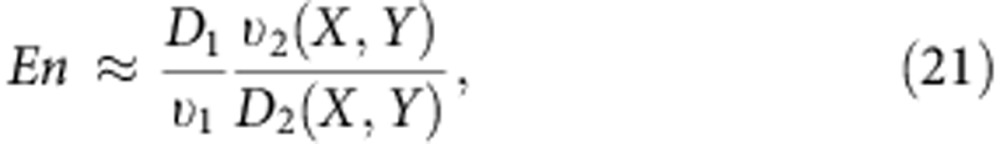


where *D*_1_ and *υ*_1_ are the self-diffusion coefficient of bulk water and the average volume fraction of SPIOs, respectively. Let us assume that 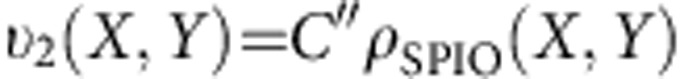 with 
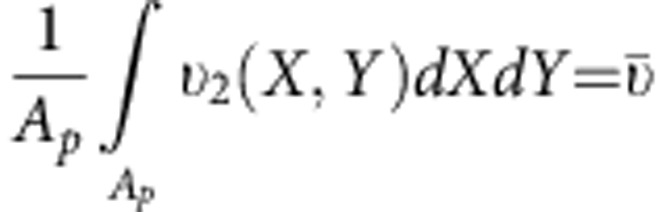
; hence,


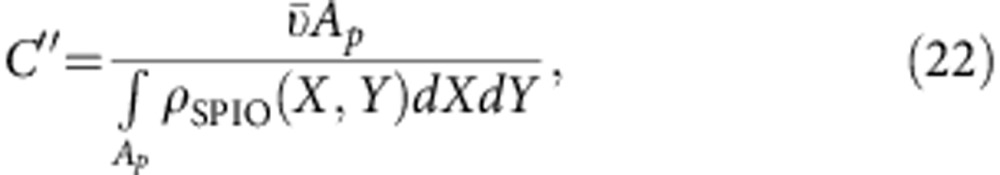


and


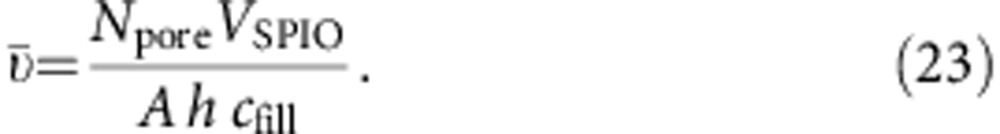


In Fig. 5d we report the function *En*(*X*,*Y*) corresponding to the SiMP particle of Fig. 5c.

Finally, the average enhancement due to the entire SiMP can be estimated as


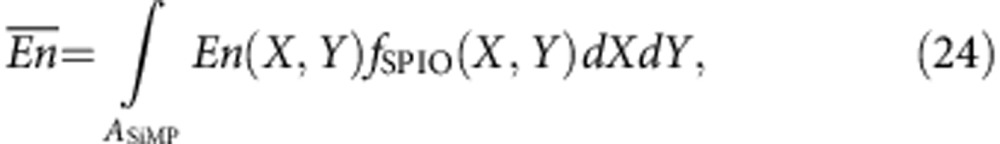


where *f*_SPIO_(*X*,*Y*) is the following distribution function


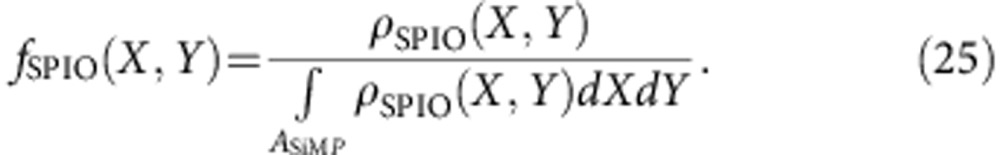


For the case in Fig. 5d, we find 
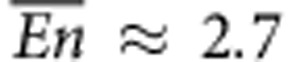
. In Fig. 5c, we consider SiMPs filled by 5 nm SPIOs, the transverse relaxivity of which was experimentally assessed as (*r*_2_)_2_=270±73 mM^−1^ s^−1^. Moreover, 5 nm SPIOs (Sigma-Aldrich) show transverse relaxivity in bulk conditions of water (*r*_2_)_1_=107±24 mM^−1^ s^−1^. As a result, the experimentally measured enhancement for the agent in Fig. 5c (compared with SPIOs in bulk water) is 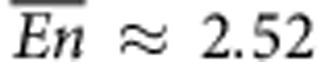, which is in excellent agreement with the above prediction, fully based on the EDX signal (iron) and our model.

## Author contributions

E.C. and M.F. designed the computational plans. M.F. performed all the MD simulations. P.A. and E.C. conceived the idea of the suggested scaling. P.D. conceived the idea of the study, and with P.A. designed and coordinated the project. All the authors interpreted and discussed the computational and experimental results and wrote the manuscript.

## Additional information

**How to cite this article:** Chiavazzo, E. *et al.* Scaling behaviour for the water transport in nanoconfined geometries. *Nat. Commun.* 5:3565 doi: 10.1038/ncomms4565 (2014).

## Supplementary Material

Supplementary Figures, Tables, Notes, Discussion, Methods and References Supplementary Figures 1-36, Supplementary Tables 1-14, Supplementary Notes 1-2, Supplementary Discussion, Supplementary Methods and Supplementary References

Supplementary Software 1 Matlab(r) routine for computing characteristic length of the confinement
