# Supplementary material for: Scaling behaviour for the water transport in nanoconfined geometries
Source: Nat Commun. 2014 Apr 3;5:3565. doi: 10.1038/ncomms4565 (PMC3988813; doi:10.1038/ncomms4565)
Supplement: Supplementary Figures, Tables, Notes, Discussion, Methods and References — Supplementary Figures 1-36, Supplementary Tables 1-14, Supplementary Notes 1-2, Supplementary Discussion, Supplementary Methods and Supplementary References [file ncomms4565-s1.pdf]

SUPPLEMENTARY FIGURES

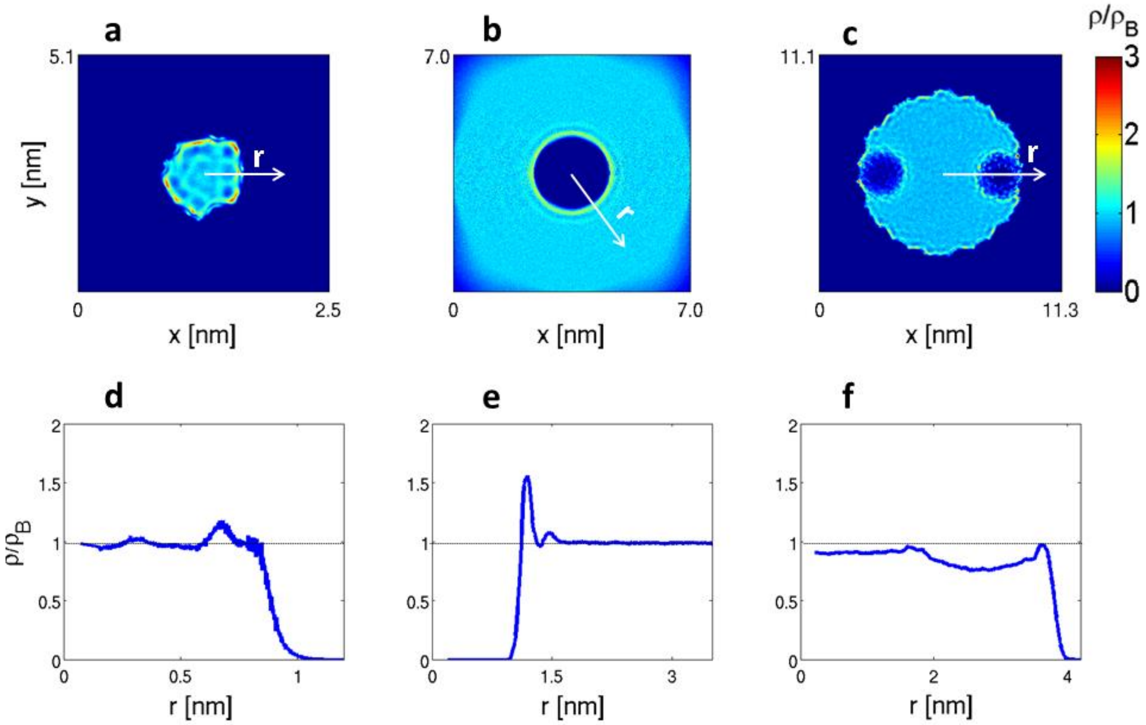

Supplementary Figure 1. Water density ρ within nanopores or around NPs. All density values are normalized by the bulk density $\rho_B = 1000$ kg m^{-3} at 300 K. **(a)** 2D distribution of ρ/ρ_B within a silica nanopore (Φ =2.0 nm), which is calculated by averaging ρ along the nanopore axis. **(b)** 2D distribution of ρ/ρ_B around a magnetite NP ($\phi =$ 2.0 nm) within a cubic water box (L=7.0 nm), which is calculated by averaging ρ along the radius of NP. **(c)** 2D distribution of ρ/ρ_B within a silica nanopore ($\Phi = 8.1$ nm) filled by 4 magnetite NPs ($\phi = 2.0$ nm), which is again calculated by averaging ρ along the nanopore axis. Only 2 out of 4 NPs are visible, because they are placed on the same x, y coordinates along the pore axis. **(d)** Radial ρ/ρ_B within a silica nanopore (Φ =2.0 nm), where r=0 nm lies on nanopore axis. **(e)** Radial ρ/ρ_B around a magnetite NP ($\phi = 2.0$ nm), where r=0 nm lies on NP barycenter. **(f)** Radial ρ/ρ_B within a silica nanopore (Φ =8.1 nm) filled by 8 magnetite NPs ($\phi =$ 2.0 nm), where r=0 nm lies on nanopore axis.

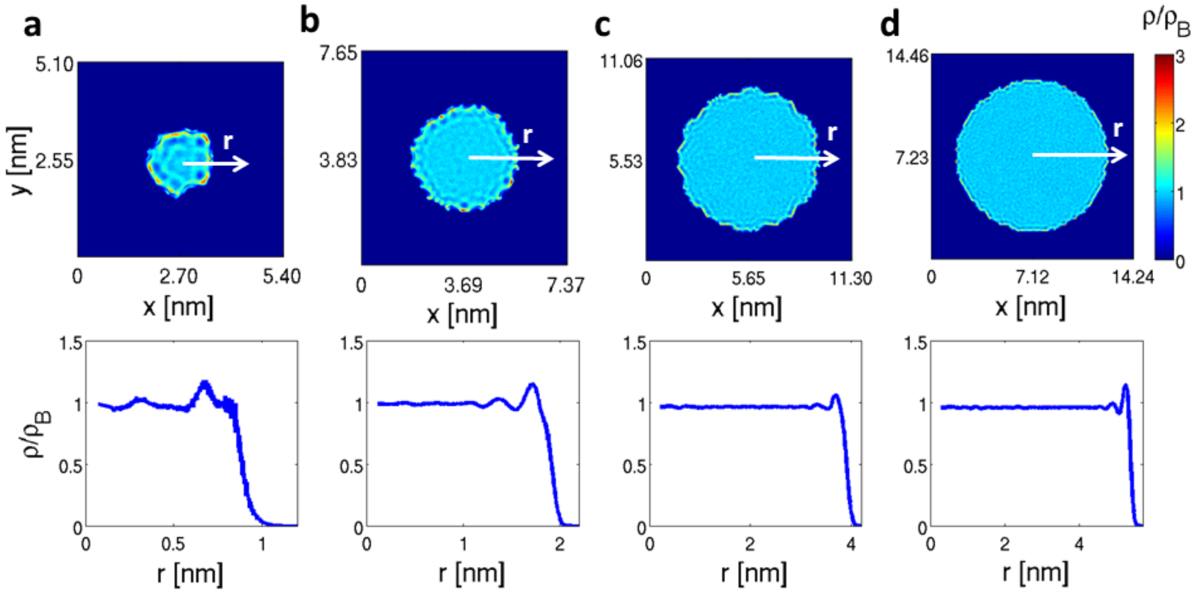

Supplementary Figure 2. Water density ρ within nanopores with different diameters. (a) $\Phi = 2.0$ nm; **(b)** $\Phi = 4.1$ nm; **(c)** $\Phi = 8.1$ nm; **(d)** $\Phi = 11.0$ nm. Top: The 2D distribution of ρ/ρ_B within silica nanopores is depicted, which is calculated by averaging ρ along the nanopore axis. Bottom: The radial ρ/ρ_B within silica nanopores is shown, where $r = 0$ nm lies on nanopore axis.

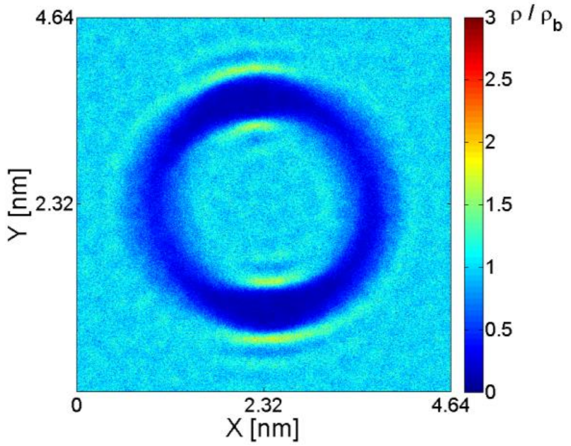

Supplementary Figure 3. Water density ρ within CNT. Density values are normalized by the bulk density $\rho_B = 1000$ kg m^{-3} at 300 K. The 2D distribution of ρ/ρ_B within and around a (20,20) CNT 10 nm long is depicted, which is calculated by averaging ρ along the CNT axis (i.e. Z axis).

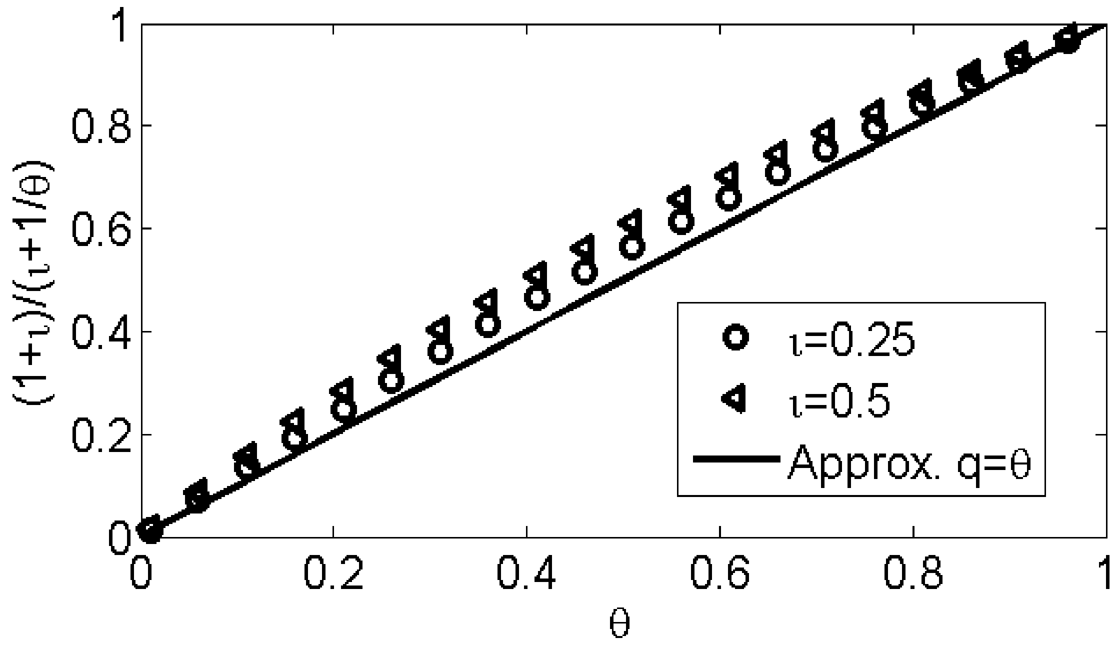

Supplementary Figure 4. Approximation of $q(\theta)$ function. The function $q(\theta) = \frac{1+\iota}{\iota+1/\theta}$ is plotted against the θ scaling variable at two (conservative / large) values of ι (symbols). The approximating function $q(\theta) = \theta$ (line) is also depicted. See **Supplementary Discussion** for further details.

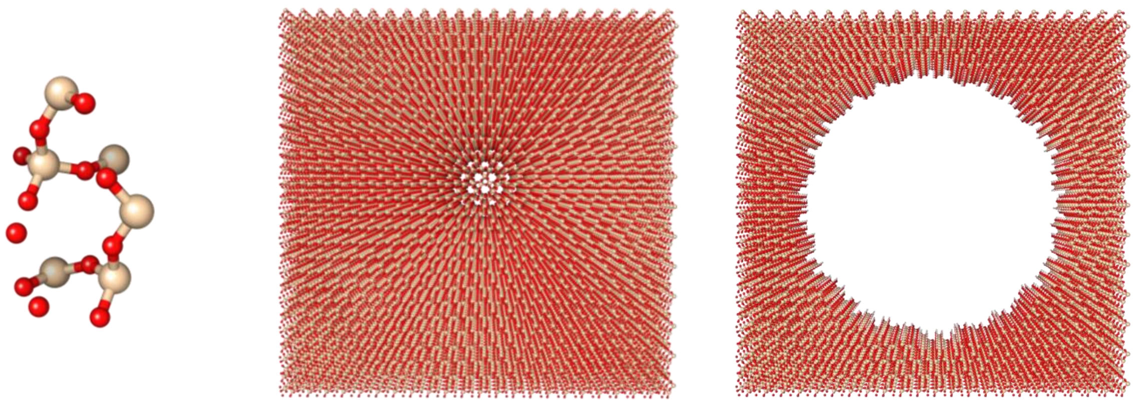

Supplementary Figure 5. Preparation of the MD geometry of a silica nanopore. From left to right: Unit cell of alpha-quartz; silica crystal brick (11.30 x 11.06 x 4.32 nm^3); silica pore (8 nm diameter) before the surface functionalization.

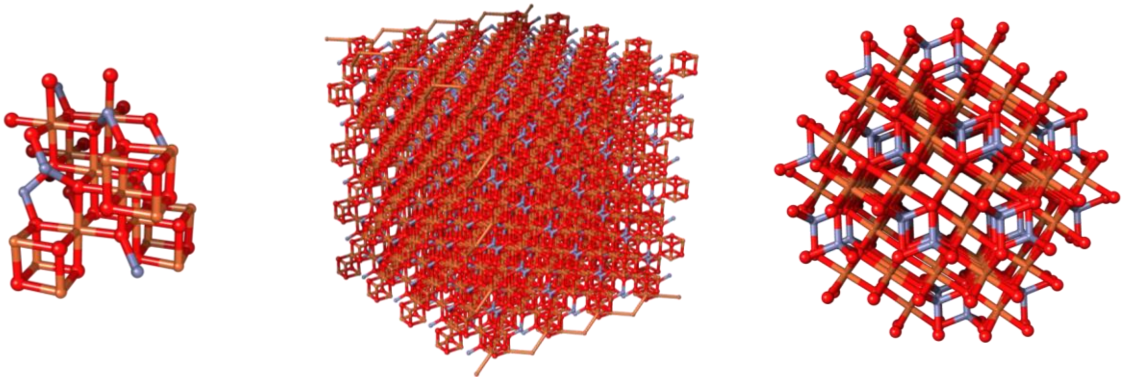

Supplementary Figure 6. Preparation of the MD geometry of a magnetite nanoparticle. From left to right: Unit cell of magnetite; magnetite crystal brick (3.0 x 3.0 x 3.0 nm^3); magnetite nanoparticle (2 nm diameter) before the surface functionalization.

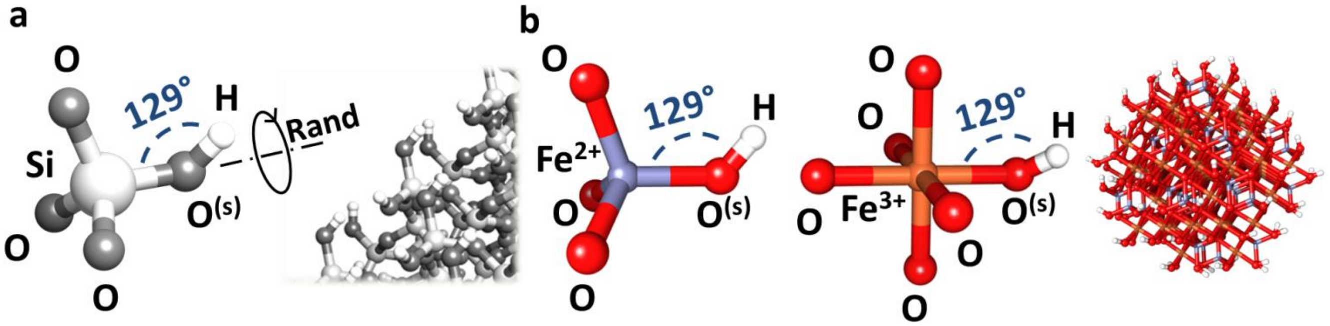

Supplementary Figure 7. Silica and magnetite surface functionalization. **(a)** Silica tetrahedron with geometric details of silanol surface group and magnification of a silica surface. The dihedral angle O-Si-$O^{(s)}$-H is randomly chosen, where $O^{(s)}$ is the oxygen atom belonging to silanol; **(b)** Fe^{2+} tetrahedron and Fe^{3+} octahedron within magnetite crystals, with details of the $Fe^{2+}OH$ and $Fe^{3+}OH$ surface groups. A magnetite particle ($\phi = 2.0$ nm) is shown in the rightmost part.

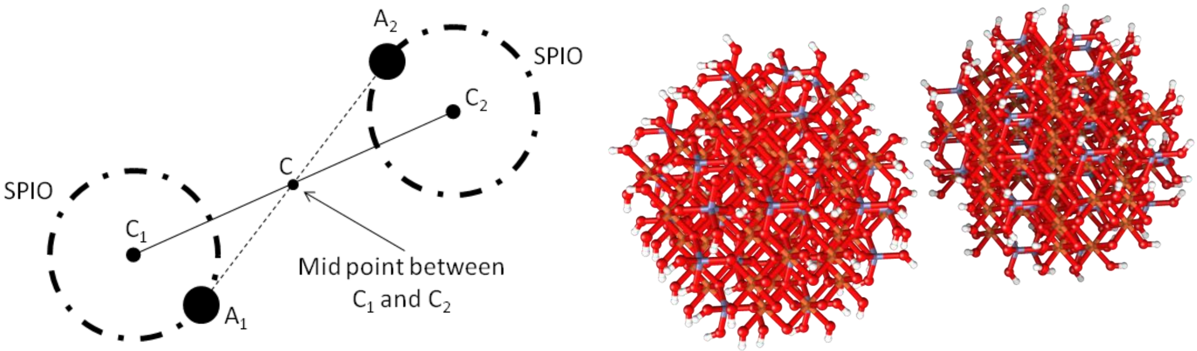

Supplementary Figure 8. Relative positions of NPs couples. NPs are constructed in pairs where each particle represents the mirror image of the other with respect to the midpoint of the line segment connecting the centers of the two particles (on the left-hand side, atom A_1 is the mirror image of A_2 with respect to C). On the right-hand side a rendered image of a SPIO mirrored pair.

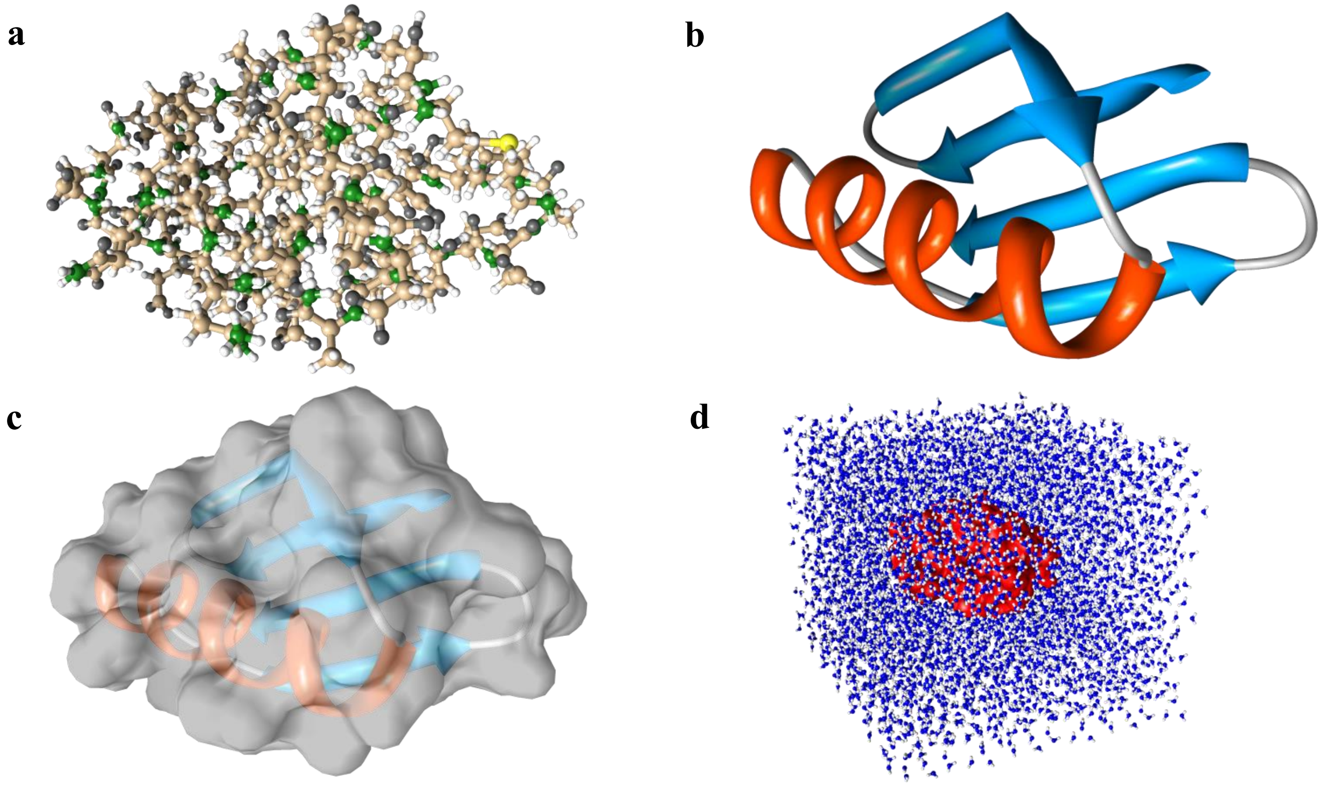

Supplementary Figure 9. MD geometry of B1-Immunoglobulin binding domain. (a) Atomistic visualization. **(b)** Ribbon visualization of secondary structures (orange for alpha-helices; cyan for beta-sheets; gray for random coils). **(c)** Solvent accessible surface of the protein (light gray). **(d)** Protein (red) solvated in SPC/E water box (blue).

.

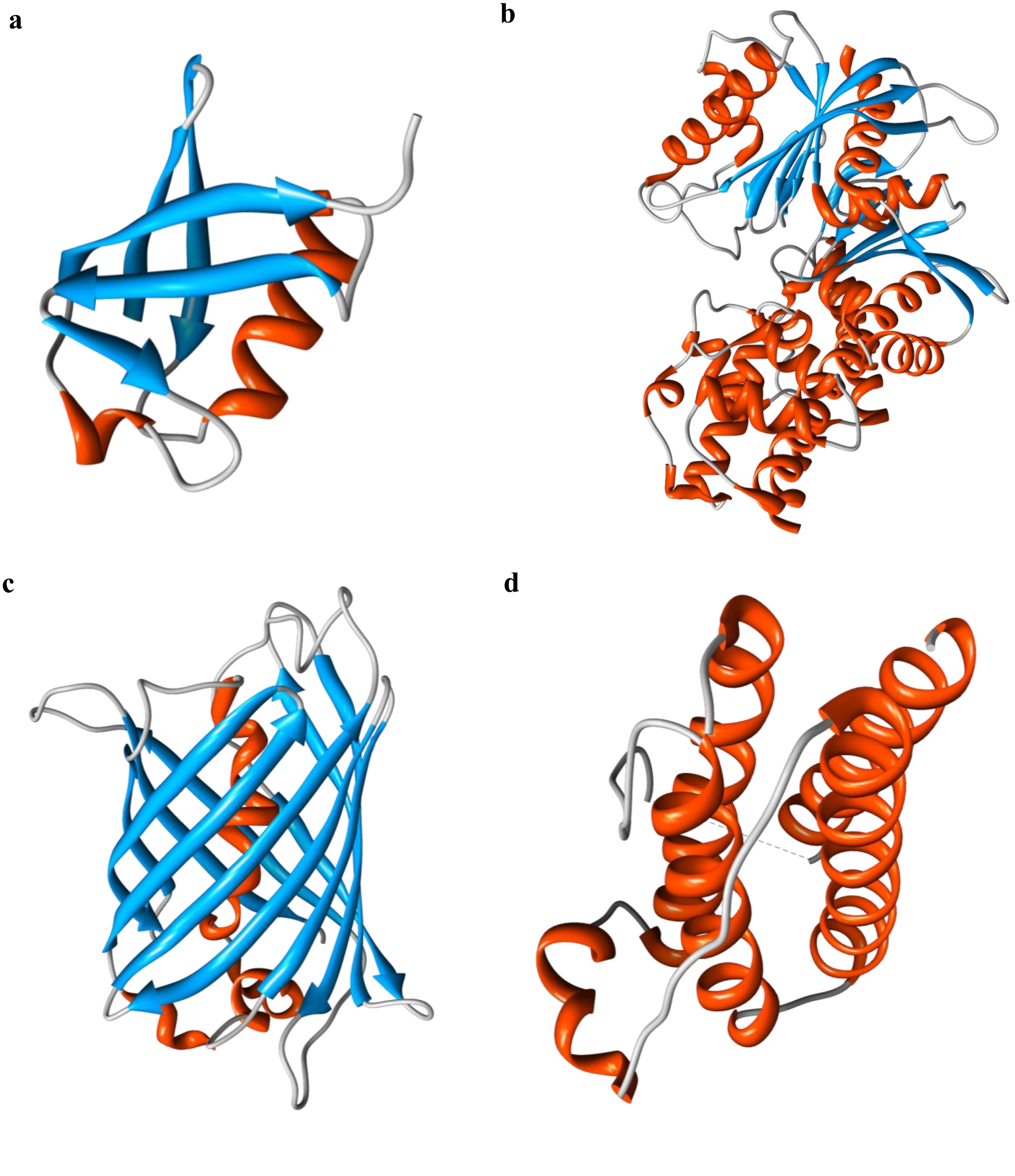
a
b
c
d

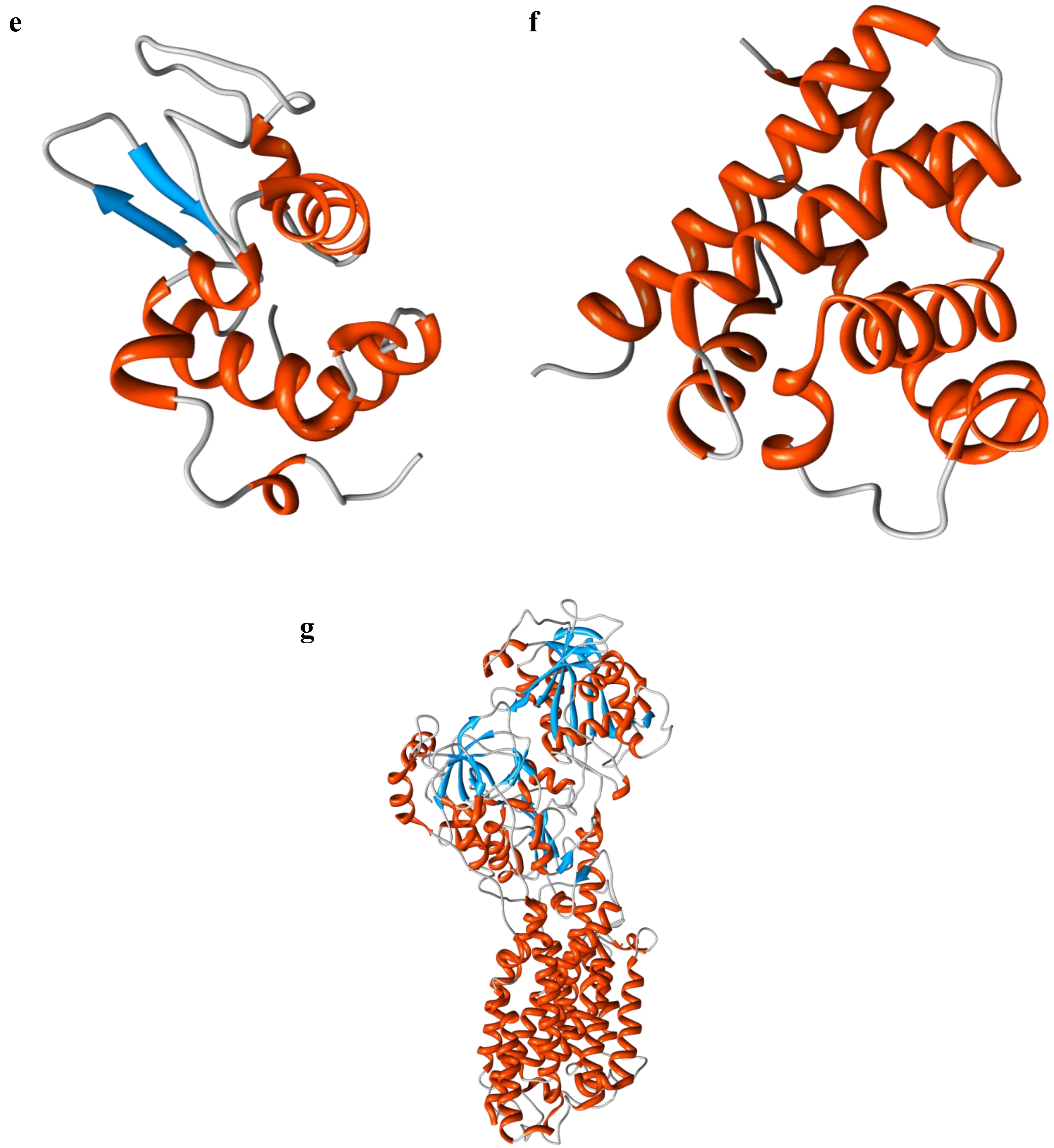

Supplementary Figure 10. Simulated proteins. Secondary structures (orange for alpha-helices; cyan for beta-sheets; gray for random coils) of proteins are displayed**. (a)** Ubiquitin. **(b)** Glucokinase. **(c)** Green Fluorescence. **(d)** Leptin. **(e)** Lysozyme. **(f)** Myoglobin. **(g)** Ca^{2+}-ATPase.

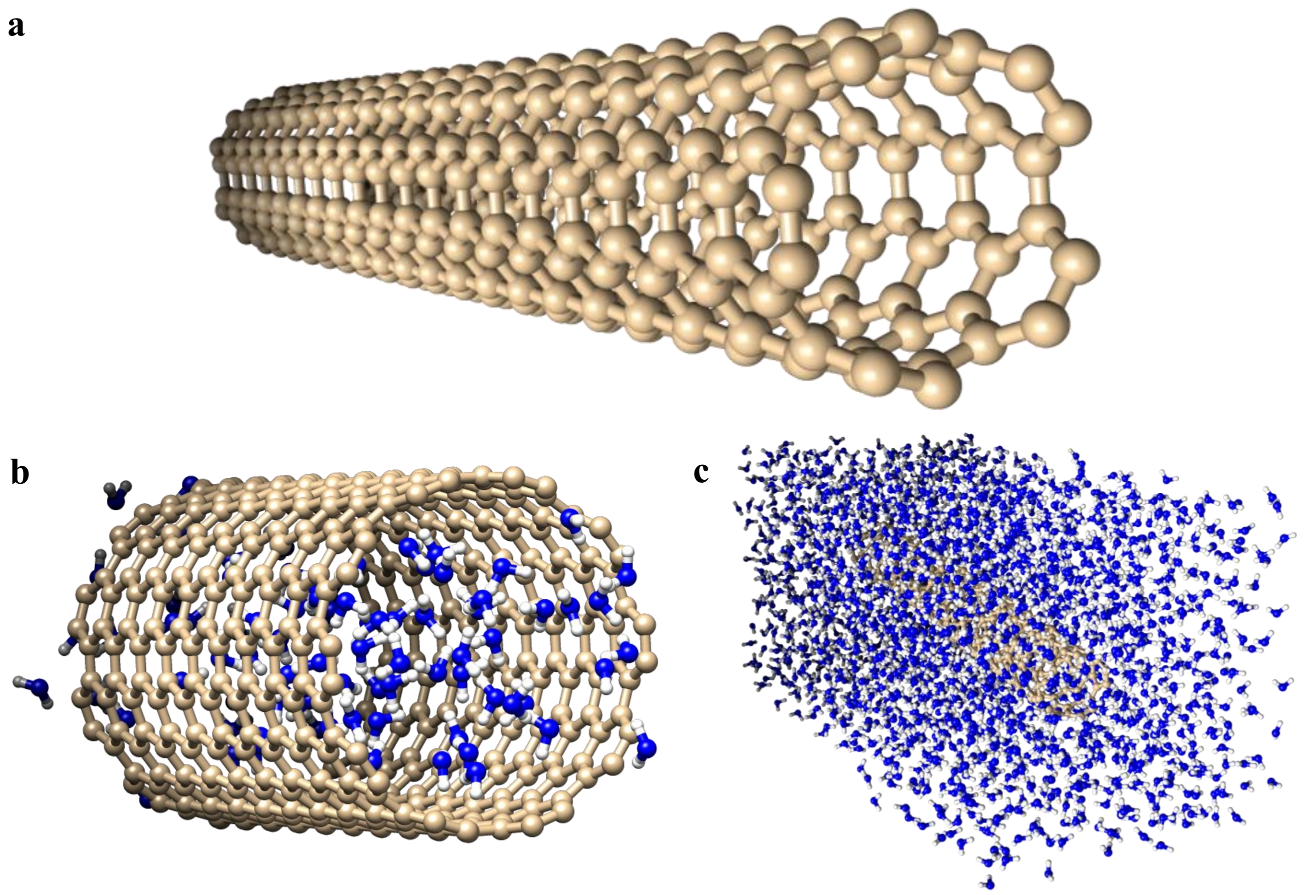

Supplementary Figure 11. MD geometries of solvated carbon nanotubes (CNTs). (a) (5,5) CNT 5 nm long. **(b)** (10,10) CNT filled by water. **(c)** (5,5) CNT 5 nm long solvated in a water box.

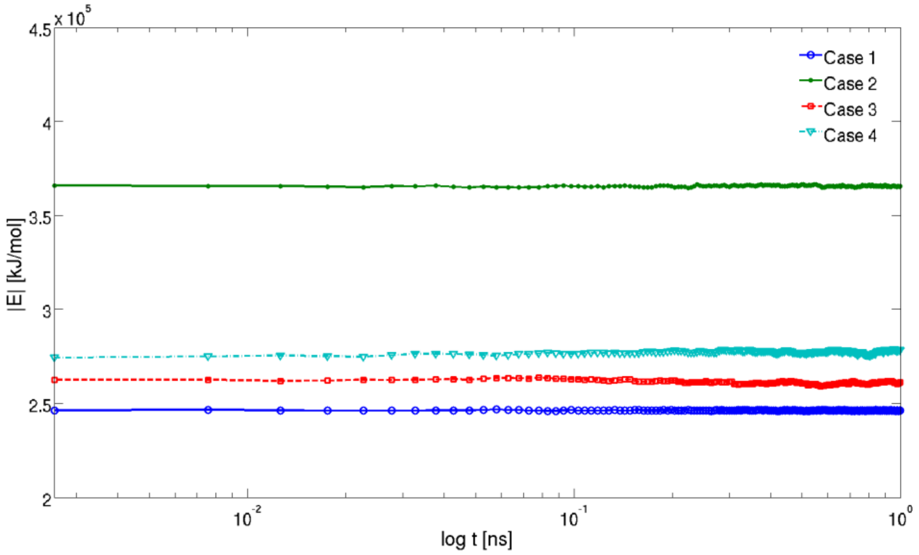

Supplementary Figure 12. Energy trend of nanopore setups. Total energy conservation of the systems where silica nanopores are simulated (Cases 1–4 in **Supplementary Table 1**). *D* is obtained by the MSD calculated between 600 and 1000 ps.

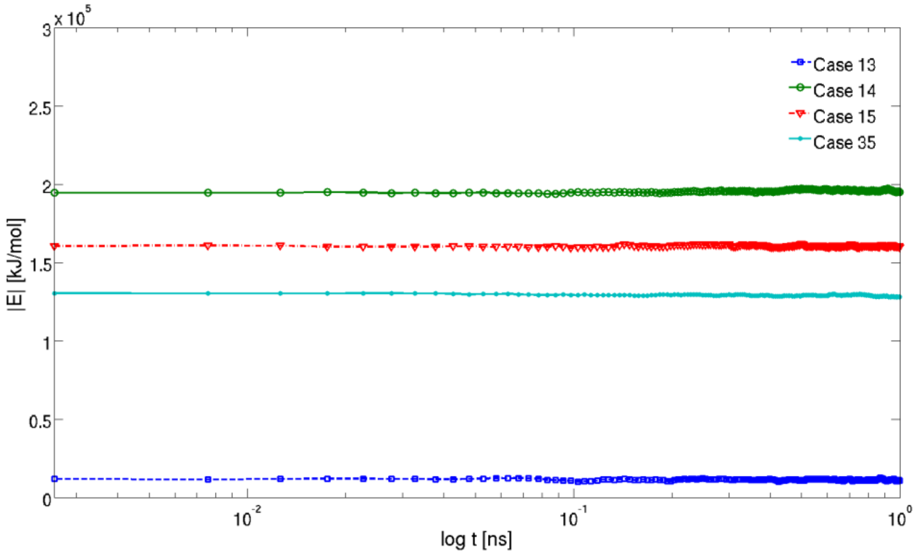

Supplementary Figure 13. Energy trend of NPs setups. Total energy conservation of the systems where silica or magnetite NPs are simulated (Cases 13−15 and 35 in **Supplementary Tables 4** and **8**). D is obtained by the MSD calculated between 600 and 1000 ps.

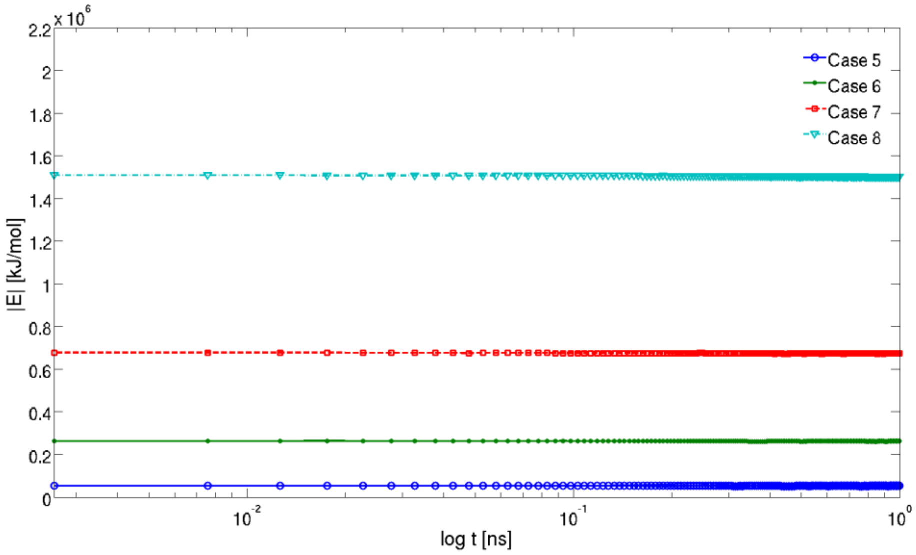

Supplementary Figure 14. Energy trend of nanopore filled by NPs setups. Total energy conservation of the systems where magnetite NPs within silica nanopores are simulated (Cases 5−8 in **Supplementary Table 2**). *D* is obtained by the MSD calculated between 600 and 1000 ps.

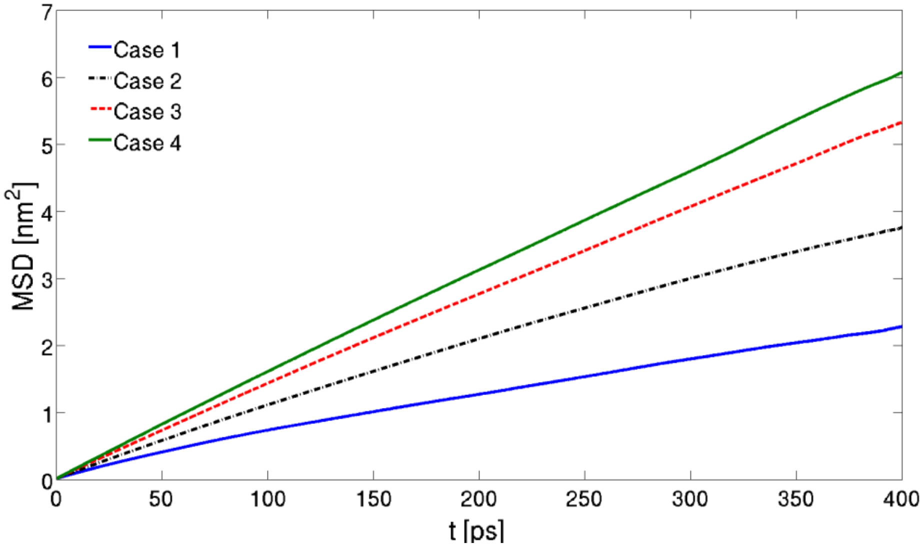

Supplementary Figure 15. MSD of water in nanopore setups. MSD evaluated on equilibrated systems between 600 and 1000 ps. Silica nanopores are simulated (Cases 1–4 in **Supplementary Table 1**).

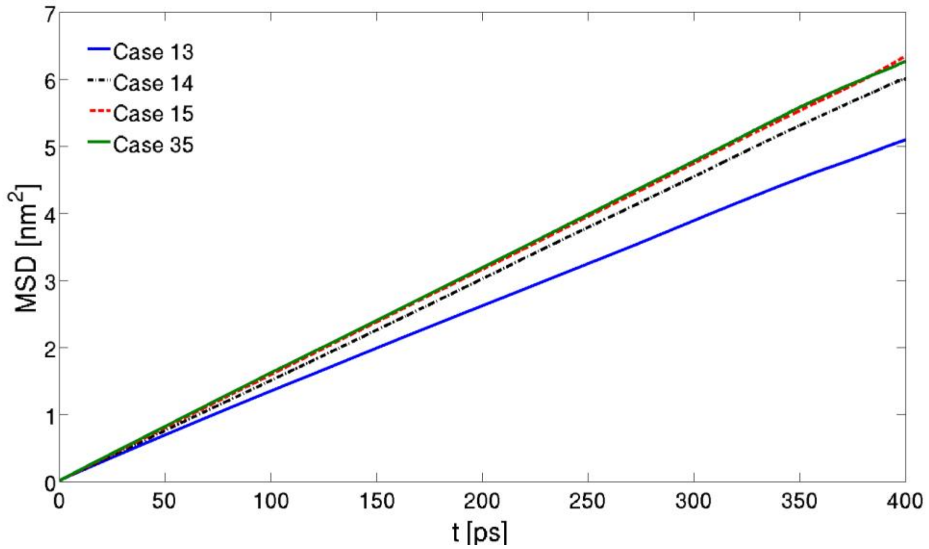

Supplementary Figure 16. MSD of water in NPs setups. MSD evaluated on equilibrated systems between 600 and 1000 ps. Silica or magnetite NPs are simulated (Cases 13−15 and 36 in **Supplementary Tables 4** and **8**).

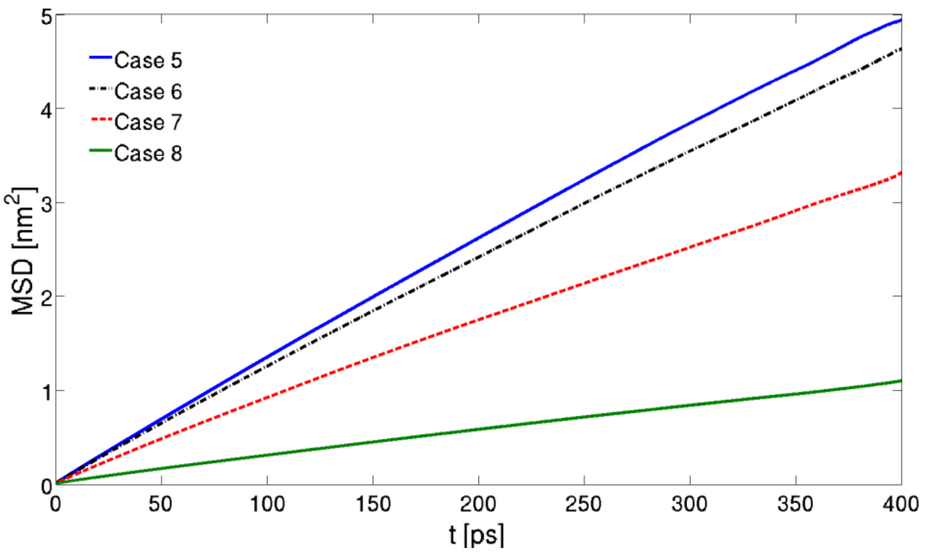

Supplementary Figure 17. MSD of water in nanopore filled by NPs setups. MSD evaluated on equilibrated systems between 600 and 1000 ps. Magnetite NPs within silica nanopores are simulated (Cases 5−8 in **Supplementary Table 2**).

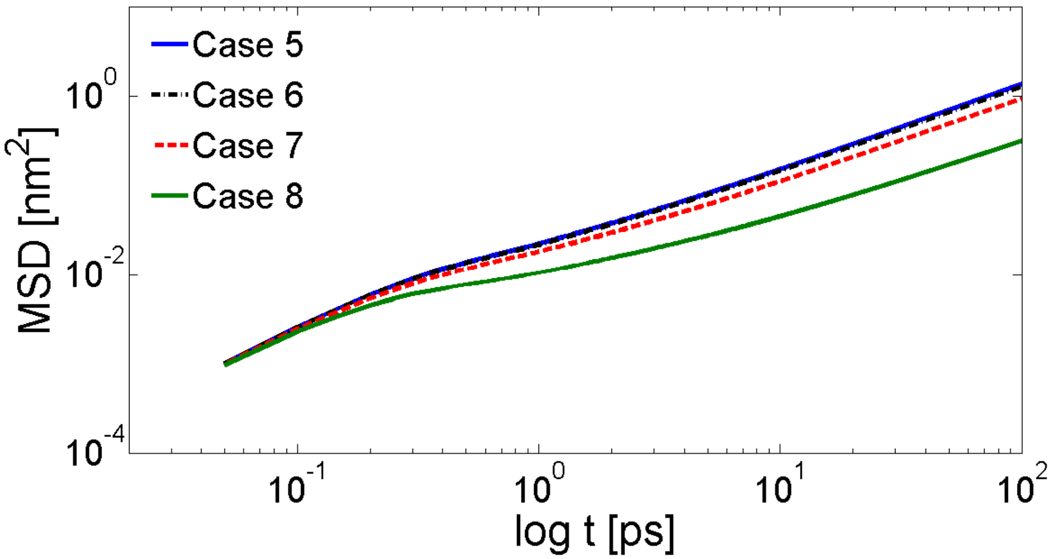

Supplementary Figure 18. logMSD of nanopore filled by NPs setups. Double logarithmic plot of MSD versus time, evaluated on equilibrated systems between 600 and 1000 ps. Magnetite NPs within silica nanopores are simulated (Cases 5−8 in **Supplementary Table 2**).

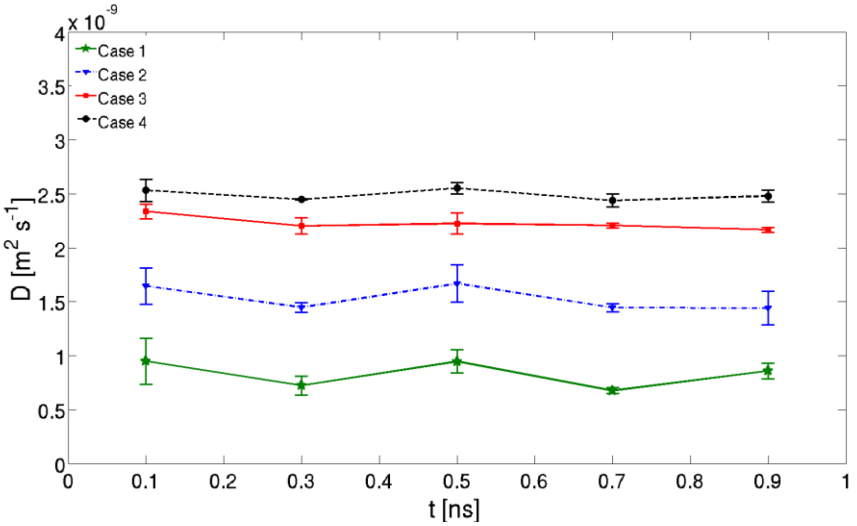

Supplementary Figure 19. *D* versus time in equilibrated nanopore setups. Silica nanopores are simulated (Cases 1−4 in **Supplementary Table 1**). Error bars are obtained by fitting MSD in different time intervals.

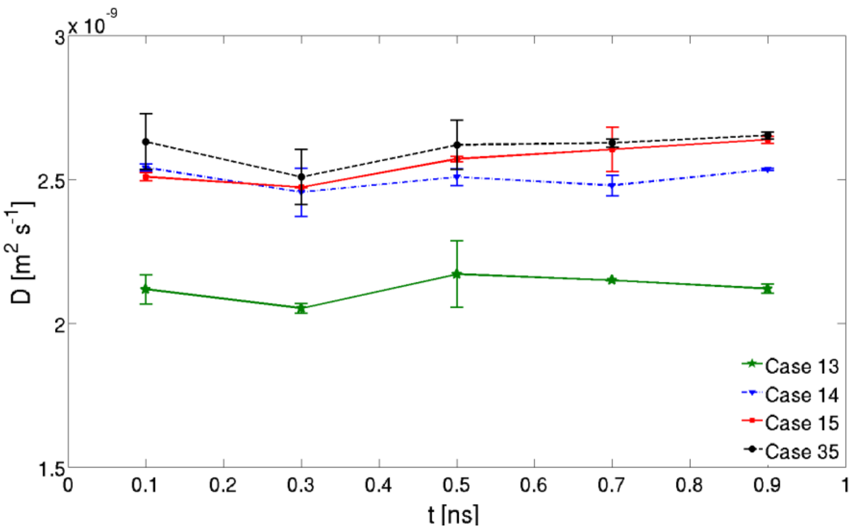

Supplementary Figure 20. D versus time in equilibrated NPs setups. Silica or magnetite NPs are simulated (Cases 13−15 and 36 in **Supplementary Tables 4** and **8**). Error bars are obtained by fitting MSD in different time intervals.

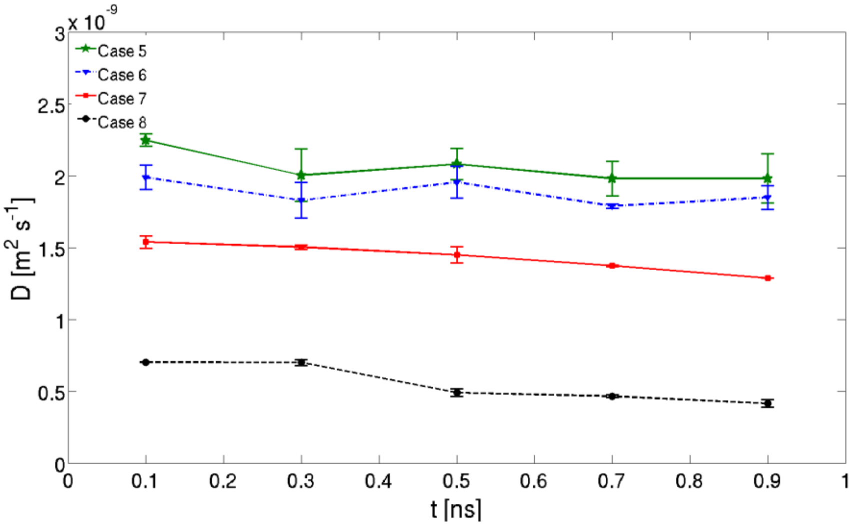

Supplementary Figure 21. D versus time in equilibrated nanopores filled by NPs setups. Magnetite NPs within silica nanopores are simulated (Cases 5−8 in **Supplementary Table 2**). Error bars are obtained by fitting MSD in different time intervals.

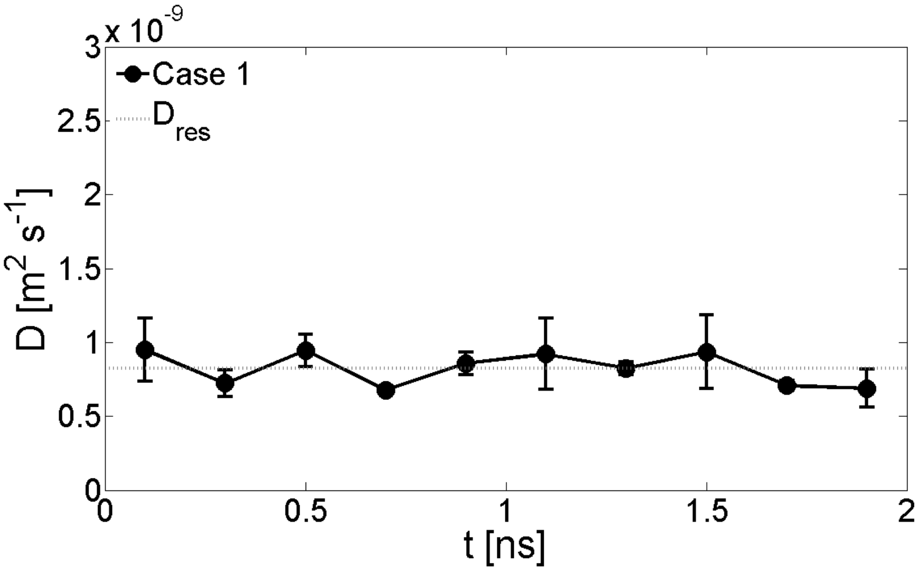

Supplementary Figure 22. *D* versus time in time-extended simulation. Hydrated silica nanopore ($\Phi = 2$ nm) is simulated. D_{res} is the self-diffusion coefficient evaluated in the 0.6-1.0 ns interval, as reported in **Supplementary Table 1**. Error bars are obtained by fitting MSD in different time intervals.

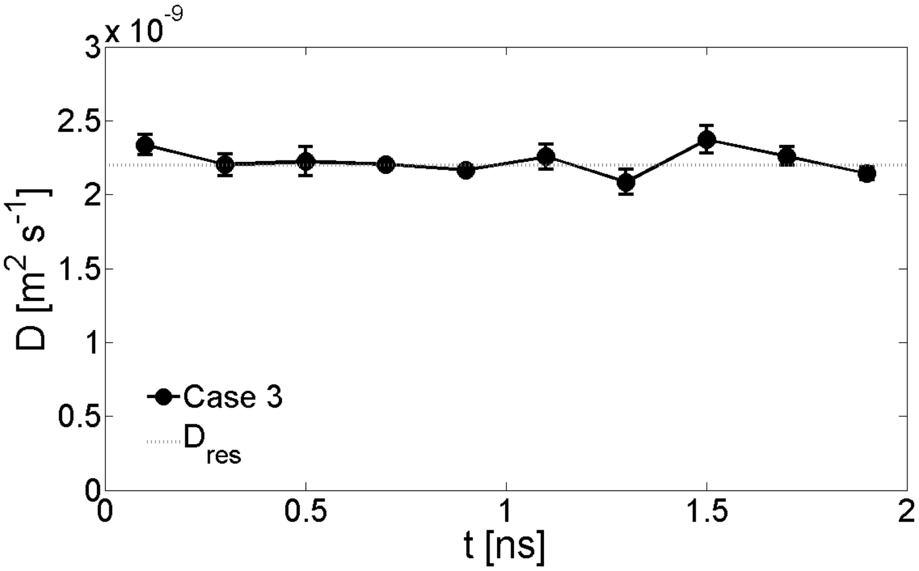

Supplementary Figure 23. *D* versus time in time-extended simulation. Hydrated silica nanopore ($\Phi = 8$ nm) is simulated. D_{res} is the self-diffusion coefficient evaluated in the 0.6-1.0 ns interval, as reported in **Supplementary Table 1**. Error bars are obtained by fitting MSD in different time intervals.

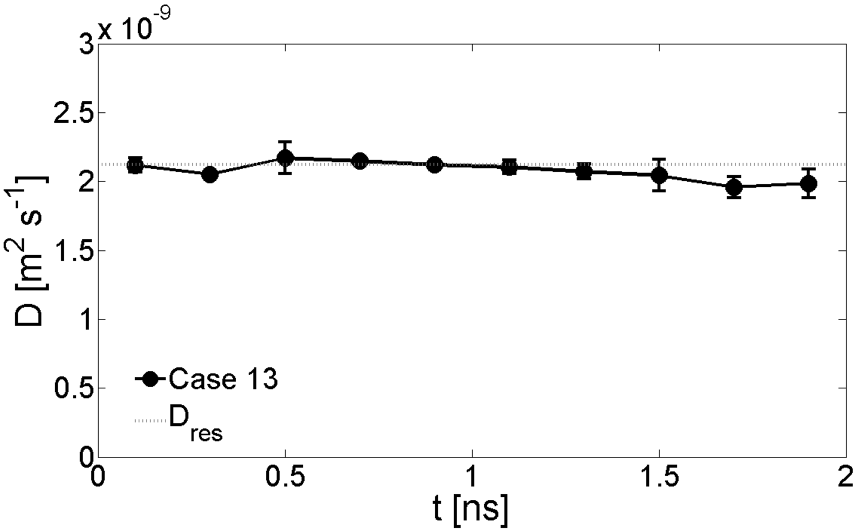

Supplementary Figure 24. *D* versus time in time-extended simulation. Silica nanoparticle (ϕ = 5.2 nm) in a $L = 6$ nm water box is simulated. D_{res} is the self-diffusion coefficient evaluated in the 0.6-1.0 ns interval, as reported in **Supplementary Table 4**. Error bars are obtained by fitting MSD in different time intervals.

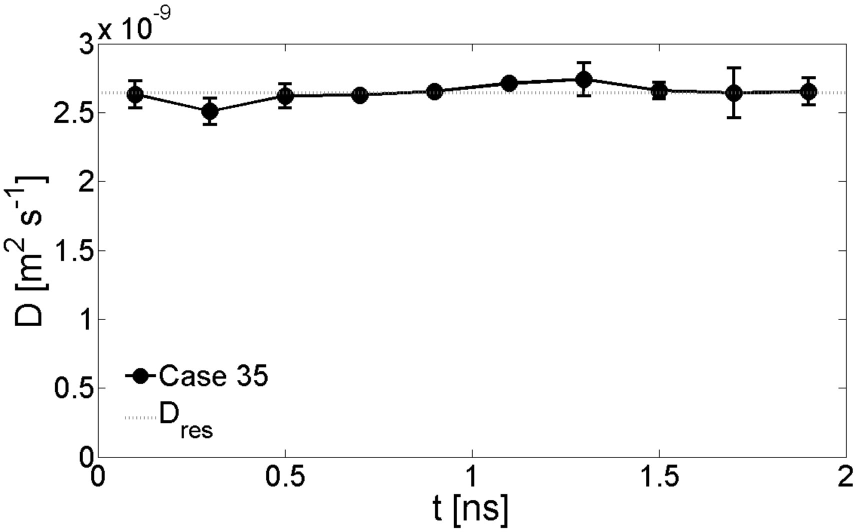

Supplementary Figure 25. *D* versus time in time-extended simulation. Silica nanoparticle (ϕ = 2 nm) in a $L = 5$ nm water box, using the partial charges from magnetite force-field for silica atoms, is simulated. D_{res} is the self-diffusion coefficient evaluated in the 0.6-1.0 ns interval, as reported in **Supplementary Table 8**. Error bars are obtained by fitting MSD in different time intervals.

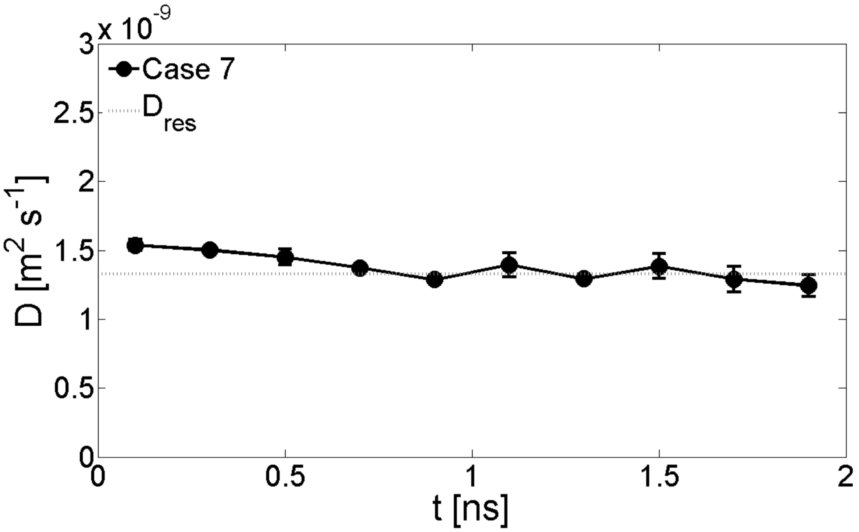

Supplementary Figure 26. *D* versus time in time-extended simulation. Hydrated silica nanopore ($\Phi = 8$ nm) filled by 8 magnetite nanoparticles ($\phi = 2$ nm) is simulated. D_{res} is the self-diffusion coefficient evaluated in the 0.6-1.0 ns interval, as reported in **Supplementary Table 2**. Error bars are obtained by fitting MSD in different time intervals.

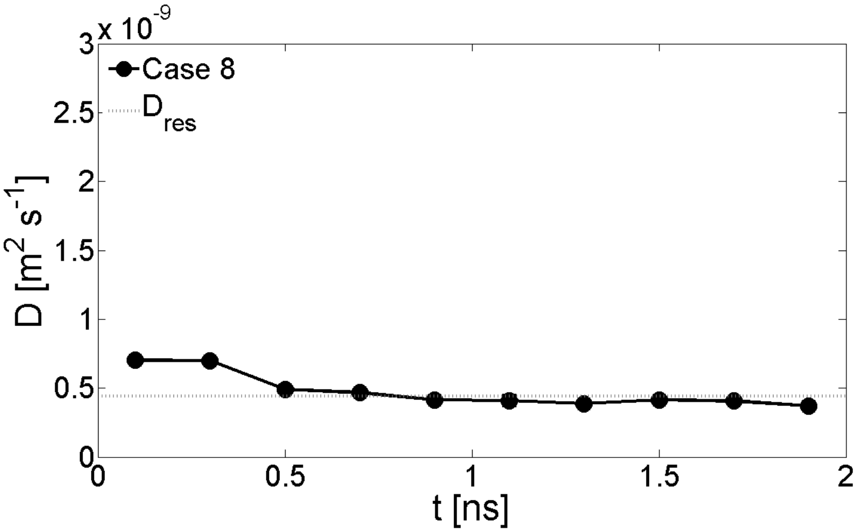

Supplementary Figure 27. *D* versus time in time-extended simulation. Hydrated silica nanopore (Φ = 8 nm) filled by 16 magnetite nanoparticles (ϕ = 2 nm) is simulated. D_{res} is the self-diffusion coefficient evaluated in the 0.6-1.0 ns interval, as reported in **Supplementary Table 2**. Error bars are obtained by fitting MSD in different time intervals.

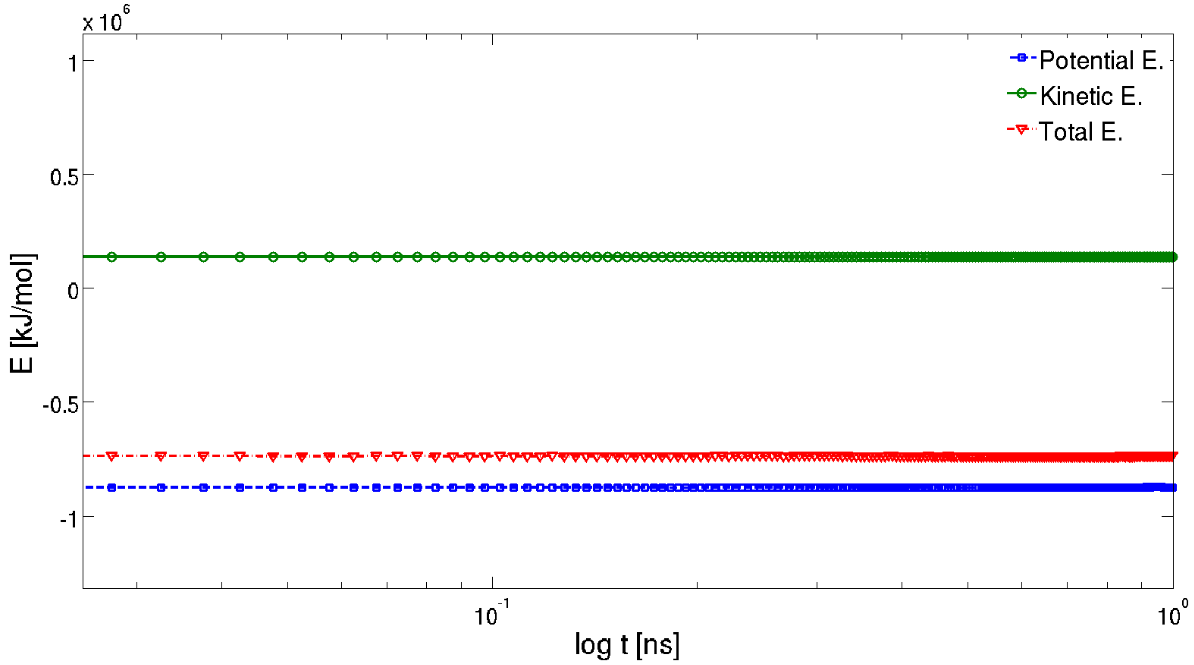

Supplementary Figure 28. Energies trend in 1 ns MD simulation of a solvated protein. As an example, potential, kinetic and total energies from the Glucokinase simulation (Supplementary Figure 6 b) are depicted.

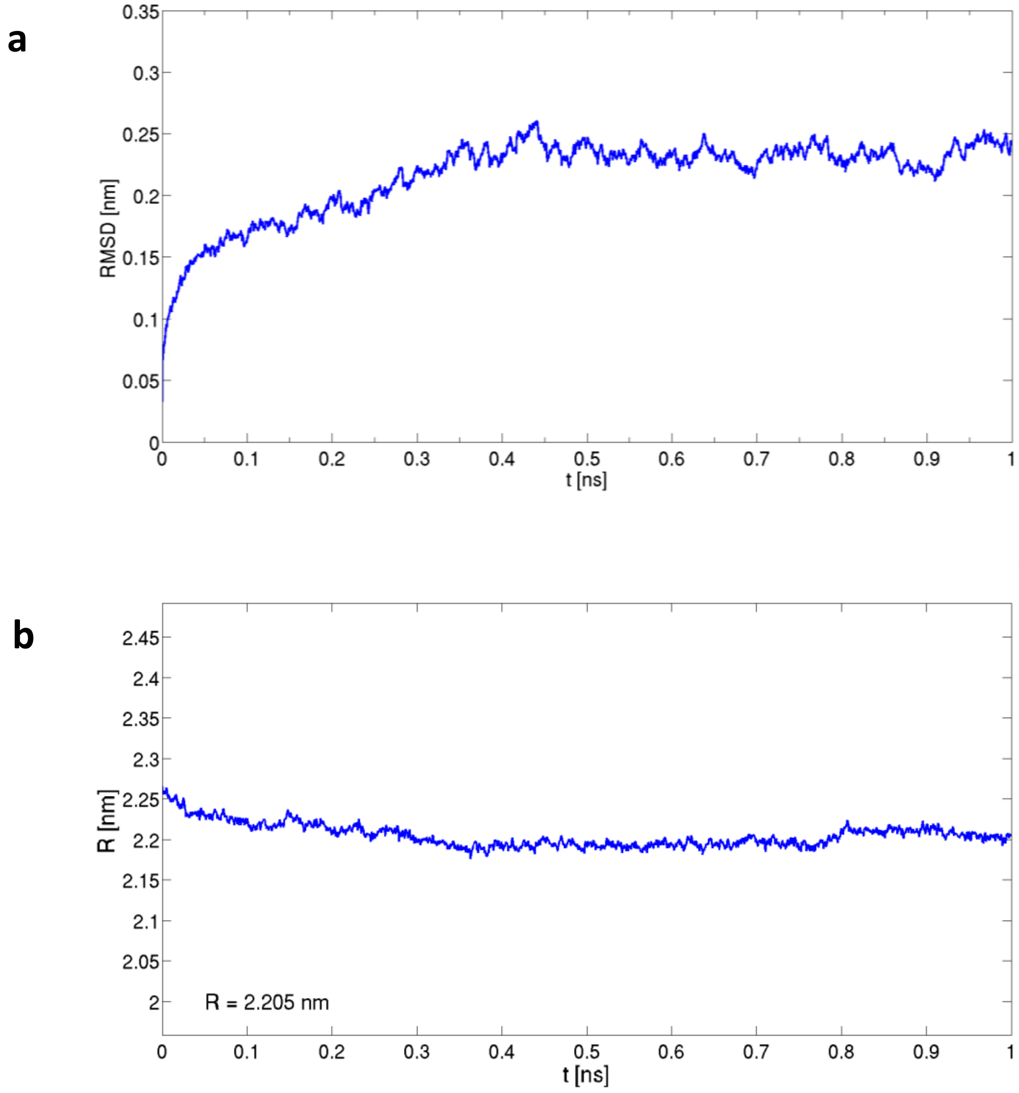

Supplementary Figure 29. Structure stability of Glucokinase in 1 ns MD simulation. (a) Root mean square deviation**. (b)** Radius of gyration.

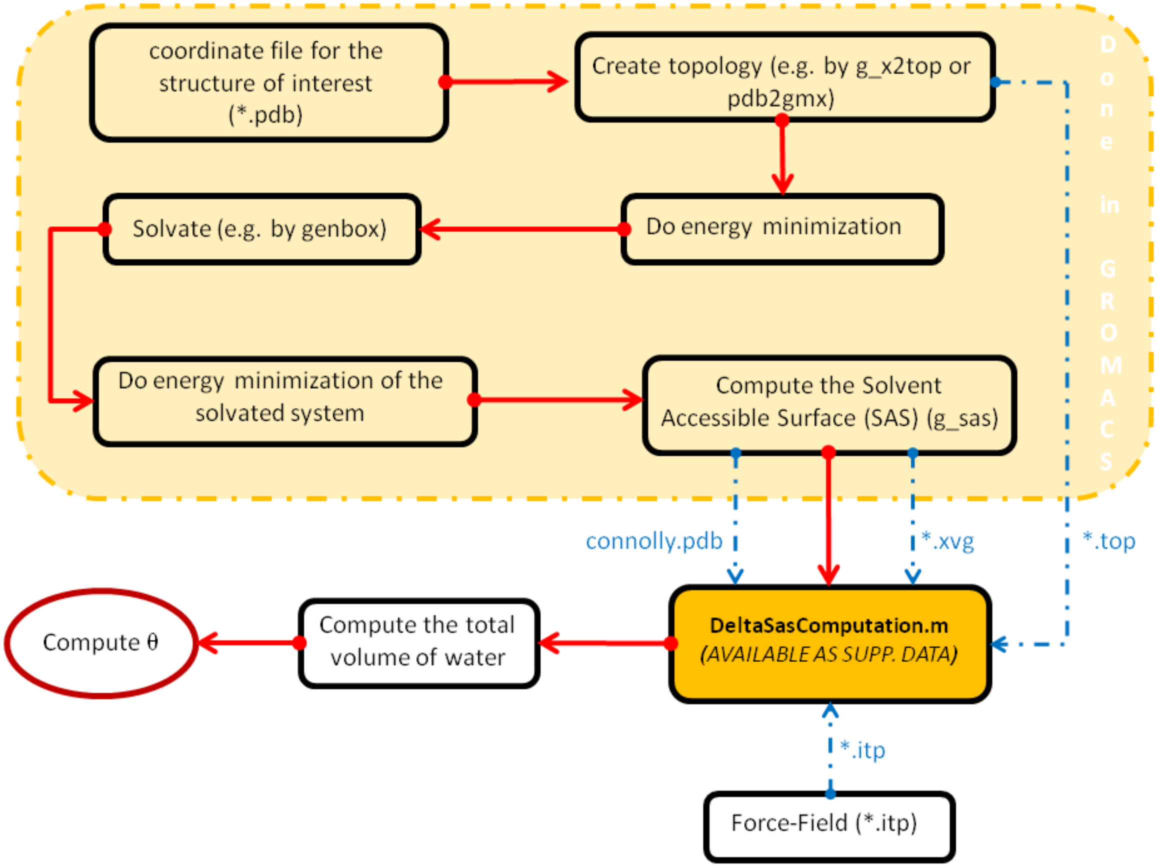

Supplementary Figure 30. Steps leading to the computation of the scaling parameter *θ*. Red arrows indicate the chronological sequence, while dashed blue lines indicate the flow of files provided as **Supplementary Software 1**.

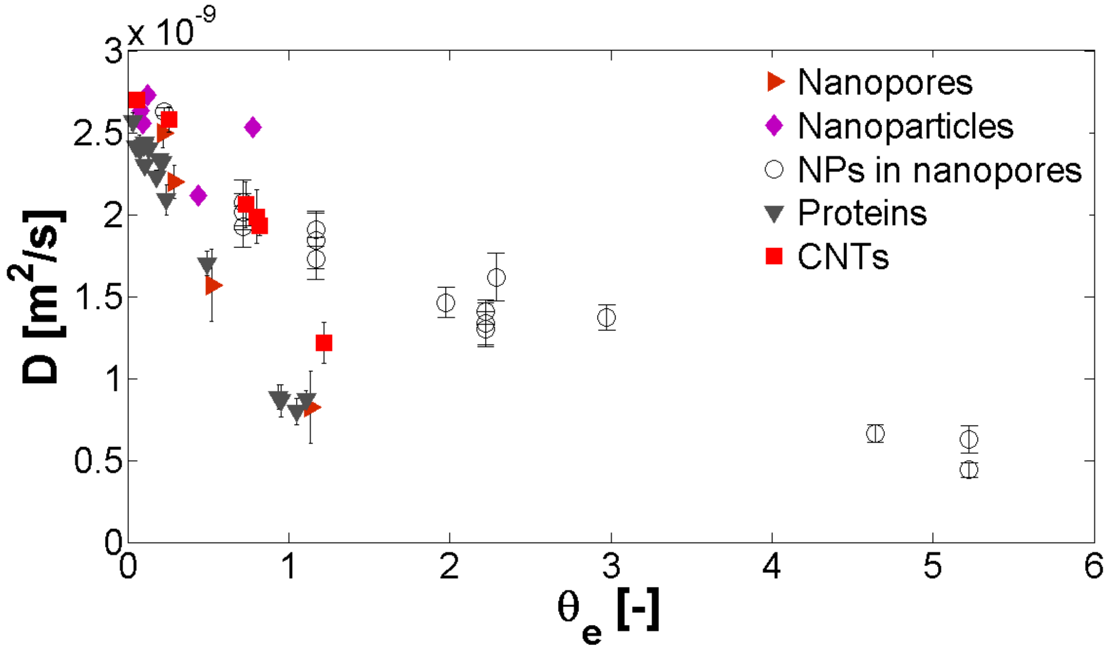

Supplementary Figure 31. Alternative dimensionless parameter $\boldsymbol{\theta_e}$. D self-diffusion coefficient of water vs. the dimensionless parameter θ_e based on the interfacial work (see **Supplementary Discussion**). For simplicity, only a subset of the analyzed cases are reported.

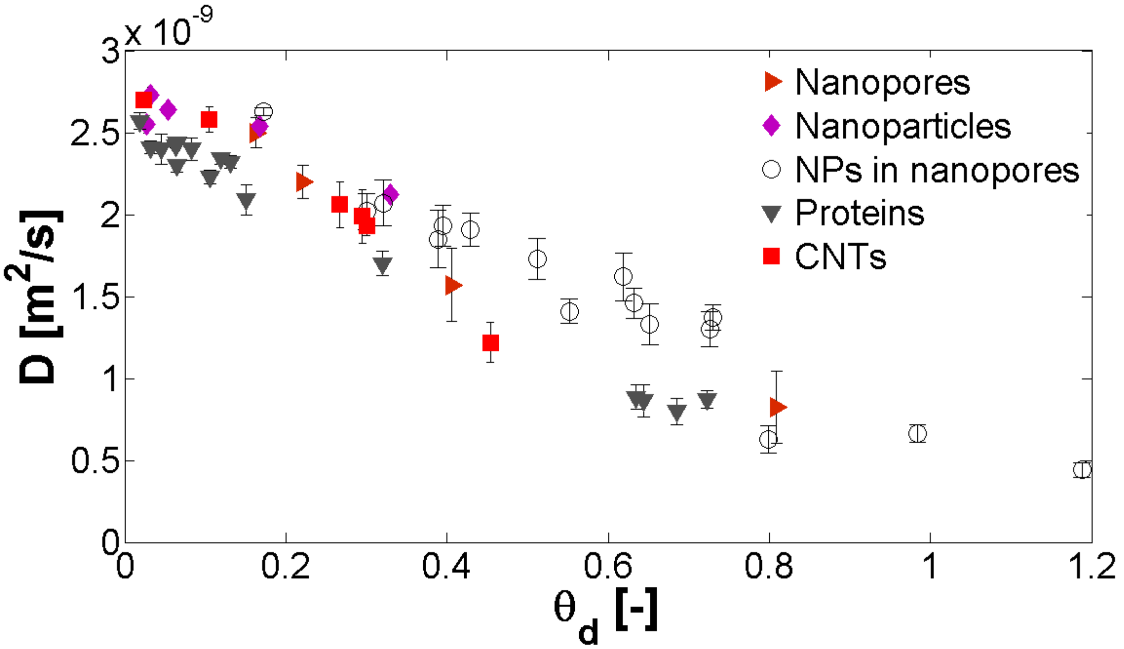

Supplementary Figure 32. Alternative dimensionless parameter $\boldsymbol{\theta_d}$. D self-diffusion coefficient of water vs. the dimensionless parameter θ_d, which takes into account also the density of water of the system (see **Supplementary Discussion**). For simplicity, only a subset of the analyzed cases are reported.

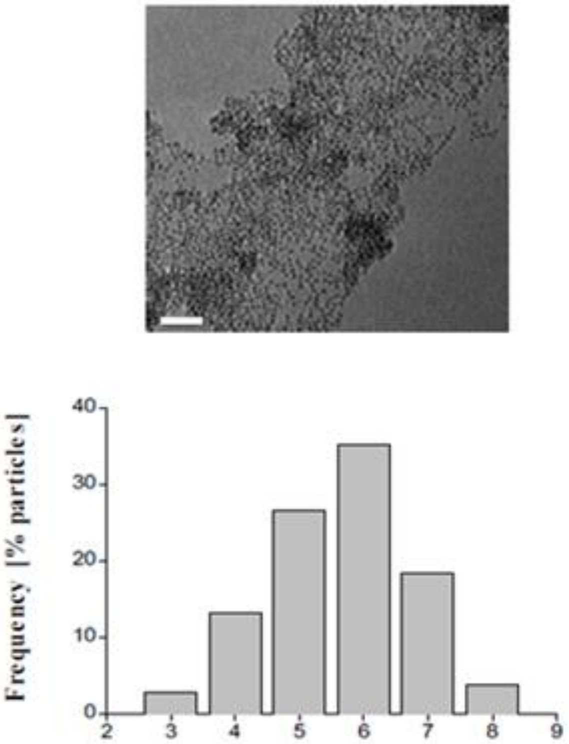

Supplementary Figure 33. SPIOs characterization. TEM images (top row) of the (5 nm) SPIOs and size distribution histograms (bottom row). Scale bar is 50 nm.

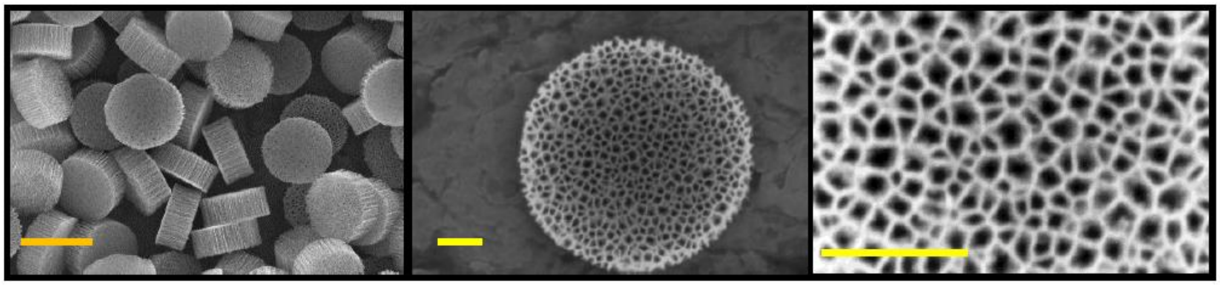

Supplementary Figure 34. SEM images of the mesoporous silicon particles (SiMPs). SiMPs present a diameter of 1,000 nm, a height of 400 nm and an average pore size of 40 nm. Scale bars are 1,000 nm (left) and 200 nm (center and right).

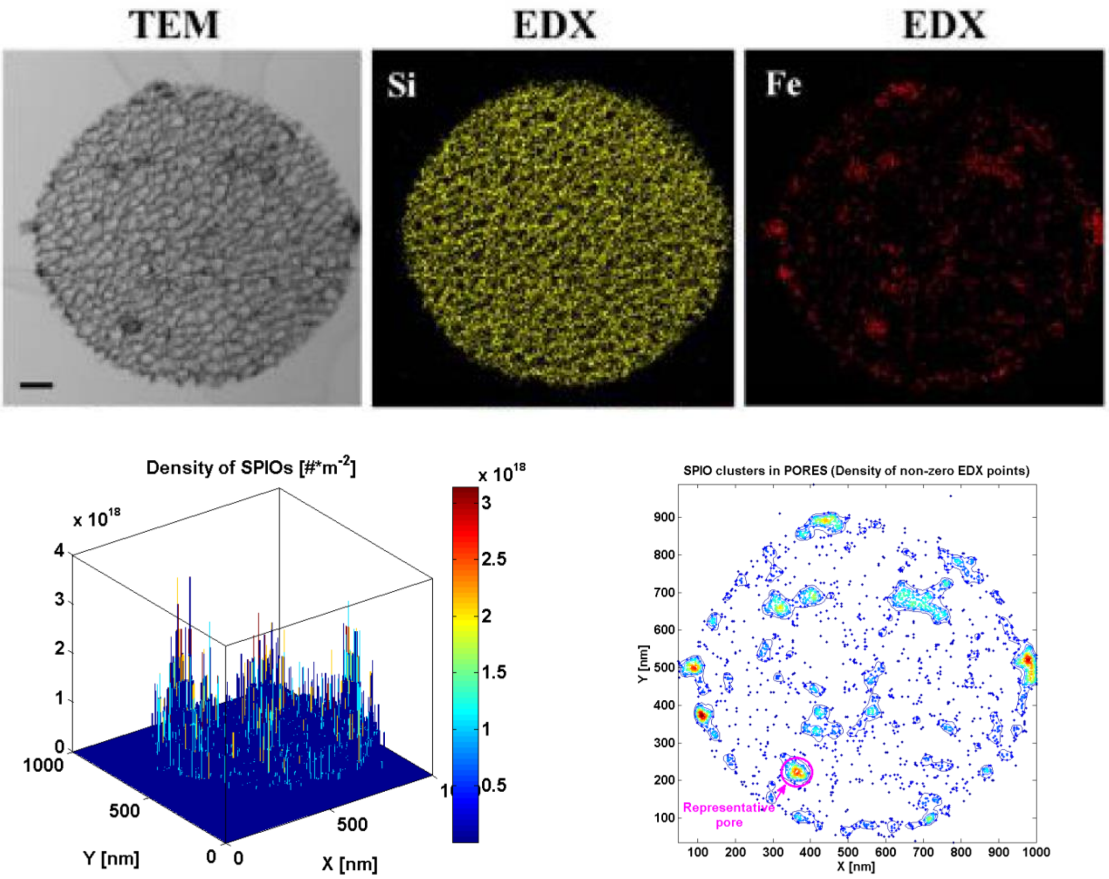

Supplementary Figure 35. Density distribution of SPIOs within a SiMP. TEM, EDX image (silicon) and EDX image (iron) of a representative SiMP (Top). Local density of SPIOs (bottom-left). Density of non-zero EDX points (bottom-right).

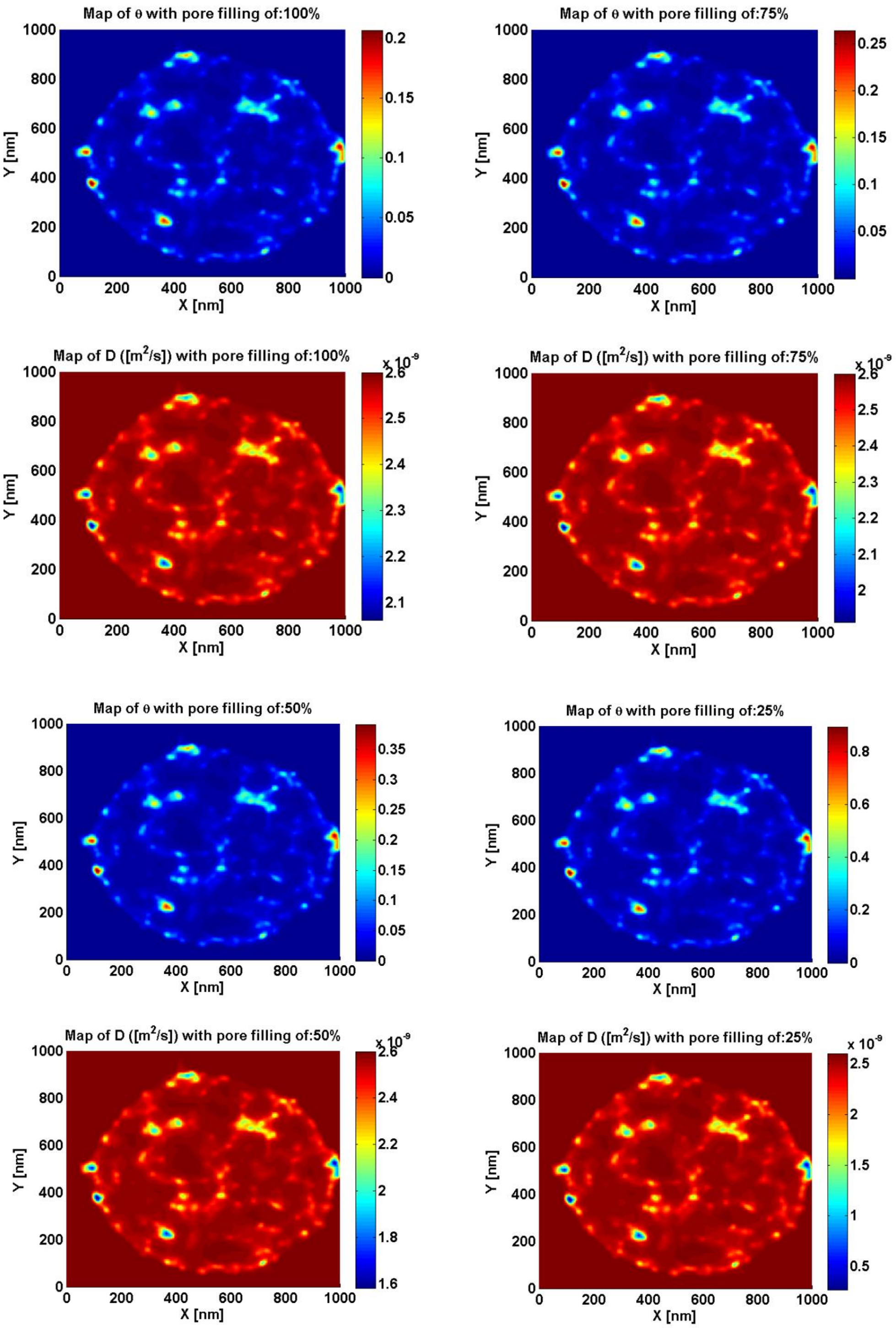

Supplementary Figure 36. $\boldsymbol{\theta}$ and $\boldsymbol{D}$ maps in a SiMP filled by SPIOs at different fillings. Map of the scaling parameter $\theta = \theta(X, Y)$ starting from the EDX image in **Supplementary Figure 35** at several pore loadings / fillings (i.e. pore fraction occupied by SPIOs).

SUPPLEMENTARY TABLES

Supplementary Table 1. MD simulations and results of silica nanopores with different diameters Φ.

CASE	Geometry			Thermodynamics		Force-Field		Characteristic length	θ	D [m^2 s^{-1}]	+/- [m^2 s^{-1}]
	Φ [nm]	ϕ [nm]	N	ρ [kg m^{-3}]	T [K]	Partial Charges	ε_{Fe} [kJ mol^{-1}]	δ [nm]			
**1**	2.03	-	0	818	300	Default	-	0.367	**0.663**	**8.24E-10**	2.20E-10
**2**	4.05	-	0	903	300	Default	-	0.333	**0.366**	**1.57E-09**	2.22E-10
**3**	8.13	-	0	913	300	Default	-	0.331	**0.202**	**2.20E-09**	1.01E-10
**4**	11.04	-	0	923	300	Default	-	0.329	**0.150**	**2.50E-09**	8.98E-11

Supplementary Table 2. MD simulations and results of silica nanopores (Φ = 8.1 nm) filled by a varying number N of magnetite NPs (ϕ = 2.0 nm).

CASE	Geometry			Thermodynamics		Force-Field		Characteristic lengths	θ	D [m^2 s^{-1}]	+/- [m^2 s^{-1}]
	Φ [nm]	ϕ [nm]	N	ρ [kg m^{-3}]	T [K]	Partial Charges	ε_{Fe} [kJ mol^{-1}]	δ [nm]			
**5**	8.13	1.97	2	859	300	Default	24.94	0.331 (sil.)/ 0.497 (mag.)	**0.276**	**2.07E-09**	1.41E-10
**6**	8.13	1.97	4	838	300	Default	24.94	0.331 (sil.)/ 0.497 (mag.)	**0.360**	**1.91E-09**	1.01E-10
**7**	8.13	1.97	8	797	300	Default	24.94	0.331 (sil.)/ 0.497 (mag.)	**0.519**	**1.33E-09**	1.27E-10
**8**	8.13	1.97	16	663	300	Default	24.94	0.331 (sil.)/ 0.497 (mag.)	**0.787**	**4.41E-10**	4.49E-11

Supplementary Table 3. MD simulations and results of silica nanopores with diameter Φ filled by N magnetite NPs with diameter ϕ.

CASE	Geometry			Thermodynamics		Force-Field		Characteristic lengths	θ	D [m^2 s^{-1}]	+/- [m^2 s^{-1}]
	Φ [nm]	ϕ [nm]	N	ρ [kg m^{-3}]	T [K]	Partial Charges	ε_{Fe} [kJ mol^{-1}]	δ [nm]			
**9**	8.13	1.27	16	812	300	Default	24.94	0.331 (sil.)/ 0.461 (mag.)	**0.513**	**1.46E-09**	9.15E-11
**10**	11.04	1.27	36	873	300	Default	24.94	0.329 (sil.)/ 0.461 (mag.)	**0.540**	**1.62E-09**	1.46E-10
**11**	11.04	1.97	20	808	300	Default	24.94	0.329 (sil.)/ 0.497 (mag.)	**0.590**	**1.37E-09**	7.79E-11
**12**	11.04	1.27	66	786	300	Default	24.94	0.329 (sil.)/ 0.461 (mag.)	**0.774**	**6.63E-10**	5.14E-11

Supplementary Table 4. MD simulations and results of a silica or magnetite NP with diameter ϕ immersed in a cubic box of water with edge L.

CASE	Geometry				Thermodynamics		Force-Field		Characteristic length	θ	D [m^2 s^{-1}]	+/- [m^2 s^{-1}]
	Mat.	ϕ [nm]	L [nm]	N	ρ [kg m^{-3}]	T [K]	Partial Charges	ε_{Fe} [kJ mol^{-1}]	δ [nm]			
**13**	SiO_2	5.22	6	1	935	300	Default	-	0.327	**0.309**	**2.12E-09**	3.78E-11
**14**	Fe_3O_4	5.22	8	1	974	300	Default	24.94	0.533	**0.164**	**2.53E-09**	3.54E-11
**15**	SiO_2	5.22	7	1	926	300	Default	-	0.327	**0.159**	**2.63E-09**	2.32E-11
**16**	Fe_3O_4	1.97	7	1	991	300	Default	24.94	0.461	**0.032**	**2.73E-09**	4.93E-11
**17**	Fe_3O_4	1.27	6	1	988	300	Default	24.94	0.497	**0.027**	**2.55E-09**	5.32E-11

Supplementary Table 5. MD simulations and results of silica nanopores (Φ = 8.1 nm) filled by a varying number N of magnetite NPs (ϕ = 2.0 nm), according to different densities of water ρ within the nanopore.

CASE	Geometry			Thermodynamics		Force-Field		Characteristic lengths	θ	D [m² s⁻¹]	+/- [m² s⁻¹]
	Φ [nm]	ϕ [nm]	N	ρ [kg m⁻³]	T [K]	Partial Charges	ε_{Fe} [kJ mol⁻¹]	δ [nm]			
**18**	8.13	1.97	2	919	300	Default	24.94	0.331 (sil.)/ 0.497 (mag.)	**0.276**	**2.02E-09**	1.09E-10
**19**	8.13	1.97	4	925	300	Default	24.94	0.331 (sil.)/ 0.497 (mag.)	**0.360**	**1.85E-09**	1.75E-10
**20**	8.13	1.97	8	941	300	Default	24.94	0.331 (sil.)/ 0.497 (mag.)	**0.519**	**1.40E-09**	7.33E-11
**21**	8.13	1.97	16	985	300	Default	24.94	0.331 (sil.)/ 0.497 (mag.)	**0.787**	**6.29E-10**	8.38E-11
**22**	8.13	1.97	2	698	300	Default	24.94	0.331 (sil.)/ 0.497 (mag.)	**0.276**	**1.92E-09**	1.25E-10
**23**	8.13	1.97	4	702	300	Default	24.94	0.331 (sil.)/ 0.497 (mag.)	**0.360**	**1.73E-09**	1.27E-10
**24**	8.13	1.97	8	715	300	Default	24.94	0.331 (sil.)/ 0.497 (mag.)	**0.519**	**1.30E-09**	1.06E-10

Supplementary Table 6. MD simulations and results of silica nanopores (Φ = 8.1 nm) filled by a varying number N of magnetite NPs (ϕ = 2.0 nm), according to different values of Lennard-Jones potential of iron atoms ε_{Fe}.

CASE	Geometry			Thermodynamics		Force-Field		Characteristic lengths	θ	D [m^2 s^{-1}]	+/- [m^2 s^{-1}]
	Φ [nm]	ϕ [nm]	N	ρ [kg m^{-3}]	T [K]	Partial Charges	ε_{Fe} [kJ mol^{-1}]	δ [nm]			
**25**	8.13	1.97	2	859	300	Default	2.49	0.331 (sil.)/ 0.447 (mag.)	**0.256**	**2.16E-09**	2.25E-10
**26**	8.13	1.97	2	859	300	Default	12.47	0.331 (sil.)/ 0.496 (mag.)	**0.268**	**2.07E-09**	1.46E-10
**27**	8.13	1.97	4	838	300	Default	2.49	0.331 (sil.)/ 0.447 (mag.)	**0.322**	**1.92E-09**	6.64E-11
**28**	8.13	1.97	4	838	300	Default	12.47	0.331 (sil.)/ 0.496 (mag.)	**0.346**	**1.91E-09**	1.12E-10
**29**	8.13	1.97	8	797	300	Default	2.49	0.331 (sil.)/ 0.447 (mag.)	**0.453**	**1.47E-09**	1.09E-10
**30**	8.13	1.97	8	797	300	Default	12.47	0.331 (sil.)/ 0.496 (mag.)	**0.496**	**1.35E-09**	6.45E-11
**31**	8.13	1.97	16	663	300	Default	2.49	0.331 (sil.)/ 0.447 (mag.)	**0.704**	**4.97E-10**	5.78E-11
**32**	8.13	1.97	16	663	300	Default	12.47	0.331 (sil.)/ 0.496 (mag.)	**0.760**	**4.11E-10**	3.48E-11

Supplementary Table 7. MD simulations and results of silica nanopores (Φ = 8.1 nm) filled by 8 magnetite NPs (ϕ = 2.0 nm), according to different values of partial charges of silica or magnetite surfaces. In Case 33, the partial charges on magnetite surface are set to zero; in Case 34 all the partial charges of the setup are set to zero.

CASE	Geometry			Thermodynamics		Force-Field		Characteristic length	θ	D [m^2 s^{-1}]	+/- [m^2 s^{-1}]
	Φ [nm]	ϕ [nm]	N	ρ [kg m^{-3}]	T [K]	Partial Charges	ε_{Fe} [kJ mol^{-1}]	δ [nm]			
**33**	8.13	1.97	8	797	300	Default (SiO_2); Neutral (Fe_3O_4)	24.94	0.331 (sil.)/ 0.363 (mag.)	**0.519**	**1.64E-09**	4.46E-11
**34**	8.13	1.97	8	797	300	Neutral (all system)	24.94	0.330 (sil.)/ 0.363 (mag.)	**0.519**	**1.69E-09**	1.98E-10

Supplementary Table 8. MD simulations and results of a silica NP (ϕ = 2.0 nm) in a cubic box of water (L = 5.0 nm). Partial charges of silanol groups are modified according to the magnetite force-field, namely $q_{SI(H)}$ = 1.21 e, $q_{O(H)}$ = -1.61 e and q_H = 0.40 e.

CASE	Geometry				Thermodynamics		Force-Field		Characteristic length	θ	D [m^2 s^{-1}]	+/- [m^2 s^{-1}]
	Mat.	ϕ [nm]	L [nm]	N	ρ [kg m^{-3}]	T [K]	Partial Charges	ε_{Fe} [kJ mol^{-1}]	δ [nm]			
**35**	SiO_2	1.97	5	1	990	300	Fe_3O_4 partial charges on SiO_2	-	0.326	**0.054**	**2.64E-09**	3.11E-11

Supplementary Table 9. MD simulations and results of carbon nanotubes (Φ diameter, L length) solvated in triclinic water boxes with V volumes.

CASE	Geometry				Thermodynamics		Characteristic length	θ	D [m^2 s^{-1}]	+/- [m^2 s^{-1}]
	Chirality	Φ [nm]	L [nm]	V [nm^3]	ρ [kg m^{-3}]	T [K]	δ [nm]			
**36**	(5,5)	0.68	4.8	21.1	1076	300	0.286	**0.489**	**1.22E-09**	1.24E-10
**37**	(5,5)	0.68	4.8	73.6	989	300	0.286	**0.104**	**2.58E-09**	7.89E-11
**38**	(5,5)	0.68	4.8	315.6	971	300	0.286	**0.022**	**2.70E-09**	4.36E-11
**39**	(10,10)	1.4	9.8	131.9	989	300	0.368	**0.291**	**1.99E-09**	1.64E-10
**40**	(20,20)	2.7	9.8	260.1	989	300	0.369	**0.296**	**1.93E-09**	5.89E-11
**41**	(30,30)	4.1	9.8	431.4	989	300	0.369	**0.264**	**2.06E-09**	1.39E-10

Supplementary Table 10. MD simulations and results of proteins, with SAS surface accessible surface, solvated in water boxes with V volumes.

CASE	Geometry			Thermodynamics		Characteristic length	θ	D [m^2 s^{-1}]	+/- [m^2 s^{-1}]
	Protein	SAS [nm^2]	V [nm^3]	ρ [kg m^{-3}]	T [K]	δ [nm]			
**42**	B1 Immunoglobulin	36.4	23.1	934	300	0.295	**0.675**	**8.72E-10**	5.22E-11
**43**	B1 Immunoglobulin	36.4	24.0	934	300	0.295	**0.639**	**7.98E-10**	8.01E-11
**44**	B1 Immunoglobulin	36.4	25.7	903	300	0.295	**0.582**	**8.64E-10**	9.98E-11
**45**	B1 Immunoglobulin	36.4	26.0	899	300	0.295	**0.571**	**8.85E-10**	7.40E-11
**46**	B1 Immunoglobulin	36.4	347.5	989	300	0.295	**0.032**	**2.41E-09**	3.80E-11
**47**	Ubiquitin	48.2	60.0	936	300	0.309	**0.300**	**1.70E-09**	7.42E-11
**48**	Ubiquitin	48.2	111.8	976	300	0.309	**0.147**	**2.09E-09**	9.13E-11
**49**	Ubiquitin	48.2	252.2	979	300	0.309	**0.062**	**2.42E-09**	1.35E-11
**50**	Ubiquitin	48.2	347.5	989	300	0.309	**0.044**	**2.39E-09**	9.31E-11
**51**	Ubiquitin	48.2	816.6	993	300	0.309	**0.019**	**2.57E-09**	5.14E-11
**52**	Green Fluorescence Protein	108.8	347.5	989	300	0.302	**0.105**	**2.23E-09**	4.06E-11
**53**	Ca^{2+}-ATPase	448.2	1214.8	989	300	0.309	**0.129**	**2.32E-09**	3.93E-11
**54**	Glucokinase	198.2	579.9	989	300	0.302	**0.117**	**2.34E-09**	3.20E-11
**55**	Leptin	68.9	347.4	989	300	0.306	**0.064**	**2.44E-09**	1.73E-11
**56**	Myoglobin	90.7	347.4	989	300	0.295	**0.082**	**2.40E-09**	6.84E-11
**57**	Lysozyme	67.4	347.4	989	300	0.315	**0.065**	**2.30E-09**	3.86E-11

Supplementary Table 11. MD simulations and results of SPC/E water in a cubic box of water (L = 1.9 nm).

CASE	Geometry		Thermodynamics		θ	D [m^2 s^{-1}]	+/- [m^2 s^{-1}]
	Mat.	L [nm]	ρ [kg m^{-3}]	T [K]			
**58**	Water	1.9	1004	300	**0**	**2.60E-9**	6.26E-11

Supplementary Table 12. Parameters for bonded interactions within silica. [1]

Interaction	k_{ij}^{b} [kJ mol^{-1} nm^{-2}]	k_{ijk}^{ϑ} [kJ mol^{-1} rad^{-2}]	r_{ij}^{0} [nm]	ϑ_{ijk}^{0} [deg]
Si - O	126356.8	-	0.161	-
Si(H) - O(H)	135980.0	-	0.161	-
H - O(H)	236814.4	-	0.096	-
O - Si - O	-	125.5	-	109.8
O - Si(H) - O(H)	-	133.9	-	109.8
O(H) - Si(H) - O(H)	-	125.5	-	109.8
Si - O - Si	-	142.3	-	109.8
Si - O(H) - Si(H)	-	142.3	-	143.6
Si(H) - O(H) - Si(H)	-	142.3	-	143.6
Si(H) - O(H) - H	-	142.3	-	128.8

Supplementary Table 13. Parameters of nonbonded interactions within silica. [2]

Atom	σ_{ii} [nm]	ε_{ii} [kJ mol^{-1}]	q_i [e]
Si(H)	0.32	0.65	0.31
O(H)	0.38	0.53	-0.71
Si	0.32	0.65	0.00
O	0.38	0.53	0.00
H	0.00	0.00	0.40

Supplementary Table 14. Parameters of nonbonded interactions within magnetite. [3,4]

Atom	σ_{ii} [nm]	ε_{ii} [kJ mol^{-1}]	q_i [e]
Fe^{2+} (H)	0.43	24.94	1.21
Fe^{3+} (H)	0.43	24.94	0.73
O (H,2)	0.38	0.53	-1.61
O (H,3)	0.38	0.53	-1.13
Fe^{2+}	0.43	24.94	0.00
Fe^{3+}	0.43	24.94	0.00
O	0.38	0.53	0.00
H	0.00	0.00	0.40

Supplementary Notes

Supplementary Note 1. Simulated Molecular Dynamics setups and results. In order to investigate the influence of geometry and material properties, the self-diffusion coefficient D of water molecules is computed for 58 different configurations. In **Supplementary Table 1** D is evaluated according to different diameters of silica nanopores; in **Supplementary Table 4** the D obtained around silica or magnetite NPs at different concentrations (i.e. L size of the water box) is shown; in **Supplementary Tables 2** and **3** different configurations of nanopores filled by NPs are simulated. In **Supplementary Table 5** the effect of water density on D is explored, whereas in **Supplementary Table 6-8** the effect of force-fields (i.e. strength of attractive nonbonded forces on solid surface) is evaluated. In **Supplementary Tables 9** and **10** the D obtained around CNTs or proteins at different concentrations (i.e. L size of the water box) is shown, respectively. Finally, in **Supplementary Table 11** D of bulk water is computed.

Notice that in Case 10 and Case 12 NPs are initially placed randomly within the silica nanopore, whereas in all other cases NPs are initially placed on the surface of silica nanopore, where they tend to adsorb during the remaining time of computation.

Finally, the influence of temperature on D has been explored, considering Case 7 as a reference and changing the temperature of the system to 350 K and 280 K, the measured D are $3.37 \pm 0.14 \times 10^{-9}$ m^2s^{-1} and $0.86 \pm 0.03 \times 10^{-9}$ m^2s^{-1}, which implies a linear trend between D and temperature in accordance with the Einstein relation. However, the anomalous dynamics of nanoconfined water at different temperatures [5,6] and the supercooled regime at low temperatures [7,8] are beyond the aims of this work and will require further investigations.

Supplementary Note 2. Convergence of Molecular Dynamics simulations. First, the analyzed systems are equilibrated in order to measure the self-diffusion coefficient D of water molecules at steady state conditions (in **Supplementary Figures 12–14** the systems' energies of a few equilibrated cases are shown).

Hence, D is determined following the classical relationship of Einstein and computing the mean square displacement (MSD) as [9,10] $MSD = \lim_{t\to\infty}\langle\|\vec{r}_i(t) - \vec{r}_i(0)\|^2\rangle_{i\in water} = 6Dt$ (in **Supplementary Figures 15–17** a few cases are shown), where the position vector $\vec{r}_i$ refers to the center of mass of the water molecule i at the generic time t, and 0 refers to the initial configuration of the system. Note that the MSD trend is linear after few picoseconds ($MSD \propto t^\alpha$ with $\alpha = 1$), which denotes classical diffusion by Brownian motion. **Supplementary Figure 18** depicts a double logarithmic plot of MSD versus time, where three different segments can be clearer distinguished, indicating three different diffusive regimes: (i) a ballistic region ($\alpha \cong 2$), [11] within a sub-picosecond domain; (ii) a cage breaking sub-diffusion regime ($\alpha < 1$), [12] within 1–50 ps according to the different configurations; (iii) the Brownian diffusion regime ($\alpha = 1$), where the D of water is evaluated. Note that in cases where water molecules are highly confined (e.g. Case 1 in **Supplementary Figure 15**), the sub-diffusion regime can last tens of picoseconds, because the proximity of most of the water to a surface significantly affects the cage-breaking needed by the diffusing molecules for escaping their own hydration shell. [12,13]

Finally, the convergence of D in 1 ns runs is verified by evaluating it every 200 ps and by verifying that D tends to an asymptotic value, as shown in **Supplementary Figures 19–21**. For the sake of completeness, a few setups are extended up to 2 ns, in order to further assess the stability of D in longer runs. In particular, two silica nanopores (**Supplementary Figures 22, 23**), two silica/magnetite nanoparticles (**Supplementary Figures 24, 25**) and two silica

nanopores filled by magnetite nanoparticles (**Supplementary Figures 26, 27**) are considered. Note that error bars are obtained by fitting MSD in different time intervals, whereas the dotted line indicates the value of water self-diffusion coefficient evaluated in the 600-1000 ps interval. The extended simulations confirm that D converges to a steady state value after approximately 0.6 ns, then it fluctuates (less than ±10%) around the equilibrium value.

After a few hundreds of picoseconds, the stability of the simulated protein structures is assured by the convergence of: (i) systems' energies (e.g. **Supplementary Figure 28**); (ii) root mean square deviation of the structures with respect to the crystallographic ones (averagely below 0.3 nm, e.g. **Supplementary Figure 29a**); (iii) radius of gyration of the protein (e.g. **Supplementary Figure 29b**).

SUPPLEMENTARY DISCUSSION

Water density in nanoconfined geometries

Water molecules in a close proximity of solid surfaces are subject to van der Waals and Coulomb interactions, which interfere with their dynamics. This induces a layering of water molecules near the surface. Among these layers, only the first and partially the second layer of water (i.e. within 0.3 – 1.0 nm from the solid surface [14]) show appreciable different physical properties as compared to bulk water. [15,16]

In **Supplementary Figure 1**, the density ρ of water within a silica nanopore, around a magnetite NP and in a silica nanopore with magnetite NPs are shown. In all simulated cases, the density profile is clearly flat over 0.5–1.0 nm from the solid surface, according to the different geometries and force-fields used. Layering effects of water can be noticed at the solid interface, where water is organized in a mono, double or more layer structure.

The first layer of water around the surface of a silica nanopore ($\Phi = 2$ nm) shows a peak of 1.2 ρ/ρ_B (ρ_B= 1000 kg m^{-3} is the bulk density at 300 K). A second layer is barely observable at r = 0.4 nm from the center of the pore. Moreover, local peaks of 2.5-3.0 $\rho/\bar{\rho}$ can be noticed in the narrower cavities at the pore surface (**Supplementary Figure 1 a, d)**. In **Supplementary Figure 2** the water densities within nanopores with different diameters are compared. We can notice that a higher diameter implies a greater region of bulk water, because the interface region (i.e. the first layers of water) is still relegated to the first 0.5–1.0 nm from the solid surface. Moreover, the peak intensity of the first layer of water is almost the same in all cases, i.e. 1.2 ρ/ρ_B.

For the magnetite NP ($\phi = 2.0$ nm), the first layer of water shows a clear peak of 1.5 ρ/ρ_B, and a second layer of water is also visible with a peak of 1.1 ρ/ρ_B (**Supplementary Figure 1 b,**

e). The higher peak in the density profile for magnetite is determined by the selection of a stronger force-field.

The density distribution of water within a system composed by a silica nanopore ($\Phi = 8.1$ nm) filled by 4 magnetite NPs ($\phi = 2.0$ nm) is then considered. In **Supplementary Figure 1 c**, local peaks of 2.5-3.0 ρ/ρ_B can be noticed around the NPs and in the narrower cavities of the nanopore, with a synergistic effect in the interstices between NPs and nanopore surface; whereas in **Supplementary Figure 1 f** the radial distribution of ρ/ρ_B shows an expected drop in water density where the NPs are located. However, ρ/ρ_B peaks are still noticeable around NPs and nanopore surfaces, even though radially averaged with the rest of bulk water within the nanopore.

Finally, the density distribution of water around (and within) a (20,20) CNT is considered. In **Supplementary Figure 3**, local peaks of 2.3 ρ/ρ_B can be noticed in the proximity of CNT surface. Notice that the apparently asymmetric distribution of water densification around the CNT is given by the slight tumbling of the geometry around the Y axis during the simulation.

Note that the hydrophilic liquid-solid interaction within the first layers of adsorbed water induces a strong distortion of the hydrogen bond network, which in turn significantly affects the dynamics of confined water. [17,18]

Of course, the level of hydration determines the effective pressure, namely the sum of the bulk pressure and the spreading pressure, [19] inside the low mobility region due to nanoconfinement and consequently the thermodynamic state of water in the low mobility region. Eventually the effective pressure could determine a switch from a low-density liquid to a high-density liquid. [20] Our observations here refer to the mobility of nanoconfined water, which are not greatly affected by the actual local density.

Alternative definitions of θ dimensionless scaling parameter

In this subsection, two alternative definitions of the scaling parameter θ are discussed in order to further support the one discussed in the main text. The first one is based on the notion of interfacial work; while the second makes an attempt to incorporate also the water density / hydration level.

θ accounting for interfacial work

Before considering the discussed procedure, a preliminary effort has been made to search for a proper scaling quantity. Inspired by other succesful works, where a scaling behaviour could be found by resorting to the notion of interfacial work, [21,22] our first attemp in searching for the scaling parameter was focused on the following argument.

Let us consider the schematics in **Figure 2b**. For an arbitrary particle p, inspired by the computation of $\delta^{(p)}$, we also define a characteristic energy $\varepsilon^{(p)}$:

$$\varepsilon^{(p)} = \frac{\sum_{i=1}^{N_a} \varepsilon_i^{(p)} S_{loc,i}}{S_{tot}}, \tag{1}$$

where $\varepsilon_i^{(p)}$ represents the well depth of the potential energy shown in **Figure 2c)**. Since the interfacial work between a particle and the solvent is proportional to the above $\varepsilon^{(p)}$, a possible guess for the scaling quantity would be:

$$\theta_e = \frac{1}{V_w} \frac{\sum_p \varepsilon^{(p)} V_{in}^{(p)}}{N_{av} k_B T}, \tag{2}$$

where N_{av} and $V_{in}^{(p)}$ are the Avogadro Number and the volume of influence of particle p, respectively. However, if θ_e is assumed as a unique independent variable for scaling the D values, a poor correlation appears, as evident in **Supplementary Figure 31**. Hence, θ_e was judged not suitable for our purpose.

θ accounting for water density

The suggested scaling parameter θ in Equation (3) does not include the density of water within the analyzed configurations. In fact, the considered MD setups are characterized by a range of hydration levels (from 660 to 1080 kg m^{-3}), where no heterogeneous wetting and anomalous behavior due to low water filling regimes is expected. [6,23] Towards an effort of incorporating also the hydration level, the following variable θ_d was also considered as a scaling parameter:

$$\theta_d = \frac{\rho_B}{\rho}\theta, \quad (3)$$

where ρ_B is the bulk density of water (given the pressure and the temperature), ρ is the actual water density in the setup, while θ is the suggested scaling variable previously defined by Equation (3).

In **Supplementary Figure 32** and **Figure 3** we report the results obtained when the scaling variables θ_d and θ are used, respectively.

Estimate of the scaling parameter $\boldsymbol{\theta}$ for the literature data

In **Figure 3** we also included results from the literature. By referring to the work of Milischuk et al., [24] we have computed the corresponding scaling parameter for the reported silica nanopores (with diameters 2, 3 and 4 nm) as follows. Considering the curvature effect, the volume of influence within a pore can be estimated as: $V_{in} = \delta S_{SAS}(1 - \delta/d_{SAS})$, with S_{SAS} and d_{SAS} being the solvent accessible surface area and the corresponding diameter, respectively. Hence, for those cases, $\theta = \theta' = \left[\delta S_{SAS}\left(1 - \frac{\delta}{d_{SAS}}\right)\right]/V_{pore}$, with V_{pore} being the volume of water within the

pore. Finally, both the axial ($D_{\parallel}$) and radial ($D_{\perp}$) diffusion coefficients are provided, hence here we report the value of: $D = \frac{1}{3}D_{\parallel} + \frac{2}{3}D_{\perp}$.

In reference [25] water confined within five single walled carbon nanotubes (CNTs) is simulated. By referring to Figure 5 of reference [25], again both the axial ($D_{\parallel}$) and radial ($D_{\perp}$) diffusion coefficients at T=298 K are provided, hence for each configuration we are here interested in the value of: $D = \frac{1}{3}D_{\parallel} + \frac{2}{3}D_{\perp}$. Let d_{CNT} and σ_{CO} be the nominal tube diameter and the minimal distance between a carbon atom and the water molecule, respectively. Owing to the high curvature of those cases, the volume of influence has been more accurately computed as: $V_{in} = \pi \left[\left(\frac{d_{SAS}}{2} \right)^2 - \left(\frac{d_{SAS}}{2} - \delta \right)^2 \right] L$, where L is the tube length while $d_{SAS} = d_{CNT} - \sigma_{CO}$. On the other hand, the total volume of water reads: $V_w = \pi \left(\frac{d_{SAS}}{2} \right)^2 L$, therefore the scaling parameter (for small CNTs) takes the simple form: $\theta = \theta' = V_{in}/V_w = 4 \left[\frac{\delta}{d_{SAS}} - \left(\frac{\delta}{d_{SAS}} \right)^2 \right]$. In **Figure 3** we reported cases with $d_{CNT} = 1.08\, nm, d_{CNT} = 1.36\, nm, d_{CNT} = 1.62\, nm, d_{CNT} = 1.9\, nm$ and $d_{CNT} = 2.17\, nm$, corresponding to chirality (8,8), (10,10), (12,12), (14,14) and (16,16), respectively, with $\sigma_{CO} = 0.33\, nm$ (consistently with the value reported in reference [25]).

Finally, we considered the results in Figure 2 of reference [26], where the radial profile of the water diffusion coefficient around myoglobin has been reported as a function: $D = D(r)$ (see also reference [27]). Clearly, at a fixed distance from the protein $r = \bar{r}$, a value for the scaling parameter θ can be computed as: $\theta = \theta' = \frac{\delta S_{SAS}}{V_w}$, while the corresponding (averaged) value of the diffusion coefficient reads:

$$\overline{D} = \frac{\int_{r_0}^{\bar{r}} D(r) 4\pi r^2 dr}{\int_{r_0}^{\bar{r}} 4\pi r^2 dr}, \tag{4}$$

with r_0 denoting the location of the water molecules nearest to the protein. In **Figure 3**, we report five values from the curve in Figure 2 of reference [26] corresponding to the following distances from the protein $\bar{r}$: 0.4 nm, 0.55 nm, 0.65 nm, 0.75 nm, 0.85 nm.

D-θ model: further details about thermodynamic insights

In this subsection, we interpret the scaled results reported in **Figure 3** by a model based on experimental data of nanoconfined water.

Unfortunately, despite its fundamental importance in science and technology, the physical properties of water are far from being completely understood. [20] The liquid state of water is definitively unusual because it behaves as if there exists a singular temperature toward which its thermodynamic properties diverge. [20] Hence, the efforts of scientists from many disciplines to seek a coherent explanation for this unusual behavior make water one of the most important open questions in science today. [20]

Very briefly, one could say that water in the presence of heterogeneities (e.g. in contact with foreign substances or surfaces) freezes at atmospheric pressure at the melting temperature $T_M \approx$ 273.15 K. However, pure bulk water can be supercooled below its melting temperature down to the homogenous nucleation temperature $T_H \approx 235$ K, below which it inevitably crystallizes. In the range between the melting temperature and the homogenous nucleation temperature, liquid water exists in a supercooled metastable state, which is characterized by anomalies in its thermodynamic properties. [20] There is a growing interest in the prediction of properties of supercooled water. In particular, in applied atmospheric science, it is commonly accepted that the uncertainties in numerical weather prediction and climate models are mainly caused by poor understanding of properties of water in tropospheric and stratospheric clouds, where liquid water

can exist in a deeply supercooled state. [28] In particular, thermodynamic properties of supercooled water diverge toward a singular temperature $T_S = 228$ K. [20] In 1992, Poole *et al.* suggested that the anomalous properties of supercooled water may be caused by a critical point that terminates a line of liquid–liquid separation of lower-density and higher-density water. [29] This assumption was provocative because liquid water states under normal conditions are inaccessible below the homogenous nucleation temperature. For this reason, the range of temperatures between T_H and $T_X \approx 150$ K, where low density amorphous ice melts into a highly viscous fluid, is usually named *no-man's-land* for liquid water. On the other hand, the assumption by Poole *et al.* led to the notion of virtual critical point of liquid–liquid coexistence (LLCP), which is still used nowadays to formulate accurate equations of states for supercooled water. [28] It is worth to point out that some insights into phases of liquid water can be obtained by alternative interpretations, for example based on the order-disorder transition scenario. [30]

Actually, there are at least two methods to explore the *no-man's-land* for liquid water, namely the liquid states at temperature below T_H. One possibility is to use molecular solutions rich in water. [30] Another one, which is receiving a growing attention because of the practical implications in nanotechnology, is to study water in nanoscopic confinement. Water near surfaces often does not crystallize upon cooling, but only recently have the properties of such water been measured. [30] For example, when confined in nanopores made of porous hydrophilic silica glass (Vycor), water does not crystallize and can be supercooled well below T_H. [20] Nanoconfinement seems to confirm the assumption of a second critical point scenario, because it clearly reveals the existence of a peak in the specific heat capacity (Widom line) and a dynamic crossover in the self-diffusion properties of water, at $T_L \approx 225$ K, between the two dynamical behaviors known as fragile and strong, which is a consequence of a change in the hydrogen bond

structure of liquid water. [20,31] Clearly this situation resembles the pseudo-critical temperature in supercritical fluids. [32] Nanoconfinement seems to damp out the peaks in heat capacity, rather than to exaggerate, the behavior in real water [30] (see, for example, Figure 1 in reference [31]). Some authors attribute this smoothing to the confinement length scale effects. [33] Alternatively, one could interpret the results by imaging that nanoconfinement acts as a pressure. [30] This would be consistent with the concept of spreading pressure, which is popular in the thermodynamics of adsorption and, more generally, in the non-equilibrium thermodynamics of heterogeneous systems. [19]

Coming back to our scaled results reported in **Figure 3**, it is reasonable from the previous discussion to describe water in the region with lower molecular mobility (C) as supercooled liquid water with strong nanoconfinement (characteristic length δ is smaller than 0.6 nm). The thermodynamic state depends on the confinement length scale δ [33] but only few experimental data are available. For example, let us consider the experimental data about the specific heat capacity of ordinary water confined in silica nanopores as a function of the system temperature (**Figure 4a**). [31] This thermodynamic property depends on the diameter of the nanopore. However, for very small nanopores (<1.7 nm), the heat capacity shows no meaningful peak, which suggests that no ice is present (effectively too far from the virtual LLCP because of the surface spreading pressure). In case of strong nanoconfinement, the heat capacity c_p seems to move gradually from a temperature-independent plateau, which starts at room temperature, to a linear function of the temperature, which is typical for ice, at very low temperature (<100 K).

In the following, we base our analysis on the experimental curve corresponding to the system under the strongest nanoconfinement (nanopore with diameter 1.7 nm), and assume that such a results is the most representative of a generic configuration with $\theta \approx 1$.

By numerical integration, the function $\Delta h = f(T) = -\int_T^{T_0} c_p dT$ can be evaluated with $f(T_0) = 0$, and $T_0 = 300$ K being the room temperature (**Figure 4b**). [31]

For any reduction of the system energy $\Delta h \leq 0$, the previous function allows one to estimate the corresponding system temperature $T = f^{-1}(\Delta h)$. The latter temperature can be used as the input variable for computing the self-diffusion coefficient D of supercooled nanoconfined water according to reference [20], (where nanopores of 1.4 nm and 1.8 nm were used to experimentally investigate on the value of D_C under strong confinement. See **Figure 4c**). In other words, from reference [20] we can easily extract the function: $\frac{D_C}{D_B} = g(T)$ which combined with the above $T = f^{-1}(\Delta h)$ it yields $D_C/D_B = g(\Delta h)$, as shown in **Figure 4d**.

The plot in **Figure 4d** is the fundamental result used to interpret our scaled results. The volume occupied by the water molecules is partitioned in two regions, the one with higher molecular mobility (B), coinciding with the bulk water, while the other with lower molecular mobility (C), including the confined molecules in the water boundary layer at the liquid-solid interface. Let h_B be the molar enthalpy of the bulk region and $h_C \leq h_B$ the molar enthalpy in the adsorbed region. Clearly, $\Delta h = h_C - h_B \leq 0$ indicates the energy of adsorption. [19] The potential well Δh is the average potential in the confined region defined by δ, which is comparable with the water molecule size. This means that only 1-2 water layers fit in the potential well and hence $\Delta h \approx -\varepsilon$, where $-\varepsilon$ is the (total) potential minimum, is a good approximation. In this way, the self-diffusion of water in the nanoconfined region defined by δ can be estimated by the strength of the surface potential, namely

$$D_C = D_B\, g(-\varepsilon). \tag{4}$$

The next step consists in computing the average diffusivity of water D over the whole volume, which is partitioned in bulk region B with volume V_B where water molecules have diffusivity D_B

and adsorbed region C with volume V_C where molecules have diffusivity D_C. Let us recall the definition of diffusivity, namely

$$D = \lim_{t\to\infty} \frac{1}{6t} \langle \|\vec{r}_i(t) - \vec{r}_i(0)\|^2 \rangle_{i\in V_B+V_C} = \frac{1}{N_B+N_C} \lim_{t\to\infty} \frac{1}{6t} \sum_i^{N_B} \|\vec{r}_i(t) - \vec{r}_i(0)\|^2 + \frac{1}{N_B+N_C} \lim_{t\to\infty} \frac{1}{6t} \sum_i^{N_C} \|\vec{r}_i(t) - \vec{r}_i(0)\|^2 = \left(1 - \frac{N_C}{N_B+N_C}\right) D_B + \frac{N_C}{N_B+N_C} D_C. \quad (5)$$

with N_C and N_B being the numbers of water molecule in the two regions. The previous formula is simply a mixing rule based on the molar concentrations. Taking into account that $N_C = \rho_C V_C / m$ where ρ_C is the water density in the adsorbed region and m is the mass of one water molecule, the previous expression becomes

$$D = \left[1 - \frac{\rho_C \theta}{\rho_C \theta + \rho_B (1-\theta)}\right] D_B + \frac{\rho_C \theta}{\rho_C \theta + \rho_B (1-\theta)} D_C. \quad (6)$$

Taking into account Supplementary Equation (4) yields

$$\frac{D(\theta,\varepsilon)}{D_B} = 1 + \frac{\rho_C \theta}{\rho_C \theta + \rho_B (1-\theta)} [g(-\varepsilon) - 1]. \quad (7)$$

The previous equation is the fundamental one of the proposed model. Here, the average diffusivity for the considered volume depends on a geometrical parameter θ and an energetic parameter ε. For the considered setups, some simplifications are possible. First of all, the following function: $q = \frac{\rho_C \theta}{\rho_C \theta + \rho_B (1-\theta)} = \frac{1+\iota}{\iota + 1/\theta}$ (with $\rho_C = (1+\iota)\rho_B$) can be safely approximated as $q = \theta$ for the values of ρ_C of interest. In fact, here a maximum value for ι was observed to be: $\iota = 0.1 - 0.2$ (e.g. based on the density profile shown in **Supplementary Figure 1e)** for an SPIO particle), where the (average) value for ρ_C was computed by $\frac{\int_{r_0}^{r_0+\delta} \rho_C 4\pi r^2 dr}{\int_{r_0}^{r_0+\delta} 4\pi r^2 dr}$. In **Supplementary Figure 4** the exact function $q = \frac{1+\iota}{\iota + 1/\theta}$ for two quite large values of ι ($\iota = 0.25, \iota = 0.5$) are shown. The negligible deviations between the above and the approximated function $q = \theta$ (even in such conservative cases) support the above simplifying assumption.

Moreover, as it is clearly shown in **Figure 4d**, the considered surfaces are characterized by small values for $g(-\varepsilon)$. Hence, under the two previous simplifying assumptions ($g(-\varepsilon) \approx 0$ and $q \approx \theta$), the Supplementary Eqaution (7) reduces to

$$D(\theta) = D_B(1 - \theta). \tag{8}$$

which well matches both our numerical simulations and data from the literature (see **Figure 3**) with an excellent coefficient of determination ($R^2 = 0.93$), and most importantly without the need of introducing any empirical factors.

Outer sphere theory

Transversal relaxivity r_2 of T_2 contrast agents (CAs) such as SPIO nanoparticles is given by

$$r_2 = \frac{1/T_2 - 1/T_{0,w}}{M_{Fe}}, \tag{9}$$

being $T_{0,w}$ the diamagnetic contribution ($T_{0,w} \cong 2.8$ s), and M_{Fe} the iron concentration in solution, in mM. T_2 is the transverse relaxation time of NP, which can be modeled by the quantum-mechanical outer-sphere theory [34-36]

$$\frac{1}{T_2} = \frac{4}{9} \upsilon \tau_D (\Delta\omega_r)^2 \tag{10}$$

when the CA fulfills the condition

$$\tau_D < 1/\Delta\omega_r, \tag{11}$$

which indicates a motional averaging regime (MAR). In a MAR regime water protons, because of their diffusion, experience changing magnetic fields, which are effectively time-averaged. Supplementary Equation (11) is generally fulfilled in case of ultrasmall NPs (i.e. diameters less than 10 nm). [37] υ is the volume fraction of NPs, which can be expressed as

$$\upsilon = \frac{4}{3}\pi a^3 N_{av} M_{SPIO} \times 10^{-3} \quad (12)$$

where $a = r + L$ is the effective radius of NP (magnetic core with r radius; coating layer with L thickness), N_{av} is the Avogadro number and M_{SPIO} is the molarity of SPIOs, in mM. τ_D is the time needed for water to diffuse across the NP surface; whereas $1/\Delta\omega_r$ is the angular phase shift induced by the NP at the equator line on its surface. Note that it has been proven that outer sphere theory accurately predicts r_2 of SPIOs and other superparamagnetic contrast agents. [36,37]

Different definitions of τ_D and $\Delta\omega_r$ have been suggested in literature, [36,38] and some of their physical parameters can be subject to multiple interpretations. If the model suggested by Tong *et al.* is considered, [36]

$$\tau_D = r^2/D \quad (13)$$

$$(\Delta\omega_r)^2 = \frac{4}{5}\gamma^2\mu_c^2/a^6 \quad (14)$$

where D is self-diffusion coefficient of water molecules around the CA, $\gamma = 2.675 \times 10^8 \mathrm{s}^{-1}\mathrm{T}^{-1}$ is the proton gyromagnetic ratio and μ_c is the Curie moment of SPIOs. In Supplementary Equation (13) the choice of r is justified by arguing that the coating is not completely impermeable, and water molecules can diffuse until the crystal core, especially in the case of shorter and less dense coatings. At high magnetic field, it is possible to approximate $\mu_c \cong \frac{4}{3}\pi r^3 M_s$, where M_s is the saturation magnetization of NP. [39] Therefore, Supplementary Equation (10) may also be recast as:

$$\frac{1}{T_2} = \frac{4}{9}\left(\frac{4}{3}\pi a^3 N_{av} M_{SPIO} \times 10^{-3}\right)\left(\frac{r^2}{D}\right)\left[\frac{\frac{4}{5}\gamma^2\left(\frac{4}{3}\pi r^3 M_s\right)^2}{a^6}\right], \quad (15)$$

which can be finally simplified as

$$\frac{1}{T_2} = \frac{256}{405}\pi^2\gamma^2 \upsilon^* M_S^2 \frac{r^5}{Da^3} \qquad (16)$$

by introducing the volume fraction of the magnetic crystal core $\upsilon^* = \frac{4}{3}\pi r^3 N_{av} M_{SPIO} \times 10^{-3}$.

Supplementary Methods

Molecular Dynamics simulations of nanopores and nanoparticles

A first set of geometries analyzed by MD simulations are cylindrical nanopores and spherical nanoparticles (NPs). Nanopores are made out silica; NPs are made out magnetite or silica.

First, a unit cell of alpha-quartz (SiO_2) [40] (leftmost picture of **Supplementary Figure 5**) is considered. The latter unit cell is replicated along all Cartesian axes, such that a fully periodic brick with size 11.30 x 11.06 x 4.32 nm^3 is constructed (middle picture in **Supplementary Figure 5**). We consider a computational domain with smaller dimensions compared to typical experiments, [41] for reducing the computational demand. However, this is not a restricting hypothesis as proved by the good scalability of the results in terms of the presented parameter θ. Subsequently, a nanopore in the above brick is obtained by removing all atoms whose distance from the brick center is smaller than a fixed length (i.e. nanopore radius). For the sake of simplicity, since our main concern is to investigate on the trend of the self-diffusion coefficient of water molecules at different SPIOs concentrations, here amorphization of silica is neglected.

Upon the creation of the pore (see the rightmost picture in **Supplementary Figure 5**), all silicon atoms located along the "cut surface" with only one bonded oxygen atom are removed. In addition, one hydrogen atom is attached to all oxygen atoms that are missing one bond with silicon (surface oxygen). This is achieved by imposing that the angle formed by silicon, oxygen and hydrogen is 128.8 degrees (elevation), [42] with a random azimuth angle (see **Supplementary Figure 7a**).

A unit cell of magnetite (Fe_3O_4) [43] (leftmost picture of **Supplementary Figure 6**) is then considered. The latter unit cell is replicated along all Cartesian axes, such that a fully periodic brick with desired size is constructed (here 3.0 x 3.0 x 3.0 nm^3, see middle picture in

Supplementary Figure 6). Finally, a spherical particle (2.0 nm diameter in this case) is obtained by retaining only atoms within a fixed distance from the brick center (radius), as shown in the rightmost picture of **Supplementary Figure 6**.

After the above cut, the NP surface is treated following the sub-steps: 1) Iron atoms Fe^{2+} and Fe^{3+} are removed when they have less than 4 and 6 bonds, respectively; 2) a bonded hydrogen atom is attached to all oxygen atoms with only one bond at the magnetite surface. The Fe-O-H angle can be estimated between 125° (DFT, quantum) and 128° (SPASIBA, empirical force field) for Fe^{2+}; while this Fe-O-H angle can be estimated between 126° (DFT, quantum) and 130° (SPASIBA, empirical force field) for Fe^{3+}. [44] In our simulations, we found that the numerical results are not very sensitive to this angle and hence we used the same angle of the silanol group (129°) for the sake of simplicity in the geometry preparation, with a random azimuth angle (see **Supplementary Figure 7b**). Such an approach of modeling the surface aims at mimicking real SPIO particles without coating, [45] and it represents a simple technique already validated in other works for iron nanoparticles in aqueous environment. [46] On the other hand, here the effect due to complex coatings will be indirectly taken into account by a sensitivity analysis of the nonbonded interactions (Lennard-Jones and partial charges parameterizations).

Towards the end of minimizing the effects due to the residual electrical dipole induced by the charges located at the NPs surface, in all the studied setups NPs are inserted in pairs within the nanopore, where the first particle (which undergoes a random rigid rotation on each Cartesian axis) is initially opposed to its mirror image with respect to the midpoint of the line segment connecting the centers of the two particles (see **Supplementary Figure 8**). In the initial configuration of our simulations, all NPs pairs have the latter segment parallel to the pore axis.

Molecular Dynamics simulations of proteins

The second set of geometries analyzed by MD simulations aims to study the effect of different proteins on the surrounding water molecules. Proteins are biological macromolecules made of one or more chains of amino acid, performing a vast variety of functions within living organisms. Amino acids show hydrophilic or hydrophobic character according to the structure and polarity of their residues. As a matter of facts, proteins may modify their structure and thus their functionality according to the dynamics of surrounding water environment. [47,48] Dense protein products, such as pharmaceutical preparations or high protein nutrition bars, involve ongoing challenges for controlling the viscosity of the water-protein system and for reducing protein self-association phenomena; [49] enzymatic activity, molecular recognition and folding process of proteins is strongly influenced by surrounding water mobility and hydration; [50] fluctuations between different conformational sub-states, which are determined by local temperature and minima of potential energy surface and are related to the biological function of the proteins, [51] are influenced by the hydrogen bond creation/destruction at the solvent-protein interface and thus by surrounding water dynamics. [52-54]

Numerous experimental and theoretical studies have already demonstrated that the water molecules in the proximity of a protein surface are subjected to confined dynamics, i.e. a general decrease in the self-diffusion coefficient respect to the bulk value and a substantial modification of the actual solvent structure. [55-57] Water diffusion has been studied both locally (i.e. by considering the water diffusion in the proximity of spatial uniform sub-regions of proteins) and globally (i.e. by evaluating the spatially averaged water diffusion as a function of the distance from protein surface). [50] However, a general modelling and broad physical understanding of the water mobility modification in the vicinity of any protein is still a subject of investigation,

mainly because of the variety of physicochemical properties (hydrophilic vs. hydrophobic) of the few tens of amino acids or functional groups being the building blocks of millions of known proteins.

Hence, a wide range of solvated proteins is considered, in order to systematically study their effect on the surrounding water dynamics. First, a simple B1-Immunoglobulin binding domain of streptococcal Protein G (1PGB entry in the Protein Data Bank [58]), which is involved in the bonding process between many infectious bacteria and host immunoglobulins and currently used in many biomedical applications, is considered (**Supplementary Figure 9**). B1-Immunoglobulin binding domain is solvated in SPC/E water boxes with different sizes, ranging from 348 nm^3 (11253 water molecules) to 23 nm^3 (496 water molecules), but similar water density (≅ 1000 kg m^{-3}).

Second, ubiquitin (1UBQ [59]), which is a small and globular regulatory protein found in almost all tissues of eukaryotic organisms, is considered (**Supplementary Figure 10a**). Ubiquitin is again solvated in SPC/E water boxes with different sizes, ranging from 817 nm^3 (26758 water molecules) to 60 nm^3 (1554 water molecules), but similar water density (≅ 1000 kg m^{-3}).

Finally, six more proteins, with a vast array of geometries, sizes and functions within living organisms, are also solvated at similar water density (≅ 1000 kg m^{-3}) and studied by MD simulations: Glucokinase (1V4S [60], **Supplementary Figure 10b**), which is an enzyme involved in the regulation of carbohydrate metabolism by acting as a glucose sensor, solvated in a 580 nm^3 SPC/E water box (16864 water molecules); Green fluorescence protein (1QXT [61], **Supplementary Figure 10c**), which is a fluorescent protein of many marine organisms widely used in fluorescence microscopy technique, solvated in a 348 nm^3 SPC/E water box (10353 water molecules); Leptin (1AX8 [62], **Supplementary Figure 10d**), which is a human hormone

regulating many metabolism-related behaviors such as appetite and hunger, solvated in a 348 nm^3 SPC/E water box (10878 water molecules); Lysozyme (1AKI [63], **Supplementary Figure 10e**), which is an enzyme part of the innate immune system, solvated in a 348 nm^3 SPC/E water box (10882 water molecules); Myoglobin (1MBS [64], **Supplementary Figure 10f**), which is found in the muscle tissue of vertebrates for oxygen transport, solvated in a 348 nm^3 SPC/E water box (10743 water molecules); Ca^{2+}-ATPase (1KJU [65], **Supplementary Figure 10g**), which is a P-type ion pump for transporting Ca^{2+} across the cellular membrane, solvated in a 1215 nm^3 SPC/E water box (35426 water molecules). The simulated conformation of Ca^{2+}-ATPase corresponds to the Ca^{2+}-free (E2) state. As noticeable in **Supplementary Figure 10**, the geometry of simulated proteins ranges from almost spherical (e.g. ubiquitin) to elongated (e.g. Ca^{2+}-ATPase) shapes, from small (e.g. 562 atoms of B-Immunoglobulin binding domain) to larger sizes (e.g. 9667 atoms of Ca^{2+}-ATPase); whereas the biological function spans from catalytic (e.g. Glucokinase) to hormonal (e.g. Leptin) or transport proteins (e.g. Myoglobin).

Note that crystal water molecules are removed from original PDB files, in order to fully solvate the protein by means of the GROMACS' tool *genbox*.

Molecular Dynamics simulations of carbon nanotubes (CNTs)

The last set of geometries analyzed by MD simulations aims to evaluate the diffusion of water in the proximity of strongly hydrophobic surfaces, such as carbon nanotube (CNT) ones. CNTs have been recognized as promising building blocks of novel nanostructured materials, because of their exceptional mechanical, thermal and electrical features. [66] Mechanical, thermal and electrical properties of CNTs are well documented in the literature; [66,67] in contrast, even if CNT-based porous materials may be interesting components of nanofluidic devices for biomedical applications, [68-70] the physical understanding of transport properties of fluids through or around

their pores is still incomplete, because of the difficulty in setting up adequate experimental setups. [71] Nevertheless, computational studies have already demonstrated a general reduction of water mobility both within (e.g. **Supplementary Figure 11b**) and around CNTs (e.g. **Supplementary Figure 11c**), or close to graphene surfaces, according to different pore size, surface functionalization or hydration level of these carbon structures. [71]

In this study, armchair CNTs with different chirality (i.e. diameter) are solvated in triclinic SPC/E water boxes with different volumes, in order to evaluate the geometry effect on the overall self-diffusion coefficient of water. First, a (5,5) CNT (i.e. 0.7 nm diameter) 5 nm long (**Supplementary Figure 11a**) is solvated in water boxes ranging from 15 (317 water molecules) to 316 (10023 water molecules) nm^3 (**Supplementary Figure 11c**). Then, 10 nm long (10,10) CNT (i.e. 1.4 nm diameter), (20,20) CNT (i.e. 2.7 nm diameter) and (30,30) CNT (i.e. 4.1 nm diameter) are solvated in 132 (3689 water molecules), 260 (7284 water molecules) and 431 (12351 water molecules) nm^3 SPC/E water boxes, respectively. Notice that water density is $\cong$ 1000 kg m^{-3} in all simulated CNT setups, which corresponds to fully hydrated CNT surfaces.

Molecular Dynamics force-fields

Two types of interactions are considered in the MD simulations: i) Bonded interactions, among the atoms forming nanopores, nanoparticles, CNTs or proteins; ii) nonbonded interactions, between the water molecules and the solid surfaces, described via van der Waals and Coulomb potentials.

First, bonded and nonbonded interactions of proteins are modeled using the GROMOS96 43a2 force field, [72] which has been widely used for studying water dynamics in the proximity of protein surfaces. [73,74]

Second, in the silica structure, the bonded interactions are modeled by means of two harmonic terms, adopted to describe the silicon-oxygen and oxygen-hydrogen interactions. That is, a bond stretching potential between two bonded atoms i and j at a distance r_{ij} (around the equilibrium distance r_{ij}^0), and a bending angle potential between the two pairs of bonded atoms (i, j) and (j, k) (around the equilibrium angle ϑ_{ijk}^0) are considered as follows:

$$U_{BOND}(r_{ij}, \vartheta_{ijk}) = \frac{1}{2} k_{ij}^b (r_{ij} - r_{ij}^0)^2 + \frac{1}{2} k_{ijk}^\vartheta (\vartheta_{ijk} - \vartheta_{ijk}^0)^2, \tag{17}$$

with parameters reported in **Supplementary Table 12.**

Here, “Si”, “Si(H)”, “O”, “O(H)” and “H” denote bulk silicon, surface silicon (i.e. belonging to a silanol group Si-O-H), bulk oxygen, surface oxygen (i.e. belonging to a silanol group Si-O-H) and hydrogen atoms, respectively.

In the magnetite structure, sufficiently high values for the force constants in Supplementary Equation (17) are assumed for all bonded interactions (rigid particle assumption), namely k_{ij}^b = 400000 kJ mol^{-1} nm^{-2} and k_{ijk}^ϑ = 400 kJ mol^{-1} rad^{-2}. In fact, our main concern is to investigate water self-diffusion coefficient of water in nanoconfined geometries, which is affected by nonbonded interactions. Therefore, here it is not of interest to accurately describe the fast dynamics within the magnetite NPs, thus the rigid particle assumption does not affect the measurements of self-diffusion coefficient of water, as confirmed by preliminary sensitivity analyses with respect to k_{ij}^b and k_{ijk}^ϑ (results not shown).

In the CNT structure, the carbon-carbon bonded interactions are also modeled by two harmonic terms (Supplementary Equation (17)), where k_{ij}^b = 478900 kJ mol^{-1} nm^{-2}, r_{ij}^0 = 0.142 nm, k_{ijk}^ϑ = 562.2 kJ mol^{-1} rad^{-2} and ϑ_{ijk}^0 = 120°. [75-77]

Nonbonded interactions among silica atoms (consisting in both van der Waals and electrostatic interactions) are also taken into account through: (i) a Lennard-Jones term

$$U_{LJ}(r_{ij}) = 4\varepsilon_{ij}\left[\left(\frac{\sigma_{ij}}{r_{ij}}\right)^{12} - \left(\frac{\sigma_{ij}}{r_{ij}}\right)^{6}\right], \quad (18)$$

with mixed parameters consistently chosen according to the following Lorentz-Berthelot combination rules

$$\begin{cases} \sigma_{ij} = \frac{1}{2}(\sigma_{ii} + \sigma_{jj}) \\ \varepsilon_{ij} = (\varepsilon_{ii} + \varepsilon_{jj})^{1/2} \end{cases}; \quad (19)$$

(ii) a Coulomb term

$$U_C(r_{ij}) = \frac{1}{4\pi\varepsilon_0}\frac{q_i q_j}{r_{ij}} \quad (20)$$

with ε_0 being the permittivity in a vacuum, and q_i, q_j the partial charge of atoms i and j, respectively. Non-zero partial charges are assigned only to atoms at the surface of the pore and belonging to a silanol group, whereas all other atoms (bulk of silica) are considered neutral (i.e. zero partial charge q_i=0). More specifically, partial charges in a silanol group are assigned following the criterion of the overall neutral charge for the entire system, with nonbonded parameters for silica reported in **Supplementary Table 13**.

Similarly to silica, partial charges are assigned only to atoms at the magnetite surface and belonging to the Fe-O-H groups, whereas zero partial charges (i.e. q_i=0) are imposed at all other bulk atoms. Moreover, partial charges are assigned within the Fe-O-H groups in order to ensure neutrality of the whole system. Both the adopted parameterization for Lennard-Jones potentials and the partial charges are reported in **Supplementary Table 14**.

Here, “Fe^{2+}”, “Fe^{3+}”, “O” and “H” denote bulk iron(II), bulk iron(III), bulk oxygen, surface oxygen and hydrogen atoms, respectively; whereas “Fe^{2+}(H)” and “O(H,2)” are atoms belonging to Fe^{2+}-O-H surface groups, “Fe^{3+}(H)” and “O(H,3)” are atoms belonging to Fe^{3+}-O-H surface groups. For the sake of completeness, Lennard-Jones parameters and partial charge values have been further artificially changed in some MD setups, in order to perform a sensitivity analysis of forcefields on D.

Nonbonded interactions between CNTs and water molecules are modeled by Lennard-Jones potential (Supplementary Equations 18, 19), where carbon atoms are characterized by $\sigma_{CC} =$ 0.36 nm and $\varepsilon_{CC} = 0.29$ kJ mol^{-1}. [75-77] All nanotubes’ carbon atoms are considered as neutral, because of the strong hydrophobicity of pristine CNTs.

Finally, Lennard-Jones potentials are treated with a twin-range cut-off and 1.5 nm cut-off distance, whereas a Particle-Mesh Ewald (PME) [9] with 1.5 nm real-space cutoff, a 0.12 nm reciprocal space gridding, and splines of order 4 with 10^{-5} tolerance is used for electrostatic interactions.

Algorithm for automatic evaluation of θ

By referring to the flow-chart in **Supplementary Figure 30**, we report the main steps involved in the computation of the scaling parameter θ. For the sake of completeness and without loss of generality, a few GROMACS commands are also given as example. We note in fact that, the described procedure can be properly rearranged by a well educated user of other MD software packages.

1. Download or generate the dry geometry of interest (i.e. *.pdb file);
2. Chose a suitable force field (e.g. GROMOS);

3. Create the topology file;

   pdb2gmx -f geometry.pdb -o geometry_0.gro -p topology.top -i restraints.itp -water spce

   editconf -f geometry_0.gro -o geometry.gro -bt triclinic -d 2 -c

   or

   editconf -f geometry.pdb -o geometry.gro -bt triclinic -d 2 -c

   g_x2top -f geometry.gro -o topology.top -ff forcefield -noparam -pbc

4. Do energy minimization of the particle in vacuum (not always necessary);

   grompp -f em.mdp -c geometry.gro -p topology.top -o em.tpr

   mdrun -s em.tpr -o trajectory.trr -c geometry-em.gro -e em.edr

5. Solvate;

   genbox -cp geometry-em.gro -cs spc216.gro -o geometry_sol.gro -p topology.top

6. Do energy minimization of the solvated particle;

   grompp -f em_sol.mdp -c geometry_sol.gro -p topology.top -o em_sol.tpr

   mdrun -s em_sol.tpr -o trajectory_sol.trr -c geometry_sol-em.gro -e em_sol.edr

7. Use the output files of step 6 for running the command g_sas, which produces the Connolly surface (in the form of a geometry *.pdb file), local and total SAS (*.xvg file);

   g_sas -f trajectory_sol.trr -s em_sol.tpr -o sas.xvg -oa atom_sas.xvg -q connolly.pdb

8. As an input, four files are provided to the routine for the computation of the characteristic length δ.

9. Estimate the accessible volume of water V_w.

10. Compute θ.

Details of experimental procedures and materials

For the hydrophilic SPIOs loaded into the SiMPs, the samples as provided by the vendors (Sigma-Aldrich) presented several aggregates, and the following procedure was performed in order to purify the original solution and select the SPIOs with the higher stability in solution. Upon sonication (~ 15 min – Bransonic Ultrasonic Cleaner), the sample were centrifuged (6 minutes, at 12,000 rpm) and the supernatant was collected. This step was repeated twice. The resulting supernatant was used for all the experiments. For assessing the iron content in the purified solution, the colloidal suspension was digested – 2x diluted in ~1.5 ml of nitric acid (Sigma-Aldrich, 70%, purified by redistillation, ≥ 99.999% trace metals basis) and dried on a thermoplate at 110 °C. The resulting solution was re-suspended in 5 ml 2% nitric acid and filtered (0.22 μm pores size). Finally, it was analyzed via Inductively Coupled Plasma Optical Emission Spectrometer (ICP-OES) for elemental Fe content. The size of the SPIO magnetic core was measured via Transmission Electron Microscopy (JEM-2100F TEM by JEOL Ltd.). Purified samples were diluted in DI water 10 times and 10 μL of the solution was deposited onto the surface of a TEM grid (Ted Pella, Inc., Formvar/Carbon 400 mesh, Copper, approx. grid hole size: 42μm) and left to dry for 1h. The size distribution was estimated from the analysis of the TEM images considering more than 100 SPIOs (**Supplementary Figure 33**).

SiMPs were fabricated by using previously reported protocols. [78] The fabrication process consists of three major steps: Formation of porous silicon films; photolithographic patterning of particles; and Reactive Ion Etch (RIE). The porous structure was tailored by electrochemical etching while the particle sizes were precisely defined by photolithography. Since the porous structure and the particle size are controlled independently, a wide range of sizes, shapes and pore morphologies can be obtained using such an approach. In this work, particles with 1,000 nm

in diameter and 400 nm in thickness were used. These particles have a mean pore size of 40 nm and a porosity of about 60%. The fabrication process is briefly described as follows: Starting with heavily doped P-type (100) wafer with resistivity of 0.005 ohm-cm as the substrate (Silicon Quest, Inc, Santa Clara, CA), the wafer was assembled on a home-made anodizing cell with the polished surface immersing in 1:3 HF(49%):ethanol solution. An etching current of 6 mA/cm^2 was applied for 125 sec to generate 400 nm porous silicon film. Then a high electrical current with current density ~76 mA/cm^2 was applied for 8 sec to form the instable release layer. An 80 nm low-temperature oxide (LTO) was deposited on the porous silicon film in a LPCVD furnace. A standard photolithography process was used to pattern the 1,000 nm circles on the film using a contact aligner (SUSS MA6 mask aligner) and NR9-500P photoresist (Futurrex Franklin, NJ, USA). The pattern was transferred into the porous silicon film by RIE in CF4 plasma (Plasmatherm BatchTop, 15 sccm CF4, 100 mTorr, 200 W RF). After striping the LTO, the porous silicon disk arrays were released in isopropanol solution by ultrasound for 1 minute. The SEM imaging of the particles was performed using a ZEISS NEON 40 Scanning Electron Microscope (SEM) at 5kV and 3-5 mm working distance using an In-lens detector (**Supplementary Figure 34**). The volume, size and concentration particles were characterized by a Multisizer 4 Coulter Counter (Beckman Coulter).

As per the loading of SPIOs into SiMPs, the stock solutions of SPIOs as purchased (5 nm from Sigma) was first purified to select particles with higher aqueous stability. Then, SiMPs were lyophilized to dryness for 8 hours and 2×10^8 SiMPs were exposed to 100 µL of the purified SPIO solution. The resulting suspension was sonicated (30 W bath sonicator) for 1 min and centrifuged for 10 min at 4,000 rpm. The supernatant was decanted and the precipitate washed twice with 200 µL deionized water to remove any free SPIOs from the SiMP surface. The

resulting assembly was resuspended in 200 µL of deionized water and characterized using HR-TEM (JEOL 2000 FX) equipped with an energy-dispersive spectrometer (EDS) and ICP-OES (Perkin-Elmer Inductively Couples Plasma Optical Emission Spectrometer). For TEM and EDS samples were diluted with water 10 times and 10 µL of the nanoconstructs solution was dropped onto a surface of TEM grid (Ted Pella, inc., Formvar/Carbon 400 mesh, Copper, approx. grid hole size: 42 µm). The efficiency of SPIOs loading was estimated by dissolving the loaded SiMPs in 0.1 M NaOH (99.99 % trace metals basis) overnight with further digestion in aqua regia. The samples were heated to dryness and were reconstituted in 2% HNO_3. The concentration of iron ions was measured using ICP-OES. The loading of the SPIOs within the mesoporous structure of the SiMPs was performed using several different batches of particles over the course of 8 months.

Supplementary References

1 Lopes, P. E., Murashov, V., Tazi, M., Demchuk, E. & MacKerell, A. D. Development of an empirical force field for silica. Application to the quartz-water interface. *J. Phys. Chem. B* **110**, 2782-2792 (2006).

2 Malani, A., Ayappa, K. & Murad, S. Influence of Hydrophilic Surface Specificity on the Structural Properties of Confined Water. *J. Phys. Chem. B* **113**, 13825-13839 (2009).

3 Lümmen, N. & Kraska, T. Investigation of the formation of iron nanoparticles from the gas phase by molecular dynamics simulation. *Nanotechnology* **15**, 525 (2004).

4 Soontrapa, C. & Chen, Y. Optimization approach in variable-charge potential for metal/metal oxide systems. *Comput. Mater. Sci.* **46**, 887-892 (2009).

5 Kumar, P., Han, S. & Stanley, H. E. Anomalies of water and hydrogen bond dynamics in hydrophobic nanoconfinement. *J. Phys.: Condens. Matter* **21**, 504108 (2009).

6 Nguyen, T. X. & Bhatia, S. K. Some anomalies in the self-diffusion of water in disordered carbons. *J. Phys. Chem. C* **116**, 3667-3676 (2012).

7 Gallo, P., Rovere, M. & Spohr, E. Supercooled confined water and the mode coupling crossover temperature. *Phys. Rev. Lett.* **85**, 4317-4320 (2000).

8 Mallamace, F. *et al.* Transport properties of supercooled confined water. *The European Physical Journal Special Topics* **161**, 19-33 (2008).

9 Allen, M. P. & Tildesley, D. J. *Computer simulation of liquids*. (Oxford university press, 1989).

10 Mark, P. & Nilsson, L. Structure and dynamics of the TIP3P, SPC, and SPC/E water models at 298 K. *J. Phys. Chem. A* **105**, 9954-9960 (2001).

11 Huang, R. *et al.* Direct observation of the full transition from ballistic to diffusive Brownian motion in a liquid. *Nat. Phys.* **7**, 576-580 (2011).

12 Mahadevan, T., Kojic, M., Ferrari, M. & Ziemys, A. Mechanisms of Reduced Solute Diffusivity at Nanoconfined Solid-Liquid Interface. *Chem. Phys.* **421**, 15-21 (2013).

13 Shlesinger, M. F., Zaslavsky, G. M. & Klafter, J. Strange kinetics. *Nature* **363**, 31-37 (1993).

14 Cui, S. Molecular self-diffusion in nanoscale cylindrical pores and classical Fick's law predictions. *J. Chem. Phys.* **123**, 054706 (2005).

15 Cicero, G., Grossman, J. C., Schwegler, E., Gygi, F. & Galli, G. Water confined in nanotubes and between graphene sheets: A first principle study. *J. Am. Chem. Soc.* **130**, 1871-1878 (2008).

16 Majolino, D., Corsaro, C., Crupi, V., Venuti, V. & Wanderlingh, U. Water diffusion in nanoporous glass: an NMR study at different hydration levels. *J. Phys. Chem. B* **112**, 3927-3930 (2008).

17 Hartnig, C. *et al.* Modifications of the hydrogen bond network of liquid water in a cylindrical SiO2 pore. *J. Mol. Liq.* **85**, 127-137 (2000).

18 Spohr, E., Hartnig, C., Gallo, P. & Rovere, M. Water in porous glasses. A computer simulation study. *J. Mol. Liq.* **80**, 165-178 (1999).

19 Kjelstrup, S. & Bedeaux, D. *Non-equilibrium thermodynamics of heterogeneous systems*. Vol. 16 (World Scientific Singapore, 2008).

20 Chen, S.-H. *et al.* The violation of the Stokes–Einstein relation in supercooled water. *Proc. Natl. Acad. Sci. USA* **103**, 12974-12978 (2006).

21 Rowlinson, J. J. S. & Widom, B. *Molecular theory of capillarity*. Vol. 8 (Courier Dover Publications, 2002).

22 Merabia, S., Shenogin, S., Joly, L., Keblinski, P. & Barrat, J.-L. Heat transfer from nanoparticles: A corresponding state analysis. *Proc. Natl. Acad. Sci. USA* **106**, 15113-15118 (2009).

23 Zang, J., Konduri, S., Nair, S. & Sholl, D. S. Self-diffusion of water and simple alcohols in single-walled aluminosilicate nanotubes. *ACS Nano* **3**, 1548-1556 (2009).

24 Milischuk, A. A. & Ladanyi, B. M. Structure and dynamics of water confined in silica nanopores. *J. Chem. Phys.* **135**, 174709-174711 (2011).

25 Liu, Y., Wang, Q., Wu, T. & Zhang, L. Fluid structure and transport properties of water inside carbon nanotubes. *J. Chem. Phys.* **123**, 234701 (2005).

26 Makarov, V. A., Feig, M., Andrews, B. K. & Pettitt, B. M. Diffusion of solvent around biomolecular solutes: a molecular dynamics simulation study. *Biophys. J.* **75**, 150-158 (1998).

27 Makarov, V., Pettitt, B. M. & Feig, M. Solvation and hydration of proteins and nucleic acids: a theoretical view of simulation and experiment. *Accounts of chemical research* **35**, 376-384 (2002).

28 Holten, V. & Anisimov, M. A. Entropy-driven liquid-liquid separation in supercooled water. *Sci. Rep.* **2** (2012).

29 Poole, P. H., Sciortino, F., Essmann, U. & Stanley, H. E. Phase behaviour of metastable water. *Nature* **360**, 324-328 (1992).

30 Angell, C. A. Insights into phases of liquid water from study of its unusual glass-forming properties. *Science* **319**, 582-587 (2008).

31 Nagoe, A., Kanke, Y., Oguni, M. & Namba, S. Findings of Cp Maximum at 233 K for the Water within Silica Nanopores and Very Weak Dependence of the Tmax on the Pore Size. *J. Phys. Chem. B* **114**, 13940-13943 (2010).

32 Asinari, P. Numerical prediction of turbulent convective heat transfer in mini/micro channels for carbon dioxide at supercritical pressure. *Int. J. Heat Mass Transfer* **48**, 3864-3879 (2005).

33 Swenson, J., Jansson, H. & Bergman, R. Relaxation Processes in Supercooled Confined Water and Implications for Protein Dynamics. *Phys. Rev. Lett.* **96**, 247802 (2006).

34 Brooks, R. A., Moiny, F. & Gillis, P. On T2-shortening by weakly magnetized particles: The chemical exchange model. *Magn. Reson. Med.* **45**, 1014-1020 (2001).

35 Gillis, P., Moiny, F. & Brooks, R. A. On T2-shortening by strongly magnetized spheres: A partial refocusing model. *Magn. Reson. Med.* **47**, 257-263 (2002).

36 Tong, S., Hou, S., Zheng, Z., Zhou, J. & Bao, G. Coating optimization of superparamagnetic iron oxide nanoparticles for high T2 relaxivity. *Nano Lett.* **10**, 4607-4613 (2010).

37 Pöselt, E. *et al.* Relaxivity Optimization of a PEGylated Iron-Oxide-Based Negative Magnetic Resonance Contrast Agent for T 2-Weighted Spin–Echo Imaging. *ACS Nano* **6**, 1619-1624 (2012).

38 Vuong, Q. L., Gillis, P. & Gossuin, Y. Monte Carlo simulation and theory of proton NMR transverse relaxation induced by aggregation of magnetic particles used as MRI contrast agents. *J. Magn. Reson.* **212**, 139-148 (2011).

39 Koenig, S. H. *et al.* Three types of physical measurements needed to characterize iron oxide nanoparticles for MRI and MRA: magnetization, relaxometry, and light scattering. *Acad. Radiol.* **9**, S5 (2002).

40 Kihara, K. An X-ray study of the temperature dependence of the quartz structure. *Eur. J. Mineral.* **2**, 63-77 (1990).

41 Ananta, J. S. *et al.* Geometrical confinement of gadolinium-based contrast agents in nanoporous particles enhances T1 contrast. *Nat. Nanotechnol.* **5**, 815-821 (2010).

42 Blake, A., Schröder, M. & Sykes, A. *Advances in inorganic chemistry*. Vol. 35 (Academic, New York, 1990).

43 Okudera, H., Kihara, K. & Matsumoto, T. Temperature dependence of structure parameters in natural magnetite: single crystal X-ray studies from 126 to 773 K. *Acta Crystallogr. Sect. B-Struct. Sci.* **52**, 450-457 (1996).

44 Yapo-Kicho, D., Lagant, P. & Vergoten, G. The SPASIBA Force Field for Studying Iron-Tannins Interactions: Application to Fe3+/Fe2+ Catechol Complexe. *Int. J. Mol. Sci.* **8**, 259-272 (2007).

45 Thorek, D. L., Chen, A. K., Czupryna, J. & Tsourkas, A. Superparamagnetic iron oxide nanoparticle probes for molecular imaging. *Ann. Biomed. Eng.* **34**, 23-38 (2006).

46 Spagnoli, D., Gilbert, B., Waychunas, G. A. & Banfield, J. F. Prediction of the effects of size and morphology on the structure of water around hematite nanoparticles. *Geochim. Cosmochim. Acta* **73**, 4023-4033 (2009).

47 Ellis, R. J. & Minton, A. P. Cell biology: Join the crowd. *Nature* **425**, 27-28 (2003).

48 Zhou, H.-X., Rivas, G. & Minton, A. P. Macromolecular crowding and confinement: biochemical, biophysical, and potential physiological consequences. *Annu. Rev. Biophys.* **37**, 375 (2008).

49 Bouchoux, A. *et al.* Molecular Mobility in Dense Protein Systems: An Investigation through 1H NMR Relaxometry and Diffusometry. *J. Phys. Chem. B* **116**, 11744-11753 (2012).

50 Bizzarri, A. R. & Cannistraro, S. Molecular dynamics of water at the protein-solvent interface. *J. Phys. Chem. B* **106**, 6617-6633 (2002).

51 Ansari, A. *et al.* Protein states and proteinquakes. *Proc. Natl. Acad. Sci. USA* **82**, 5000-5004 (1985).

52 Barron, L. D., Hecht, L. & Wilson, G. The Lubricant of Life: A Proposal That Solvent Water Promotes Extremely Fast Conformational Fluctuations in Mobile Heteropolypeptide Structure. *Biochemistry* **36**, 13143-13147 (1997).

53 Goldanskii, V. I. & Krupyanskii, Y. F. Protein and protein-bound water dynamics studied by Rayleigh scattering of Mössbauer radiation (RSMR). *Quarterly Reviews of Biophysics* **22**, 39-92 (1989).

54 Green, J. L., Fan, J. & Angell, C. A. The protein-glass analogy: New insight from homopeptide comparisons. *The Journal of Physical Chemistry* **98**, 13780-13790 (1994).

55 Denisov, V. P. & Halle, B. Protein hydration dynamics in aqueous solution. *Faraday Discussions* **103**, 227-244 (1996).

56 Otting, G., Liepinsh, E. & Wuthrich, K. Protein hydration in aqueous solution. *Science* **254**, 974-980 (1991).

57 Tarek, M. & Tobias, D. J. The dynamics of protein hydration water: a quantitative comparison of molecular dynamics simulations and neutron-scattering experiments. *Biophys. J.* **79**, 3244-3257 (2000).

58 Gallagher, T., Alexander, P., Bryan, P. & Gilliland, G. L. Two crystal structures of the B1 immunoglobulin-binding domain of streptococcal protein G and comparison with NMR. *Biochemistry* **33**, 4721-4729 (1994).

59 Vijay-Kumar, S., Bugg, C. E. & Cook, W. J. Structure of ubiquitin refined at 1.8 Å resolution. *J. Mol. Biol.* **194**, 531-544 (1987).

60 Kamata, K., Mitsuya, M., Nishimura, T., Eiki, J.-i. & Nagata, Y. Structural basis for allosteric regulation of the monomeric allosteric enzyme human glucokinase. *Structure* **12**, 429-438 (2004).

61 Barondeau, D. P., Putnam, C. D., Kassmann, C. J., Tainer, J. A. & Getzoff, E. D. Mechanism and energetics of green fluorescent protein chromophore synthesis revealed by trapped intermediate structures. *Proc. Natl. Acad. Sci. USA* **100**, 12111-12116 (2003).

62 Zhang, F. *et al.* Crystal structure of the obese protein Ieptin-E100. *Nature* **387**, 206-209 (1997).

63 Artymiuk, P. J., Blake, C. C. F., Rice, D. W. & Wilson, K. S. The structures of the monoclinic and orthorhombic forms of hen egg-white lysozyme at 6 A resolution. *Acta Crystallographica Section B* **38**, 778-783 (1982).

64 Scouloudi, H. & Baker, E. X-ray crystallographic studies of seal myoglobin: The molecule at 2.5 Å resolution. *J. Mol. Biol.* **126**, 637-660 (1978).

65 Xu, C., Rice, W. J., He, W. & Stokes, D. L. A structural model for the catalytic cycle of Ca2+-ATPase. *J. Mol. Biol.* **316**, 201-211 (2002).

66 Dresselhaus, M., Dresselhaus, G., Charlier, J. & Hernandez, E. Electronic, thermal and mechanical properties of carbon nanotubes. *Philosophical Transactions of the Royal*

Society of London. Series A: Mathematical, Physical and Engineering Sciences **362**, 2065-2098 (2004).

67 Marconnet, A. M., Panzer, M. A. & Goodson, K. E. Thermal conduction phenomena in carbon nanotubes and related nanostructured materials. *Reviews of Modern Physics* **85**, 1295 (2013).

68 Kolesnikov, A. I. *et al.* Anomalously Soft Dynamics of Water in a Nanotube: A Revelation of Nanoscale Confinement. *Phys. Rev. Lett.* **93**, 035503 (2004).

69 Chou, C.-C. *et al.* Single-Walled Carbon Nanotubes Can Induce Pulmonary Injury in Mouse Model. *Nano Lett.* **8**, 437-445 (2008).

70 Tajkhorshid, E. *et al.* Control of the Selectivity of the Aquaporin Water Channel Family by Global Orientational Tuning. *Science* **296**, 525-530 (2002).

71 Nanok, T., Artrith, N., Pantu, P., Bopp, P. A. & Limtrakul, J. Structure and dynamics of water confined in single-wall nanotubes. *J. Phys. Chem. A* **113**, 2103-2108 (2008).

72 Schuler, L. D., Daura, X. & van Gunsteren, W. F. An improved GROMOS96 force field for aliphatic hydrocarbons in the condensed phase. *J. Comput. Chem.* **22**, 1205-1218 (2001).

73 Lervik, A., Bresme, F., Kjelstrup, S., Bedeaux, D. & Miguel Rubi, J. Heat transfer in protein-water interfaces. *Phys. Chem. Chem. Phys.* **12**, 1610-1617 (2010).

74 Mu, Kosov, D. S. & Stock, G. Conformational Dynamics of Trialanine in Water. 2. Comparison of AMBER, CHARMM, GROMOS, and OPLS Force Fields to NMR and Infrared Experiments. *J. Phys. Chem. B* **107**, 5064-5073 (2003).

75 Chiavazzo, E. & Asinari, P. Enhancing surface heat transfer by carbon nanofins: towards an alternative to nanofluids? *Nanoscale Res. Lett.* **6**, 1-13 (2011).

76 Quo, Y., Karasawa, N. & Goddard, W. A. Prediction of fullerene packing in C60 and C70 crystals. *Nature* **351**, 464-467 (1991).

77 Walther, J. H., Jaffe, R., Halicioglu, T. & Koumoutsakos, P. Carbon nanotubes in water: structural characteristics and energetics. *J. Phys. Chem. B* **105**, 9980-9987 (2001).

78 Adriani, G. *et al.* The preferential targeting of the diseased microvasculature by disk-like particles. *Biomaterials* **33**, 5504-5513 (2012).
